# Revealing the Fate of Isomeric Monounsaturated Fatty Acids in *Enterococcus faecalis* Membrane Lipids and Their Influence on Antimicrobial Susceptibility

**DOI:** 10.1021/acsinfecdis.6c00092

**Published:** 2026-05-08

**Authors:** Rebekah L. Casey, Annika L. Silverberg, Michael T. Marty, Kelly M. Hines

**Affiliations:** † Department of Chemistry, 1355 University of Georgia, Athens, Georgia 30602, United States; ‡ Department of Chemistry and Biochemistry, 12330 University of Arizona, Tucson, Arizona 85721, United States; § Department of Chemistry, University of Texas at Austin, Austin, Texas 78712, United States

**Keywords:** Lipidomics, lipid isomers, isotope labeling, OzID, *Enterococcus faecalis*, daptomycin

## Abstract

Previous work suggests that *Enterococcus faecalis* uses exogenous oleic acid (FA 18:1­(*9z*)) to increase its tolerance against the membrane-targeting antimicrobial, daptomycin. However, the specific roles of OA and the endogenous positional isomer, *cis*-vaccenic acid (FA 18:1­(*11z*)), in both daptomycin-susceptible (Dap-S) and daptomycin-resistant (Dap-R) strains have not been fully explored. We performed lipidomics using hydrophilic interaction liquid chromatography and reversed-phase liquid chromatography-ion mobility-mass spectrometry to identify alterations in the lipid composition of Dap-S (S613) and Dap-R (R712) strain pairs of *E. faecalis* following OA or CV supplementation. Lipidomics showed that total PG intensity in Dap-S was only impacted by OA, while total DGDG intensity was impacted by both OA and CV. However, total PG and DGDG intensities in Dap-R were not impacted by either FA. Both strains produced significantly more lipids containing two 18:1 acyl tails following OA supplementation, while CV had a similar effect on Dap-S only, indicating that Dap-S and Dap-R respond to OA and CV differently. Additionally, the preferences for OA vs CV, alone and in mixtures, were distinguished using ozonolysis and deuterium-labeling techniques that can resolve C=C positional isomers. Growth and survival assays show that Dap-S responds differently to daptomycin when it is cultured with OA or CV. Together, these results reveal that *E. faecalis* strains with genetic determinants for daptomycin resistance respond differently, in membrane lipid composition and overall growth, to biologically relevant FA 18:1 isomers compared to strains that acquire daptomycin tolerance solely through exogenous fatty acid uptake.

## Graphical abstract

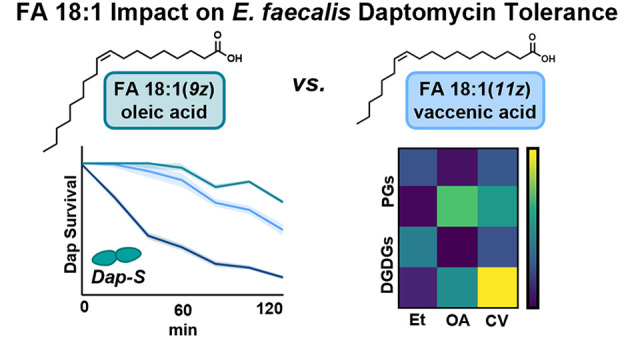

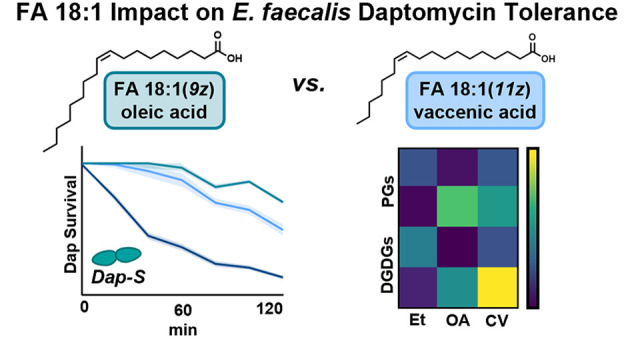

## Introduction

*Enterococcus faecalis* is a Gram-positive gut bacterium that, under certain circumstances, can cause life-threatening infections that affect many parts of the body, such as the bloodstream and skin.^1,2^ Since *E. faecalis* can have both natural and acquired resistance to multiple antibiotics designed to fight it, *E. faecalis* contributes to the ever-growing public health threat of antibiotic resistance.^3^ Daptomycin, an amphiphilic lipopeptide antibiotic, is used as a last-resort drug against Gram-positive bacteria that are resistant to more widely used antibiotics.^4,5^ The proposed mechanism of action of daptomycin is calcium-dependent and involves the insertion of the cationic daptomycin-Ca^2+^ complex into a portion of the bacterial membrane that is enriched in negatively charged phosphatidylglycerols (PGs) and lipid II.^6–9^ The daptomycin then oligomerizes in the membrane, leading to disruption of membrane polarization, leakage of ions and lipids from the cell, and activation of autolysis.^6–12^

Daptomycin-resistant (Dap-R) strains of *E. faecalis* are frequently found to have lower PG content in the membrane compared to susceptible strains, which reduces the ability of the cationic daptomycin-Ca^2+^ complex to bind membrane PGs.^13–15^ This phenotype is often achieved through mutations in genes that are involved in membrane lipid synthesis and cell envelope stress response.^13–17^ Allelic replacement experiments in the background of daptomycin-sensitive *E. faecalis* have demonstrated that the introduction of mutated *liaF*, a component of the *liaFSR* system for regulation of cell envelope stress response, increased the daptomycin MIC 4-fold but did not recapitulate the MIC of the resistant strain.^13,15^ However, the introduction of additional mutations found in cardiolipin synthase (*cls*) and glycerophosphoryl diester phosphodiesterase (*gdpD*), which further process phospholipid metabolites to generate glycerol-3-phosphate, fully replicated the MIC of the daptomycin-resistant strain.^13,15^ The combination of these mutations was shown to redistribute cardiolipins (CLs) away from the division septum to create anionic regions that trap daptomycin.^15,18^ Other alterations to membrane lipid composition that have been reported in Dap-R *E. faecalis* and *E. faecium* include elevated levels of mono- and diglucosyldiacylglycerols (MGDG and DGDG, respectively), which are precursors to lipoteichoic acid (LTA) synthesis.^14,15,19–20^

While genetic mutations are the drivers of daptomycin resistance, *E. faecalis* strains without genetically conferred resistance also have strategies to increase tolerance to daptomycin. One such strategy is to incorporate free fatty acids from the environment into its lipids to alter membrane properties, like fluidity and permeability, which can reduce daptomycin-induced membrane damage.^20–23,35^ *E. faecalis* uses the fatty acid kinase (*fak*) pathway, which has been extensively studied in *Staphylococcus aureus*, to attach exogenous free fatty acids or fatty acids liberated from host lipids to lipid glycerol backbones as fatty acyl tails through a variety of *fakA* and *fakB* proteins during lipid synthesis.^23–25^ It is possible that *E. faecalis* may uptake exogenous fatty acids more easily due to its unsaturated lipid nature,^19,14^ which causes more kinks and a less rigidly packed membrane.^26,27^ *E. faecalis* naturally produces *cis*-vaccenic acid (FA 18:1­(*11z*)), the most predominant unsaturated fatty acyl tail present across the lipid classes, but not its positional isomer, oleic acid (FA 18:1­(*9z*)). However, oleic acid has been observed to aid *E. faecalis* in its tolerance to the antibiotic daptomycin.^21,22,28,29^ In these studies, oleic acid incorporation has been evaluated primarily based on changes in the morphology of the bacteria, the sensitivity of the membrane to daptomycin in challenge assays, and lipid-class distributions.

While many studies have shown how oleic acid incorporation alters lipid classes and the FA 18:1 content of the membrane, the specific fate of incorporated FA 18:1­(*9z*) on glycerol backbones of phospholipids is not fully understood, since it can be difficult to distinguish the endogenous and exogenous FA 18:1 isomers with standard lipidomics.^21,22,28–30^ Additionally, while there have been numerous studies that investigate the impact of oleic acid on daptomycin-susceptible *E. faecalis*, how daptomycin-resistant *E. faecalis* responds to exogenous fatty acids is understudied.^20,21^ Evaluating exactly where FA 18:1 incorporates and how Dap-S and Dap-R *E. faecalis* respond differently to FA 18:1 isomers may provide a deeper understanding of how strains with different daptomycin susceptibilities may use the environment of infection sites differently. In this study, we compared the incorporation of exogenous FA 18:1­(*9z*) and FA 18:1­(*11z*) into daptomycin-susceptible (S613) and -resistant (R712) clinical isolate pairs of *E. faecalis*. Using hydrophilic interaction liquid chromatography (HILIC) and reversed-phase liquid chromatography (RPLC) with ion mobility-mass spectrometry (IM-MS),^31–34^ as well as techniques to distinguish the C=C isomers, we characterized the effects of exogenous FA 18:1 incorporation on lipid classes as a whole, individual lipids, and fragmented FA 18:1 fatty acyl tails. Through these analyses, we evaluated the fate of exogenous FA 18:1 in the membrane to reveal how Dap-S and Dap-R *E. faecalis* strains respond differently to the FA 18:1 isomers, contributing to the discussion of lipid and growth alterations in *E. faecalis* due to exogenous FA uptake.

## Results and Discussion

### Impacts of FA 18:1 Isomers on *E. Faecalis* Lipid Classes and Lipid Species

PGs are highly abundant in the membrane of daptomycin-susceptible (Dap-S) *E. faecalis* and are likely the entry point to the membrane for daptomycin.^19,35^ Daptomycin-resistant (Dap-R) strains have a reduced number of PGs, preventing daptomycin from negatively affecting the membrane. Since it has been observed that oleic acid incorporation can provide *E. faecalis* protection against daptomycin, it was our expectation that the total PGs in the membrane would be reduced primarily in our oleic acid-supplemented samples.^21,22,28,29^ A combination of RPLC- and HILIC-IM-MS was used to investigate PGs and DGDGs (SI Tables S2–S3) along with CLs, LysylPGs, and MGDGs, in a Dap-S (S613; note that the designation as susceptible is based on original reports on this isolate by Arias et al.^13^) and Dap-R (R712) *E. faecalis* strain pair when supplemented with oleic or *cis*-vaccenic acids. As in previous work, we detected a lower abundance of PGs, CLs, and LysylPGs and a higher abundance of DGDGs and MGDGs in R712 Et compared to S613 Et.^19^ When S613 was supplemented with oleic acid (S613 OA), the total amount of PGs was significantly reduced (0.45-fold, SI Tables S4 and S5) relative to the S613 control (S613 Et), while the total PG abundance in *cis*-vaccenic acid-supplemented S613 (S613 CV) remained similar to S613 Et (Figure 1A). No such net PG decrease was observed in R712 when it was supplemented with either OA or CV. The total amount of FA 18:1 fragmented from PGs increased in both strains when supplemented with either FA, even in S613 OA, where a decrease in total PG levels was observed (Figure 1B). Notably, there was a higher intensity of FA 18:1 fragmented from PGs when R712 was supplemented with OA compared to that of CV, but the opposite trend is observed for S613, suggesting that the strains incorporate the two FAs into PGs differently. Investigation of the lipid changes at the level of individual species (Figure 2) revealed a more complex scenario in which the abundance of most PG species was significantly reduced in the OA-treated S613 and R712. However, this occurred with a concomitant increase in PG species that presumably contained FA 18:1-derived acyl tails, i.e., PG 36:2 and PG 37:2, which account for the net increase in FA 18:1 acyl tail fragment intensity from the PG pool. This increase in PG 36:2 and 37:2 was substantial enough to balance the decrease in other PG species in R712 but not in S613. Given the dependency of daptomycin on anionic membrane lipids,^6,7,36^ the OA-induced reduction in PGs is a likely contributor to the observations of OA-induced daptomycin tolerance in *E. faecalis*.^20,22,23,29^

**Figure 1:**
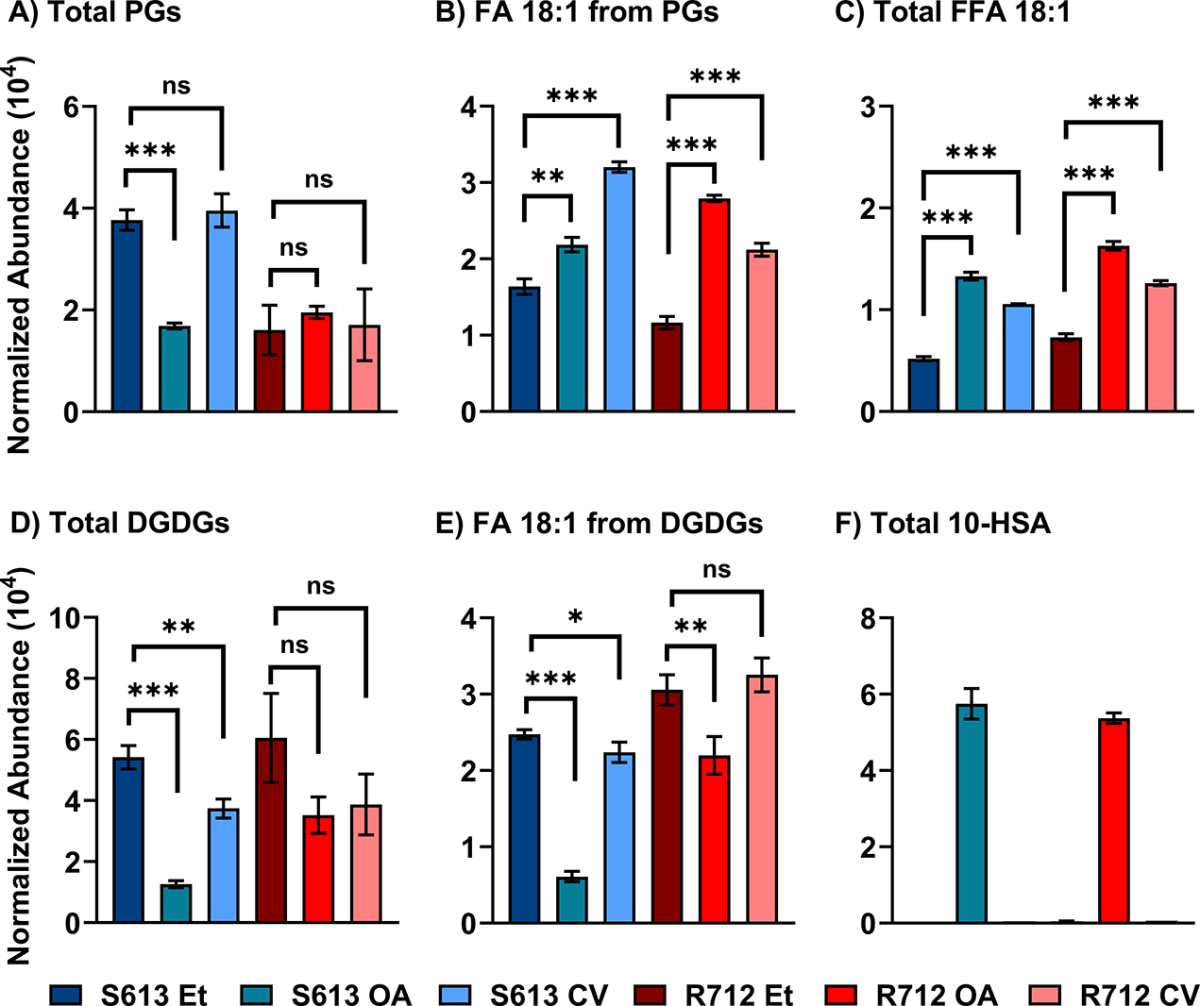
RPLC- and HILIC-IM-MS analysis of S613 and R712 and the impact of FA 18:1 isomers, oleic acid (OA) and *cis*-vaccenic acid (CV), have on: A) the total PG abundance, B) the total intensity of FA 18:1 fragmented from PGs, C) the total FFA 18:1, D) the total DGDG abundance, E) the total intensity of FA 18:1 fragmented from DGDGs, and F) the intensity of 10-hydroxystearic acid. Note: false discovery rate-adjusted multiple unpaired *t*-test *p*-values where *<0.05, **<0.01, ***<0.001.

**Figure 2:**
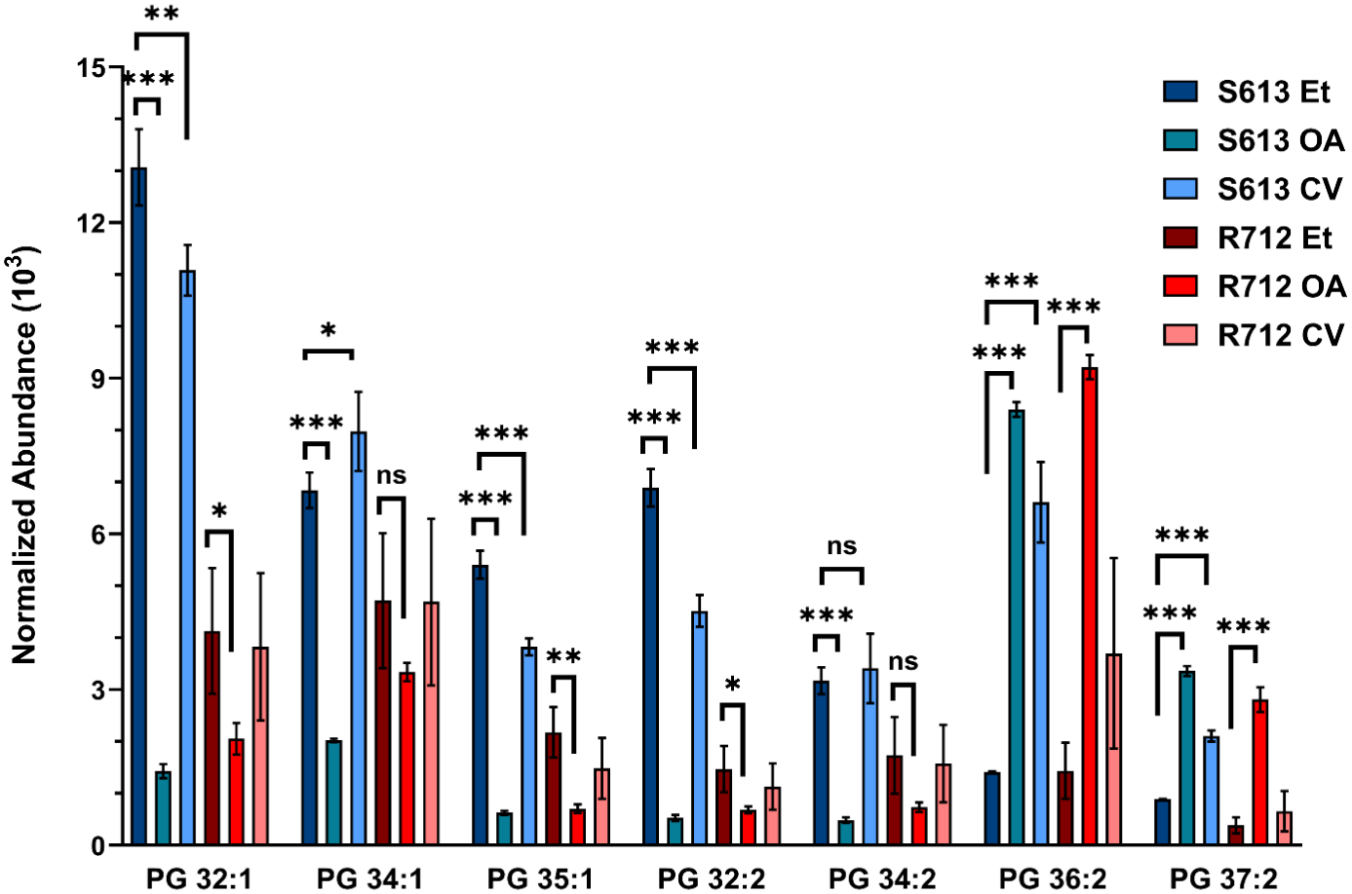
PG abundance changes observed in RPLC-IM-MS of S613 and R712 supplemented with FA 18:1 isomers, oleic acid (OA) or *cis*-vaccenic acid (CV), or an equivalent volume of ethanol (Et). Note: false discovery rate-adjusted multiple unpaired *t*-test *p*-values where *<0.05, **<0.01, ***<0.001. Comparisons of R712 Et with R712 were all not statistically significant. All *p*-values and log_2_ fold-change results are provided in SI Table S7–S8.).

The other major anionic phospholipid in *E. faecalis* is cardiolipin, which is synthesized directly from two PG precursors by cardiolipin synthase (*cls*). As with PGs, we found that CV supplementation had little impact on the total S613 and R712 CLs (SI Figure S2A). However, OA supplementation resulted in an increase in CLs for both S613 and R712 compared to the Et controls (SI Table S6), which was driven by higher amounts of CL containing 18:1-derived acyl tails, i.e., CL 72:4 (SI Figure S3A). Several studies have reported increased levels of CLs and mutations in *cls* within Dap-R strains of *E. faecalis*.^13,37^ The elimination of cardiolipin synthesis in *E. faecalis* sensitizes the bacteria to daptomycin, but the restoration of daptomycin tolerance in *cls* mutants after supplementation with oleic acid suggests that elevated CL levels are not the sole cause of oleic acid-induced daptomycin tolerance.^18,35^

*E. faecalis* also contains a neutral glycolipid, diglucosyldiacylglycerol (DGDG), within its membrane. Amounts of DGDG or related species, monoglucosyldiacylglycerol, or glycerophosphoryl DGDG (GPDGDG), have been reported to be elevated in Dap-R strains of *E. faecalis* and impacted by oleic acid supplementation.^14,35^ We observed that the total abundance of DGDG decreased significantly in S613 OA, while DGDGs in R712 OA and both strains supplemented with CV were only slightly reduced (Figure 1D). Unlike what was observed from the PG fragmentation, the intensity of the FA 18:1 fragment measured at the DGDG HILIC retention time in S613 OA was significantly lower than that of the control, while the intensity of the DGDG-derived FA 18:1 fragment from CV-treated S613 and R712 decreased slightly or remained consistent with control conditions (Figure 1E). These trends were driven by a dramatic reduction in nearly all DGDG species within S613 following growth with OA, as shown in Figure 3. Although DGDG species with 36:2 and 37:2 acyl tail compositions increased slightly upon OA supplementation, the impact was not sufficient to counteract the reduction in other DGDG species in S613 treated with OA. Decreases in DGDG species were also observed in CV-supplemented S613 and R712, but the impact of CV on DGDGs was less significant than that of OA.

**Figure 3:**
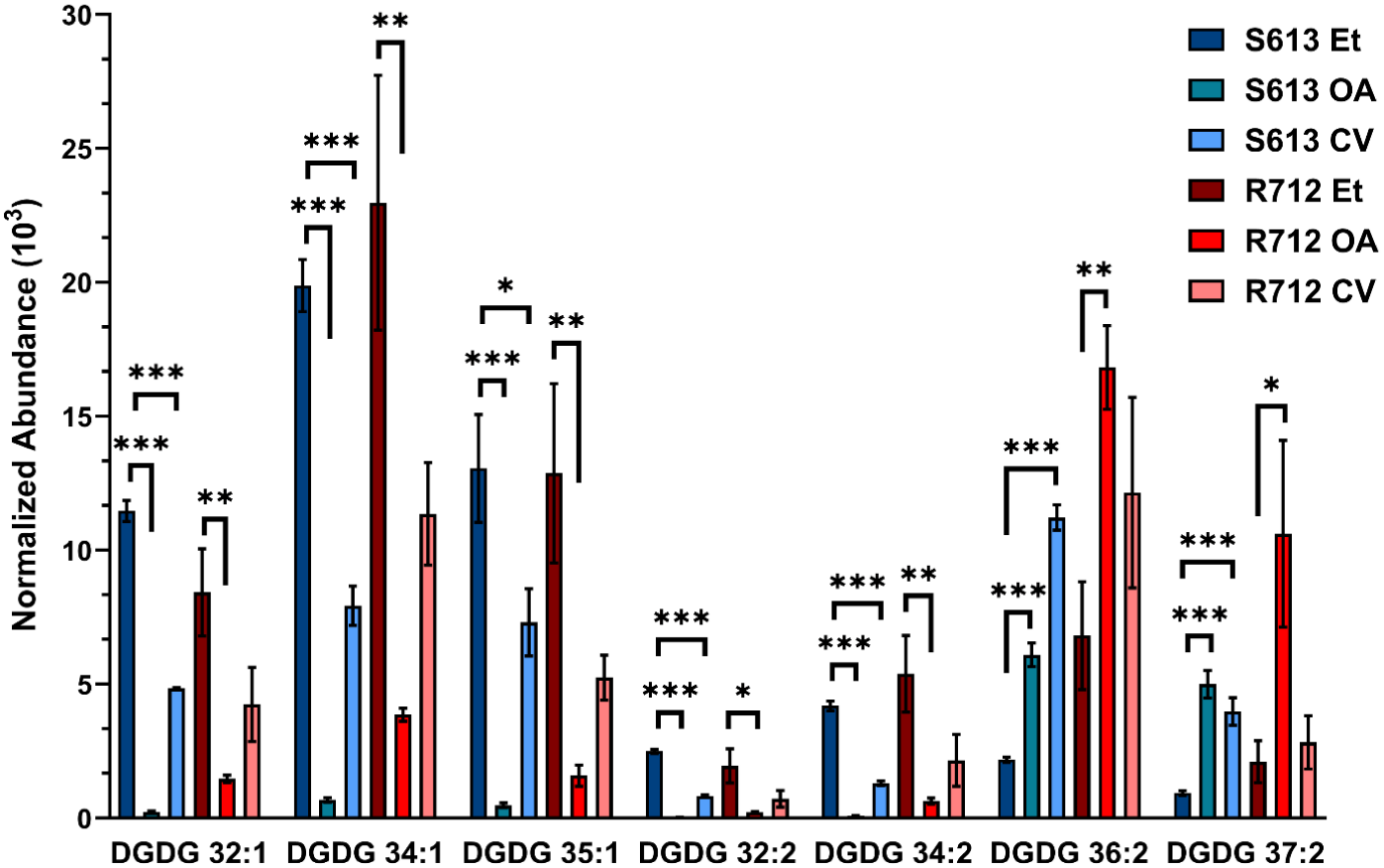
DGDG abundance changes observed in RPLC-IM-MS of S613 and R712 supplemented with FA 18:1 isomers, oleic acid (OA) or *cis*-vaccenic acid (CV), or an equivalent volume of ethanol (Et). Note: false discovery rate-adjusted multiple unpaired *t*-test *p*-values where *<0.05, **<0.01, ***<0.001. Comparisons of R712 Et with R712 were all not statistically significant. All *p*-values and log_2_ fold-change results are provided in SI Table S7–S8.).

Other significant impacts were detected in minor lipid components of the *E. faecalis* membrane. Total levels of the cationic lipid species, LysylPGs, decreased nearly 10-fold in S613 upon OA treatment (SI Figure S2B, SI Table S6), whereas CV supplementation resulted in just a 2.5-fold reduction. The observation of LysylPG in S613 OA is almost entirely attributable to LysylPG 36:2 (SI Figure S3B), which was the most abundant species by several orders of magnitude and was completely absent from the S613 Et control. In R712, supplementation with CV had little impact on total or individual LysylPG species. However, OA supplementation in R712 also resulted in a significant amount of LysylPG 36:2. Although R712 has a higher baseline amount of MGDGs than S613, no significant differences were detected in the total amount of MGDGs upon supplementation of S613 or R712 with CV or OA (SI Figure 2C). The lack of an effect from OA on total levels was likely the result of both elevated MGDGs 36:2 and 37:2 and reduced levels of other species in both strains (SI Figure S3C). CV supplementation also increased MGDG 36:2 in S613 but did not have a significant effect on R712.

Lastly, we observed that the amount of free FA 18:1 (FFA 18:1) was significantly higher under the oleic acid- and *cis*-vaccenic acid-supplemented conditions than in the ethanol vehicle controls (Figure 1C). We also detected a product of oleic acid, 10-hydroxystearic acid (10-HSA), in the extracts of S613 and R712 that were supplemented with oleic acid (Figure 1F). The formation of 10-HSA occurs through the action of oleate hydratase (OhyA), which catalyzes the addition of water to the double bond of oleic acid to form 10-HSA in a stereospecific reaction.^38–41^ Because of this process, the production of 10-HSA competes with the amount of free OA that is available for lipid synthesis.

On the basis of these results, OA has a larger impact on the total PG and DGDG abundances of S613 than CV, while OA and CV have a similar effect on the total PG and DGDG abundances of R712. The increased intensity of the FA 18:1 acyl tail fragment ion from the PGs, despite the overall decrease in PG abundance, and the lack of an increase for DGDGs, suggest that more of the supplemented FA 18:1 incorporates into PGs than DGDGs for both strains. Although some oleic acid was converted to 10-HSA, both strains contained more FFA 18:1 when OA was provided than when it was provided with CV. These differences in FFA levels may also explain why FA 18:1 fragmented from PGs and DGDGs is lower in S613 OA than that observed in S613 CV. Collectively, these results show that both Dap-S and Dap-R synthesize long, doubly unsaturated lipids (i.e., 36:2/37:2) when exogenous oleic acid or excess *cis*-vaccenic acid is available in their environment. However, it appears that R712 is better able to resist the influence of OA on the PGs and DGDGs that are natively produced by *E. faecalis*, whereas the amounts of PGs and DGDGs in S613 are negatively impacted by OA. These lipidomic results demonstrate that Dap-S and Dap-R respond to the FA 18:1 isomers differently at the levels of the lipid class and individual lipid species, suggesting that their response to OA-mediated daptomycin tolerance may also differ.

### Patterns of Fatty Acyl Tail Composition in PGs and DGDGs Based on Carbon Length and Degree of Unsaturation

The location of fatty acyl tails on the glycerol backbone is an important indication of membrane fluidity and the level of control the membrane can have over environmental stressors.^42^ To begin to understand which fatty acyl tail position oleic and *cis*-vaccenic acid tend to incorporate at, and whether there is any variation in the fatty acyl tail distributions between Dap-S and Dap-R lipids due to their response to the supplemented FAs, we examined the fatty acyl fragmentation patterns in the data-independent MS/MS spectra of the two main lipid classes, PGs and DGDGs. However, coelution between the monounsaturated and doubly unsaturated PGs and DGDGs in the RPLC separation created difficulty in distinguishing the amount of FA 18:1 coming from each lipid species using the data-independent MS/MS strategy (SI Figure S4). To combat this, all lipid species suspected to contain FA 18:1-derived acyl tails were selected for additional targeted MS/MS analysis to determine the abundance of FA 18:1 within lipid species and its position on the glycerol backbone.

The targeted MS/MS analysis revealed that only PGs 32:1 and 32:2 (Figures 4A and SI Figure S5D), as well as DGDGs 32:1 and 32:2 (Figures 4E and SI Figure S7D), contained evidence of a second fatty acyl tail combination that did not include FA 18:1. The addition of exogenous oleic acid, but not *cis*-vaccenic acid, suppressed the overall abundance of the 32:1 and 32:2 lipid species, and only the fatty acyl tail combinations that included FA 18:1 could be detected. We initially suspected that the specific positions of the acyl tails could be altered by FA 18:1 supplementation based on the significant alterations seen in each of the lipid classes. However, targeted RPLC-IM-MS/MS spectra of PGs and DGDGs (SI Figures S6 and S8) revealed that FA acyl tail positions did not change between conditions but depended on lipid class and fatty acyl tail compositions. The intensity of the fatty acyl fragment ions in MS/MS spectra generated by CID of phospholipids is determined by their position on the glycerol backbone, where the acyl tail at the *sn-2* position of phospholipids is more labile and produces a higher-intensity signal than the acyl tail from the *sn-1* position.^43–47^ In the case of monounsaturated PGs, we observed that the saturated fatty acid was consistently the more abundant fatty acyl tail fragment (Figure 4A-B). This suggested that the saturated acyl tail was located at the *sn-2* position, and FA 18:1 occupied the *sn-1* position. For the doubly unsaturated PGs, including PG 32:2 and 34:2, the more abundant fragment corresponded to the shorter acyl tail, while the FA 18:1 acyl tail occupied the *sn-1* position (Figures 4C and SI Figure S5D-E). Both PG 36:2 and PG 37:2 contained FA 18:1-derived acyl tails at both the *sn* positions. In PG 37:2, the *sn-2* position was found to contain FA 18:1, while the *sn-1* position contained a product of FA 18:1, FA 19:1 (cyclo), which is formed by the addition of a methylene group across the carbon–carbon double bond by cyclopropane synthase (Figures 4D and SI Figure S5F-G).^23,30^ In contrast to PGs, the fatty acyl tail on the *sn-1* position of the DGDG headgroup reportedly produces the more intense peak when fragmented.^48^ The fragmentation of monounsaturated DGDGs revealed a pattern in which the unsaturated fatty acyl tail fragment had the higher intensity (Figures 4E and SI Figure S7A−C), indicating that FA 18:1 occupied the *sn-1* position, like what was observed for monounsaturated PGs. For the doubly unsaturated DGDGs, FA 18:1 was located on the *sn-1* position of DGDG 32:2 and 34:2 (SI Figure 7D-E) and the *sn-2* position for DGDG 37:2 (Figure 4H).

**Figure 4:**
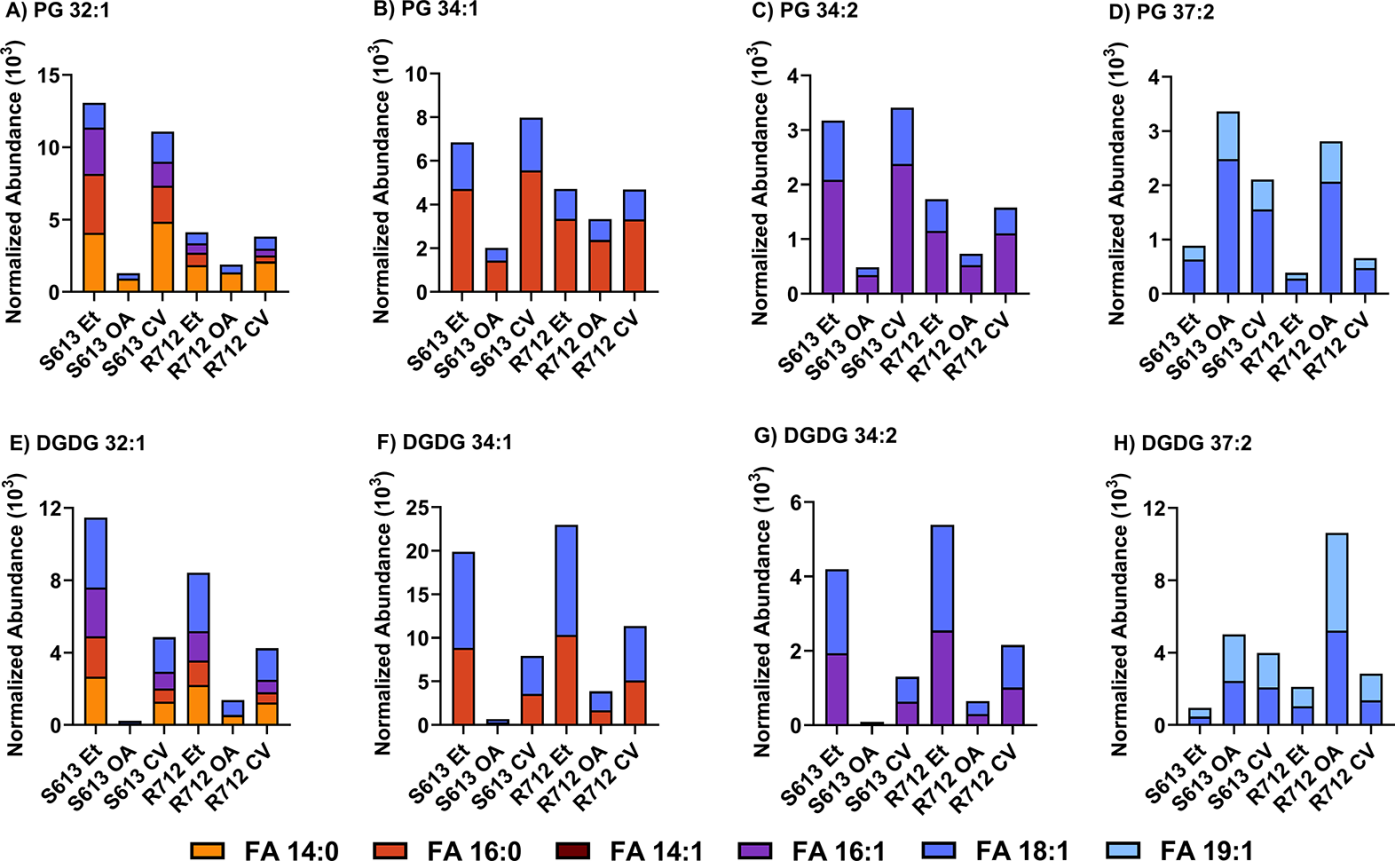
Fatty acyl tail fragment ion distributions from targeted RPLC-IM-MS/MS of: A) PG 32:1, B) PG 34:1, C) PG 34:2, D), PG 37:2, E) DGDG 32:1, F) DGDG 34:1, G) DGDG 34:2, and H) DGDG 37:2. The ratios were calculated from the intensities of all fatty acyl tail fragment ions detected in the MS/MS spectra and scaled to the normalized abundance of each lipid species.

These results suggest that the backbone positions of the fatty acyl tails depend on their degree of unsaturation for monounsaturated lipids and the carbon tail length for doubly unsaturated lipids. For all PGs and DGDGs, besides 36:2 and 37:2, the endogenous and incorporated FAs 18:1 were assigned to the *sn-1* position of the glycerol backbone based on the relative fragment intensities. Interestingly, the only lipids that had FA 18:1, or FA 18:1-derived FA 19:1, on the *sn-2* position were the four lipids that significantly increased upon the addition of oleic acid and, for S613 only, *cis*-vaccenic acid. In the oleic acid-supplemented conditions, it is possible that PGs and DGDGs with 36:2 and 37:2 compositions contained oleic acid, either directly or after cyclopropyl modification to FA 19:1, at both *sn* positions or a mixture of oleic acid with endogenous *cis*-vaccenic acid.

### Preference for Oleic Acid Incorporation During Lipid Synthesis

As presented in the previous sections, oleic acid supplementation affected PG and DGDG abundances more than *cis*-vaccenic acid supplementation. To begin to discern if these differences were due to preferences for a specific fatty acid structure, a mixture of both isomers (50 μM OA and 50 μM CV; 100 μM FA in total) was provided in the culture media for S613 and R712. The total lipid class abundances when the strains were supplemented with a mix of both isomers (S613 OA/CV and R712 OA/CV) were analyzed with HILIC-IM-MS (SI Table S11, SI Table S14). It was clear that the total PGs and DGDGs in S613 OA/CV (SI Figure S9, SI Table S12–13) were consistent with the OA-only condition, a trend that can also be observed in the total LysylPGs (SI Figure S10, SI Table S15). Since there was a similar impact on R712 total lipid classes when provided OA and CV separately, it is difficult to relate the total lipid class intensities of R712 OA/CV to one of the separate FA conditions. Additionally, when S613 was provided with the OA and CV mixture, individual PGs and DGDGs were reduced less significantly than what was observed in the OA-only condition (SI Figure S11, SI Table S16–17), presumably because the concentration in the mixture was half that of the OA-only treatment. In R712, the impact of the OA/CV mixture differed between the PGs and DGDGs. While the PGs of R712 were reduced when grown with the OA/CV mixture (SI Figure S11A), similar to but less significantly than the impact from OA alone, DGDG 36:2 and 37:2 responded to the OA/CV mixture in a manner that was more similar to the CV-only condition (SI Figure S11B). Both R712 and S613 OA/CV CLs seem to have similar trends as the OA condition, while it is unclear whether S613 and R712 OA/CV LysylPGs, in which there is a notable absence of LysylPG 36:2, and MGDGs follow the separate FA conditions (SI Figure S12, Table S18–19). However, the exact amount of OA vs CV incorporation in the mixed OA/CV conditions, or the amount of OA incorporated in addition to the background levels of endogenous CV in the OA conditions, cannot be determined using standard RPLC-IM-MS lipidomics methods.

To confirm the incorporation of OA and CV into *E. faecalis* lipids, we performed ozone-induced dissociation (OzID) to determine the specific carbon–carbon (C=C) double bond positions within the fatty acyl tails of unsaturated PGs and DGDGs that were detected in the lipid extracts of all FA-supplemented growth conditions (i.e., +CV, +OA, and +OA/CV). In OzID, ozone reacts with the lipid double bond to form an unstable ozonide that fragments to reveal the specific location of the C=C bond (SI Figures S13 and S14). These diagnostic fragment ions, known as the Criegee and aldehyde fragments, are 16 *m*/*z* apart, and their *m*/*z* values differ based on the location of the C=C bond relative to the ultimate carbon of the fatty acyl tail.^49–51^

Based on the FA compositions from RPLC-IM-MS and the OzID fragmentation spectra (SI Figures S15 and S17), we calculated the ratio of FA 18:1­(*9z*) to FA 18:1­(*11z*) for all the lipids detected in the FA-supplemented growth conditions of S613 and R712 (SI Tables S20 and S21). We found that more OA was incorporated into R712 PGs than S613 PGs (SI Figure S16) when it was the only exogenous FA provided to the bacteria. For PGs containing just one 18:1 acyl tail, the distribution of OA and CV was nearly identical between S613 and R712 when the bacteria were provided with the OA/CV mixture but was biased toward more CV than OA (63–94% CV). However, nearly all PG species containing two 18:1 or 18:1-derived acyl tails had at least one OA tail in S613 and R712 when only OA was provided in the broth, and 44–53% OA when provided with the OA/CV mixture. It should be noted that these results do not reflect the amount of 18:1 FA that is converted to 19:1 FA, as the cyclopropyl modification is not susceptible to ozonolysis. The results for DGDGs show trends similar to those of the *9z*/*11z* ratios found in PGs, but the amount of OA tended to be lower in DGDGs than in PGs (SI Figure S18). We also found that the intensities of several DGDG species (32:2 and 34:2) were too low to obtain *9z*/*11z* ratios for all FA-supplemented conditions. The competition between Criegee and aldehyde fragment formation, as well as the competition from multiple C=C sites within a lipid, can present challenges to the detection of diagnostic OzID fragment ions from low-abundance lipid precursors.

To more accurately reflect the total amount of oleic acid incorporated into lipids, including the incorporation of two oleic acids within the same lipid and the presence of oleic-acid-derived FA 19:1, the same experiments were repeated using deuterated oleic acid. The lipids with endogenous CV and incorporated *d*_9_-OA were detected with HILIC-IM-MS (SI Table S22), and the ratios of PGs and DGDGs with one, two, or no *d*_9_-OA were calculated (SI Table S23, SI Table S24). Results from the deuterated OA experiments showed that monounsaturated PGs (Figures 5A and SI Figure S19) and DGDGs (Figures 5D and SI Figure S20) contained 33–50% oleic acid in the S613 OA condition and 43–63% oleic acid in the R712 OA condition. When deuterated oleic acid was provided in a mixture with *cis*-vaccenic acid, the amount of incorporated OA dropped to 27–50% in the monounsaturated PGs and DGDGs. PGs 32:2 and 34:2 (SI Figure S19) and DGDG 34:2 (SI Figure S20), which are expected to contain just one 18:1 FA, followed similar incorporation trends as the monounsaturated PGs and DGDGs (i.e., 18–74% deuterated oleic acid in OA and 18–49% in OA/CV).

**Figure 5:**
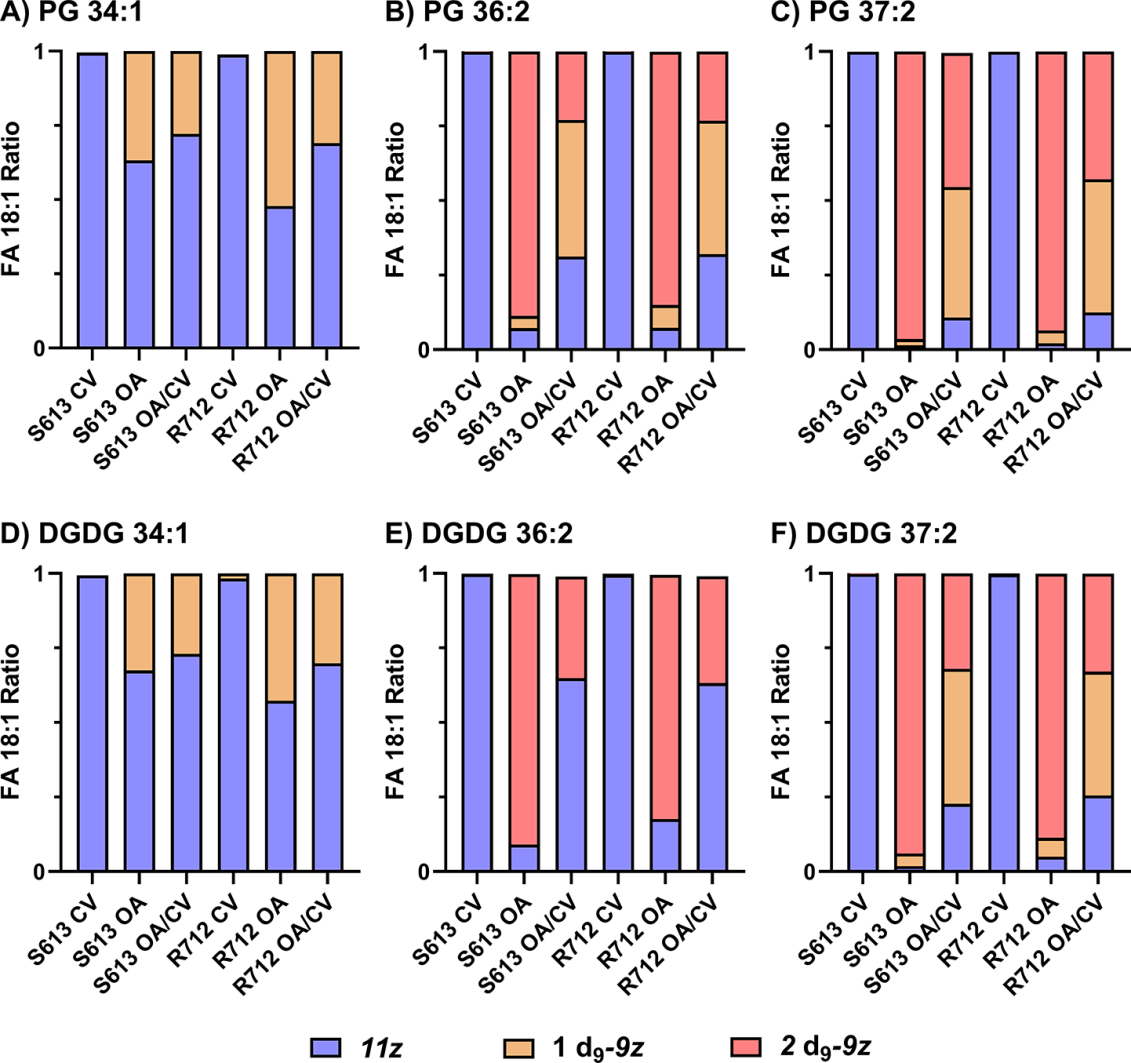
Distribution of FA 18:1­(*11z*) and incorporated *d*_9_-FA 18:1­(*9z*) utilized in the production of: A) PG 34:1, B) PG 36:2, C) PG 37:2, D) DGDG 34:1, E) DGDG 36:2, and F) DGDG 37:2. FA 18:1 isomer ratios were calculated from the relative intensities of unlabeled lipids and lipids with one or two labeled fatty acyl tails in HILIC-IM-MS data.

The incorporation of deuterated oleic acid was more heterogeneous in the PGs and DGDGs containing two unsaturated acyl tails. The long-chain doubly unsaturated PGs, PG 36:2 and PG 37:2 (Figure 5B–C), were nearly all *d*_9_-oleic acid in the OA-only conditions of both S613 and R712, suggesting that these lipid species were synthesized almost exclusively with exogenous *d*_9_-oleic acid at both *sn* positions. When S613 and R712 were provided with mixtures of OA and CV, the amount of *d*_9_-oleic acid incorporation in PG 36:2 and PG 37:2 decreased, and the amount with mixed OA and CV-derived acyl tails increased. Unlike the monounsaturated species, where incorporation trends were similar between PGs and DGDGs, we observed significant differences in the acyl tail compositions between DGDG 36:2 and PG 36:2. Our results indicated that DGDG 36:2 was either fully derived from *d*_9_-oleic acid or fully derived from *cis*-vaccenic acid when both FAs were provided (Figure 5E). This trend was observed for DGDG 36:2 in both S613 and R712. However, DGDG 37:2 (Figure 5F) mirrored the composition of PG 37:2, in which species containing two *d*_9_-oleic acid-derived tails and species with a mixture of *d*_9_-oleic and *cis*-vaccenic acyl tails were both detected.

Looking cumulatively at the ratio of FA 18:1*(11z)* vs FA 18:1*(9z)* from this experiment (SI Table S25, SI Figure S21), it is clear that a significant portion of the PG (SI Figure S22A) and DGDG (SI Figure S22B) lipids still contain 26–36% *cis*-vaccenic acid (*11z*) even when only *d*_9_-oleic acid is provided to the cultures. Interestingly, the PGs of R712 retain less (26%) *cis*-vaccenic acid than S613 (32%) when *d*_9_-oleic acid is provided alone, while the distributions are nearly identical (52%) when both *cis*-vaccenic and *d*_9_-oleic acids are supplemented in the broth. On the other hand, the DGDGs of R712 contain slightly more (36%) *cis*-vaccenic acid than S613 (32%) when *d*_9_-oleic acid is provided. R712 also appears to form more PGs and DGDGs with just a single *d*_9_-oleic acid than S613 when *d*_9_-oleic is the only exogenous FA provided in the culture, while S613 appears to form more PGs and DGDGs with two *d*_9_-oleic acid tails (46–57%). Our interpretation of these observations is that oleic acid is distributed across a wider range of DGDG and PG species in R712, while much of the oleic acid is concentrated into PG 36:2 and DGDG 36:2 in the S613 strain. This is consistent with data from our previous experiments with unlabeled oleic acid, in which we observed that OA suppressed unsaturated PGs and DGDGs to a greater extent in S613 than in R712 (Figures 2 and 3).

In general, there was remarkable agreement between the OzID and deuterium-labeling experiments. Both data sets clearly demonstrate that a substantial amount of endogenous *cis*-vaccenic acid remains in the PGs and DGDGs of both S613 and R712 when grown in oleic acid. There is also clear agreement that a greater fraction of oleic acid is incorporated into monounsaturated PGs and DGDGs in R712 than in S613, even when oleic acid is provided in an equimolar mixture with *cis*-vaccenic acid. The relative distribution between oleic acid and *cis*-vaccenic acid in lipids with two 18:1 acyl tails (i.e., PG 36:2, DGDG 36:2) is also in agreement between the two data sets. However, the OzID data obscure the extent of oleic acid incorporation into both acyl tails of PGs and DGDGs. This is partially due to the conversion of the 18:1 C=C bond into a cyclopropyl moiety, which is not susceptible to ozonolysis (as in PG 37:2, DGDG 37:2), as well as the difficulty in detecting multiple OzID events within a single lipid species with multiple double bonds on the chromatographic time scale.^52–54^

### Oleic Acid and *cis*-Vaccenic Acid Support Daptomycin Tolerance in Dap-S *E. Faecalis*

Changes in the lipid composition of strains S613 and R712 led us to investigate whether the incorporation of exogenous oleic acid or *cis*-vaccenic acid could influence the tolerance of these strains to daptomycin. Several studies in *E. faecalis* have identified oleic acid incorporation as a protective mechanism against daptomycin, but only a few studies have investigated the impact on both daptomycin-susceptible and nonsusceptible strains.^20,21^ Growth curves for S613 and R712, performed in FA-supplemented broth, showed little impact of oleic acid or *cis*-vaccenic acid on the growth of S613 (SI Figure S23). The growth curve AUCs from the EtOH vehicle control and *cis*-vaccenic acid conditions were not significantly different for R712 either. However, the growth of R712 was reduced slightly by the presence of oleic acid (SI Figure S23, *t*-test *p* = 0.039 and 0.033). Minimum inhibitory concentration measurements determined that S613 growth was inhibited by 8 μg/mL daptomycin in BHI supplemented with CaCl_2_ and EtOH (note that these are not CLSI MICs and cannot be used to infer susceptibility or resistance relative to CLSI breakpoints). R712 growth was not completely inhibited until a daptomycin concentration of 512 μg/mL, but significant increases in the lag period (i.e., >3 h) were observed at 128–256 μg/mL (SI Figure S24).

Growth curves combining FA 18:1 supplementation in the presence of daptomycin were performed to determine if oleic acid or *cis*-vaccenic acid had impacts on S613 and R712 at low (1 and 4 μg/mL) and high (64 μg/mL) daptomycin concentrations (SI Figure S25). Little impact from daptomycin or the FA 18:1 isomers was detected during growth at 1 and 4 μg/mL of daptomycin. At both concentrations, a slightly higher OD was achieved during late-stationary growth of S613 when supplemented with oleic acid (SI Table S26) and the doubling times of S613 with OA were comparable to the doubling times of R712 (SI Table S27). However, all growth of S613 was inhibited by daptomycin at 64 μg/mL regardless of FA supplementation. To better investigate the influence of FA 18:1-mediated daptomycin tolerance at early time points, we performed survival assays on midexponential phase S613 cells that were treated with *cis*-vaccenic acid or oleic acid for 30 min prior to being challenged with an inhibitory concentration of daptomycin. As shown in Figure 6, oleic acid provided the greatest degree of protection against daptomycin for S613, but *cis*-vaccenic acid also provided a positive impact on the survival of S613.

**Figure 6:**
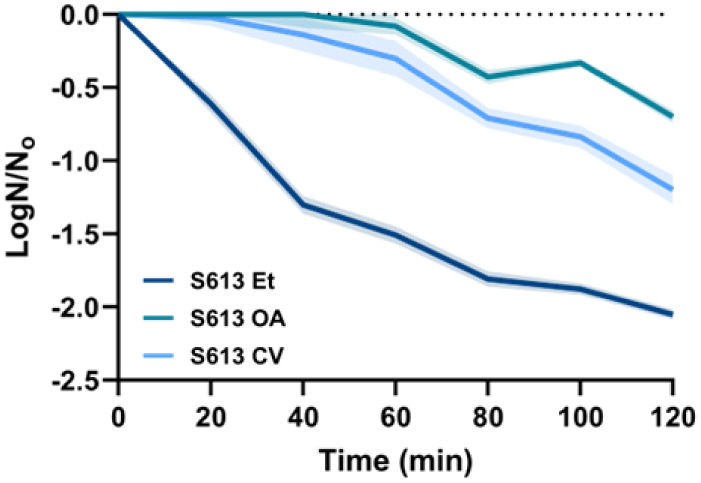
Daptomycin survival assay for S613 supplemented with OA, CV, or an equivalent volume of ethanol (Et). Cells were exposed to 64 μg/mL of daptomycin in BHI with 30 mg/L CaCl_2_. Aliquots were taken every 20 min for CFU/mL enumeration. Data represent three independent biological replicates.

## Conclusions

Previous studies on the influence of exogenous fatty acids on *Enterococcus faecalis* have demonstrated that certain FA structures are protective against membrane-damaging compounds, including the antimicrobial daptomycin.^20–22,28,29^ However, a key gap in these studies is whether *E. faecalis* strains with genetic determinants of daptomycin resistance further benefit from the influence of exogenous fatty acids. Additionally, the difficulty in separating FA 18:1 C=C isomers using traditional lipidomics is an inherent challenge in investigating FA 18:1 incorporation into *E. faecalis* lipids, as the bacteria- and host-derived versions of FA 18:1 differ in their C=C location. To address these gaps, we used a combination of chromatographic separations, tandem mass spectrometry, ozonolysis and deuterium-labeling techniques to investigate the impacts of the endogenous 18:1­(*11z*) fatty acid, *cis*-vaccenic acid, and the host-derived 18:1­(*9z*) fatty acid, oleic acid, in the well-characterized *E. faecalis* S613 and R712 clinical isolate pairs with daptomycin sensitivity and resistance, respectively.

While oleic acid had significant impacts on the lipid composition of both strains, the nature of the impact varied substantially between the daptomycin-sensitive S613 and the daptomycin-resistant R712 strains (Figure 7). The addition of OA to culture conditions suppressed the total abundance of PGs and DGDGs in S613, whereas the total abundance of PGs of R712 was unaffected, and DGDG levels were slightly (but not significantly) reduced. These results are consistent with work by the Cronan group demonstrating that oleic acid strongly represses *de novo* fatty acid biosynthesis in *E. faecalis* by preventing the transcription of fatty acid biosynthesis machinery encoded in the *fab* operon.^55^ However, *cis*-vaccenic acid is similarly able to repress *de novo* fatty acid biosynthesis in *E. faecalis*, and we observed less significant impacts on PGs and DGDGs when S613 was grown with *cis*-vaccenic acid.^25^ The observation that our data from S613 grown with an equimolar mixture of CV and OA better resemble the effects of OA-treated S613 suggests that OA more strongly represses *de novo* fatty acid biosynthesis than CV. However, the daptomycin-resistant strain, R712, appears to be slightly less susceptible to the repression of fatty acid biosynthesis by exogenous fatty acids while still achieving substantial incorporation of oleic acid.

**Figure 7:**
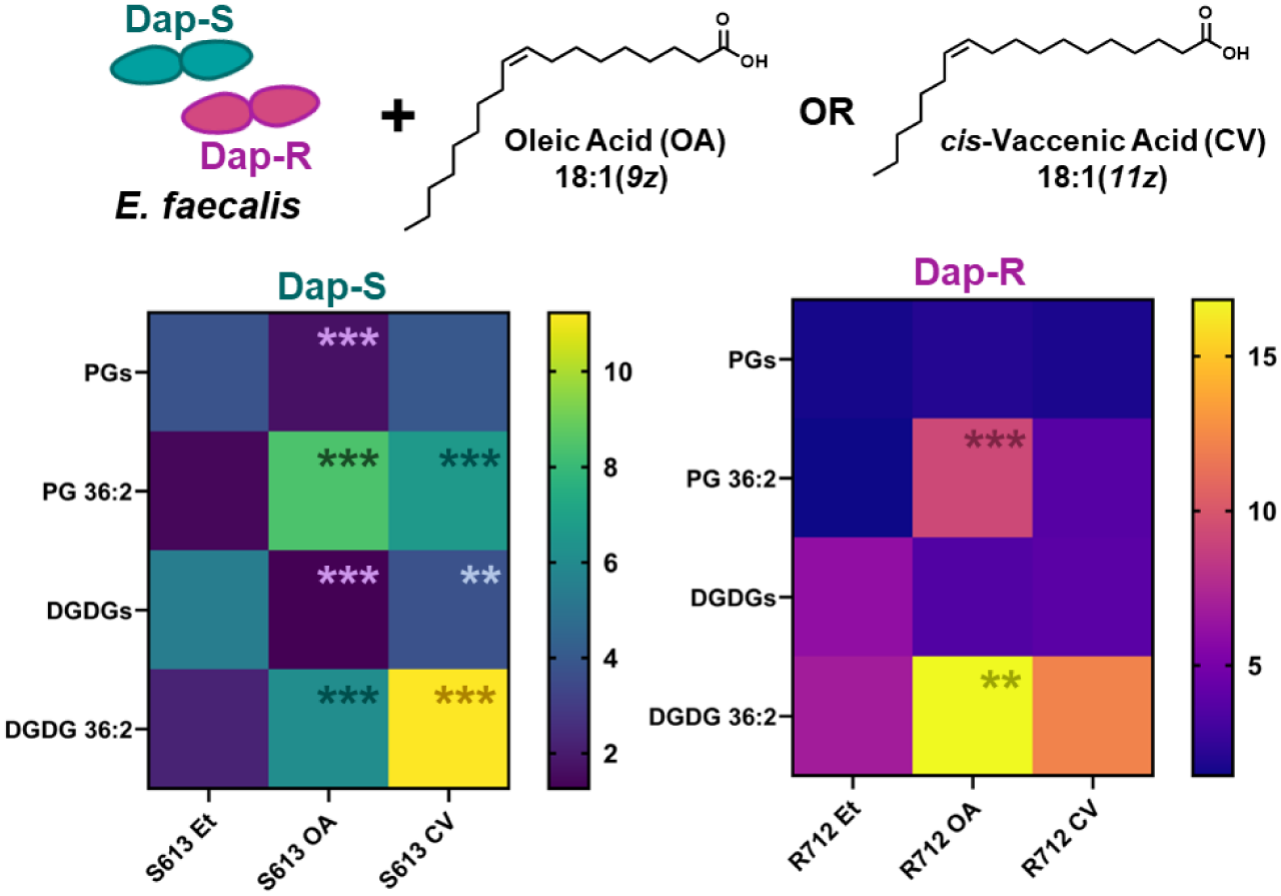
Summary of key changes in membrane lipids and Dap-S and Dap-R is provided with the FA 18:1 isomers, oleic acid, and *cis*-vaccenic acid. False discovery rate-adjusted multiple unpaired *t*-test *p*-values where *<0.05, **<0.01, ***<0.001.

The unexpected differences in the acyl tail compositions of PG 36:2 and DGDG 36:2, in which DGDG 36:2 contained almost exclusively OA when OA was provided alone or in an equimolar mixture with CV, raise questions about the biosynthetic pathways connecting these two lipid species. In *E. faecalis* and other Gram-positive bacteria, DGDGs and PGs are connected through the lipoteichoic acid (LTA) synthesis pathway. LTA is a glycerophosphate polymer that is anchored to the cell membrane by a DGDG lipid. Glycerophosphate units used to extend the LTA chain are derived from the headgroups of PGs in the inner membrane leaflet, creating a pool of DG that can be recycled into either PG or DGDG.^56,57^ The discrepancy between the acyl tail compositions of PG 36:2 and DGDG 36:2 when OA or OA+CV was provided suggests that a distinct pool of PG species is used for LTA and DGDG synthesis. Whether this distinction is based on structural preferences or spatial distributions cannot be discerned from these results.

Recent publications implicate the relationship between PGs, DGDGs, and LTA in oleic acid-mediated daptomycin tolerance. Scarpa de Mello et al. demonstrated that *Enterococcus faecium* is unable to establish daptomycin resistance in the absence of the gene, *lafB*, which completes the final step of DGDG synthesis.^20^ Furthermore, strains lacking *lafB* did not experience increased tolerance to daptomycin in the presence of oleic acid, whereas strains with *lafB* were 2-fold less sensitive. These results suggest that the direction of oleic acid into DGDGs is essential to oleic acid-mediated daptomycin tolerance. Short-term exposure of Dap-S *E. faecalis* to daptomycin was recently shown to induce a lipid composition that mirrors strains with genetically conferred daptomycin resistance, including reduced PGs and elevated DGDGs. Using pulse-chase radiolabeling, Colomer-Winter et al. demonstrated that PGs are immediately redirected to LTA and DGDG synthesis upon exposure to daptomycin through activation of LiaFSR.^57^ The displacement of anionic domains rich in CLs away from the division septum and toward peripheral regions, which is also induced by daptomycin activation of LiaFSR, has been proposed as another mechanism by which *E. faecalis* protects against damage by daptomycin.^18^

Based on the evidence presented above, we propose that Dap-S *E. faecalis* derives benefits from oleic acid through the net suppression of PGs, to levels similar to Dap-R strains, and increasing amounts of unsaturated PGs, DGDGs, and CLs that occur as newly synthesized lipids with incorporated oleic acid rather than fatty acids from *de novo* synthesis.^24,30,55^ Upon daptomycin exposure, the higher levels of CLs are more effective at displacing daptomycin from the division septum, while the pool of newly synthesized PGs can support DGDG and LTA synthesis.^18,58^ On the other hand, *E. faecalis* with daptomycin resistance derives no benefit from oleic acid due to the mutations in *liaF, cls,* and *gdpD* that achieve the same effect as oleic acid supplementation—reduced amounts of PG and enriched levels of FA 18:1-containing DGDGs.^19^ This study adds to the growing evidence that oleic acid derived from the host lipidome can induce daptomycin tolerance in *E. faecalis* by mirroring the membrane lipid alterations observed in genetically conferred daptomycin-resistant strains.

## Methods

### Materials and Chemicals

High-performance LC-grade solvents (water, acetonitrile, chloroform, methanol, isopropanol), ammonium acetate, and ammonium formate were obtained from Thermo Fisher Scientific. The fatty acid standards oleic (FA 18:1­(*9z*)) and *cis*-vaccenic (FA 18:1­(*11z*)) acid were purchased from Nu-Check and Cayman, respectively, and stored at −80 °C in ethanol. *Enterococcus faecalis* strain pairs S613 and R712 were obtained from the Werth Lab (UW) and were stored at −80 °C in a 1:1 sterile glycerol/water.

### Organisms and Growth

For all experiments, the *Enterococcus faecalis* strains, S613 (susceptible) and R712 (resistant), were streaked on nonsupplemented Brain Heart Infusion (BHI) agar plates and incubated at 37 °C overnight to midstationary phase. For all LC-MS experiments, 2 mL of 2 McFarland suspensions of S613 and R712 were cultured in triplicate in 18 mL of Brain Heart Infusion (BHI) broth with 100 μL of ethanol, 100 μL of 20 mM oleic acid (or *d*_9_-oleic acid), or 100 μL of 20 mM *cis*-vaccenic acid. In cases where the growths were supplemented with both isomers, 50 μL of each 20 mM stock were added so that the final total FA concentration was still 100 μM. The bacterial samples, performed in triplicate, were grown overnight in an incubator shaker at 37 °C and 200 rpm.

### Lipid Extraction

The centrifuged pellet was washed in 2 mL of sterile water, and the optical densities (OD_600 nm_) of each sample were measured from a 100 μL aliquot after diluting it with an additional 100 μL of water. Bacterial pellets were then extracted on ice using a modified version of the Bligh & Dyer method.^19,59^ The pellets were resuspended in 0.5 mL of HPLC-grade water, sonicated in an ice bath, and then 2 mL of chilled 1:2 chloroform/methanol was added. After vortexing periodically for 5 min, 0.5 mL of chilled chloroform and water were added to induce phase separation. The samples were briefly vortexed and centrifuged for 10 min. The lower organic layer was collected, dried under vacuum, and reconstituted in 1 mL of 1:1 chloroform/methanol.

### LC-MS

For all lipidomics experiments, besides the ozone-induced dissociation (OzID) data set, the lipid extracts and a quality control (QC) pooled mixture of 5 μL of each sample were analyzed using a Waters Acquity FTN I-Class Plus ultraperformance liquid chromatography (UPLC) system equipped with a Waters Acquity C18 (2.1 × 100 mm, 1.7 μm) column for reversed-phase liquid chromatography (RPLC) or a Waters CORTECS HILIC (2.1 × 100 mm, 1.6 μm) column for hydrophilic interaction chromatographic separation. The column temperatures were kept at 40 °C, and samples were maintained at 6 °C in the autosampler. The injection volume was 5 μL for all experiments and samples.

RPLC Mobile Phase A (MPA) consisted of 60:40 ACN/H_2_O with 10 mM ammonium formate, and Mobile Phase B (MPB) consisted of 88:10:2 IPA/ACN/H_2_O with 10 mM ammonium formate. A 30 min gradient at 0.3 mL/min was performed with the following conditions: 0–3 min, 30% MPB; 3–5 min, 30–50% MPB; 5–15 min, 50–90% MPB; 15–16 min, 90–99% MPB; 16–20 min, 99% MPB; 20–22 min, 99–30% MPB; 22–30 min, 30% MPB. Lipid extracts were prepared by drying 130 μL of lipid extract in a SpeedVac concentrator and reconstituting it in 65 μL of 60:40 ACN/H_2_O with 10 mM ammonium formate (RPLC MPA) to assess the PG and DGDG precursors, and a 4× concentration (150 to 60 μL) was used for targeted DGDGs to assess the lower-intensity fragments. HILIC MPA consisted of 95:5 ACN/H_2_O with 10 mM ammonium acetate, and MPB consisted of 50:50 ACN/H_2_O with 10 mM ammonium acetate. A 7 min gradient at a 0.5 mL/min flow rate was performed with the following conditions: 0–0.5 min, 100% MPB; 0.5–5 min, 100–60% MPB; 5–5.5 min, 60% MPB; 5.5–6 min, 60–100% MPB; 6–7 min, 100% MPB. Lipid extracts were prepared by drying 5 μL of extract, followed by reconstitution in 125 μL of 95:5 ACN/H_2_O with 10 mM ammonium acetate (HILIC MPA).

The Waters Acquity UPLC was connected to the electrospray ionization source of a Waters Synapt XS traveling-wave ion mobility mass spectrometer (TWIM-MS). Traveling wave separations were done with a wave velocity of 550 m/s and a wave height of 40 V, with a nitrogen flow of 90 mL/min. Mass calibration was performed with sodium formate over a 50–1200 *m*/*z* mass range. For all experiments, the samples were analyzed in the negative ionization mode. For HILIC, data were collected over 7 min with a collision energy ramp of 35–50 eV. For targeted MS/MS analyses, defined isolation windows were programmed based on the RPLC retention time and *m*/*z* value of the targeted precursor. The quadrupole isolation window, defined by the “LM Resolution” setting on the Synapt XS, was approximately 1.5 Da (LM Resolution 12). A collision energy ramp of 45–60 V was applied in the transfer region of the instrument. For the OzID data set, a detailed experimental section (SI Table S1) and a schematic of the instrument setup (SI Figure S1) can be found in the Supporting Information.

### Data Analysis

Progenesis QI (v3.0, Waters/Nonlinear Dynamics) was used to analyze the Waters.raw files with lock-mass correction and to align the samples with a pooled quality control sample for peak picking. All data were normalized using the “All Compounds” method within Progenesis, in which the feature abundances within each sample are normalized to the abundance of the pooled quality control sample and scaled to center the log10 ion ratio distributions to a normal distribution. Relative standard deviations of the peak areas from QCs measured throughout the RPLC run were less than 10%. Chromatographic peak areas of the lipid precursors were extracted from Progenesis and exported to Excel for further analysis. Lipid precursors were identified using the following adducts: PGs and LysylPGs with [M–H]^−^, DGDGs and MGDGs with [M + COOH]^−^ and [M + CH3COO]^−^, and CLs with [M–2H]^2–^. All lipid precursors were identified within 10 ppm mass accuracy relative to a custom version of LipidPioneer (see Supporting Information Tables S3 and S5 for ppm errors).^60^ To determine the relative distributions of fatty acyl tails within individual lipid species, the fatty acyl tail *m*/*z* values were used to create extracted ion chromatograms from the data-independent MS/MS function of the raw data (Function 2) in MassLynx. The peak areas were integrated from the extracted ion chromatograms, and the intensities of all acyl tail fragments were normalized to the intensity of the precursor from the MS1 data set.

### Growth Curve Assays

To evaluate the effect of fatty acids on the growth of *E. faecalis*, we conducted growth curve experiments with a microplate spectrophotometer (BioTek EPOCH 2, Gen 5, Agilent) in a 96-well culture plate (flat bottom, Fisher). For all assays, a bacterial inoculum of 0.5 McFarland was prepared in sterile 0.9%W/V sodium chloride. In a BSL-2 biosafety cabinet, 50 μL of the inoculum was added to 5 mL of BHI broth, and 25 μL of EtOH or 25 μL of 20 mM fatty acid was added to 5 mL of BHI broth for all assays performed. All experiments were performed in triplicate with five biological replicates per trial. For the growth curves without daptomycin, 50 μL of BHI broth was added to all wells, followed by 100 μL of BHI broth containing fatty acids or EtOH and 50 μL of S613 or R712 suspension. For the growth curves with daptomycin, S613 and R712 were prepared in calcium-adjusted BHI (30 mg/L) containing daptomycin (1, 4, or 64 μg/mL) and fatty acids (OA or CV) or an equivalent volume of ethanol. OD600 measurements were recorded at 30 min intervals over a 24 h period using a microplate spectrophotometer (BioTek EPOCH 2, Agilent). The plates were incubated at 37 °C during this time with orbital shaking for 30 s prior to each reading. Data were exported from Gen5 3.12 to Excel or GraphPad Prism (v9.02) for further analysis.

### Survival Assay

Calcium-adjusted BHI was inoculated with S613 (0.5 McFarland in 0.9% sterile saline) and grown to midexponential phase, at which point fatty acids were added to a final concentration of 100 μM, or an equivalent volume of ethanol was added for vehicle controls. The cultures were incubated for an additional 30 min, after which the bacteria were harvested by centrifugation. The pellets were washed and resuspended in 1× PBS, and then diluted 1:10 in fresh calcium-adjusted BHI containing 64 μg/mL of daptomycin. Cultures were incubated at 37 °C with shaking. Aliquots for CFU/mL enumeration were collected every 20 min over a 2 h period. Survival was calculated as the log ratio of CFU/mL relative to counts at 0 min. The assay was repeated with three biological replicates.

## Supplementary Material



## Data Availability

All LC-MS data sets from this publication are available in the MassIVE Repository under the identifier MSV000100134.

## Notes

Methods for ozone-induced dissociation; lipid identification information; additional class-level lipidomic results; additional species-level lipidomic results; additional data from mixed FA experiments; OzID fragmentation results; daptomycin growth curves (PDF)
