# Supplementary material for: Revealing the Fate of Isomeric Monounsaturated Fatty Acids in Enterococcus faecalis Membrane Lipids and Their Influence on Antimicrobial Susceptibility
Source: ACS Infect Dis. 2026 May 8;12(6):1980–91. doi: 10.1021/acsinfecdis.6c00092 (PMC13270503; doi:10.1021/acsinfecdis.6c00092)
Supplement: Supplementary file 1 [file id6c00092_si_001.pdf]

Supporting Information for

Revealing the fate of isomeric monounsaturated fatty acids in *Enterococcus faecalis* membrane lipids and their influence on antimicrobial susceptibility

Rebekah L. Casey [a], Annika L. Silverberg [b,c], Michael T. Marty [b,c], and Kelly M. Hines [a*]

[a] Department of Chemistry, University of Georgia, Athens, GA 30602, USA

[b] Department of Chemistry and Biochemistry, University of Arizona, Tucson, AZ 85721, USA

[c] Department of Chemistry, University of Texas at Austin, Austin, TX 78712, USA

*Address reprint requests to Kelly M. Hines: Department of Chemistry, University of Georgia, 302 East Campus Road, Athens, GA 30602; 706-542-1966; kelly.hines@uga.edu

ABSTRACT

Previous work suggests that Enterococcus faecalis uses exogenous oleic acid (FA 18:1(9z)) to increase its tolerance against the membrane targeting antimicrobial, daptomycin. However, the specific role of OA and the endogenous positional isomer, cis-vaccenic acid (FA 18:1(11z)), in both daptomycin-susceptible (Dap-S) and resistant (Dap-R) strains has not been fully explored. We performed lipidomics using hydrophilic interaction liquid chromatography and reversed phased liquid chromatography -ion mobility-mass spectrometry to identify alterations in lipid composition of Dap-S (S613) and Dap-R (R712) strain pairs of E. faecalis following OA or CV supplementation. Lipidomics showed that total PG intensity in Dap-S was only impacted by OA while total DGDG intensity was impacted by both OA and CV. However, total PG and DGDG intensities in Dap-R were not impacted by either FA. Both strains produced significantly more lipids containing two 18:1 acyl tails following OA supplementation, while CV had a similar effect on Dap-S only, indicating that Dap-S and Dap-R respond to OA and CV differently. Additionally, the preferences for OA vs CV alone and in mixtures were distinguished using ozonolysis and deuterium labeling techniques that can resolve C=C positional isomers. Growth and survival assays show that Dap-S responds differently to daptomycin when cultured with OA or CV. Together, these results reveal that E. faecalis strains with genetic determinants for daptomycin resistance respond differently, in membrane lipid composition and overall growth, to biologically relevant FA 18:1 isomers compared to strains that acquire daptomycin tolerance solely through exogenous fatty acid uptake.

TABLE OF CONTENTS Page

Ozone-Induced Dissociation Methods……………………………………………………...S-3

Supplemental Results & Figures……………………………………………………...S-6

1. Lipid Identifications……………………………………………………….S-6
2. Total Lipid Class Changes……………………………………………..............................S-8
3. Individual Lipid Changes……………………………………………………S-11
4. Fatty Acyl Tail Patterns……………………………………………………..S-16
5. Determining OA Incorporation……………………………………..............................S-29
    a. Mixture of OA and CV……………………………………………..S-29
    b. OzID Fragmentation…………………………..S-42
    c. Deuterium Labeling……………………………………………..S-61
6. Daptomycin Tolerance Assays……………………………………..............................S-69

OZONE-INDUCED DISSOCIATION EXPERIMENTAL

Sample Preparation. Each sample was resuspended in 1 mL 100% Optima MeOH and submitted for injection in the original MeOH resuspension.

Liquid Chromatography. Ultrahigh performance liquid chromatography (UPLC) was performed using a Waters 2D Acquity I-Class system, with the autosampler set to 10 °C and the column compartment set to 60 °C. For each sample, 10 µL was injected. The lipids were first trapped on a Waters XBridge C8 Direct Connect HP (10 µM, 2.1 x 30 mm) column, then separated and eluted using a Waters ACQUITY Premier CSH C18 (1.7 µm, 2.1 x 100 mm) column. Mobile Phase A consisted of 60/40 ACN/H_2O with 10 mM ammonium acetate and Mobile Phase B consisted of 90/10 IPA/ACN with 10 mM ammonium acetate. The LC gradient is detailed in **SI Table S1**. After the separation, the lipids entered a Waters Synapt XS for OzID.

Ozone Parameters. Ultra-high purity (UHP) oxygen gas was plumbed into the ozone generator to produce ozone. The ozone flow was set around 6% Wt%Air, with occasional drift ranging from 5-7% ozone Wt%Air. Ozone was constantly pumped into the ion mobility cell for the duration of all runs.

MS Parameters. A TG-40 Ozone generator equipped with an ambient ozone monitor (Ozone Solutions) was plumbed into the ion mobility cell of the Synapt XS (**SI Figure S1**). The phosphatidylglycerol (PG) lipids were ionized using negative ESI polarity. The capillary and sampling cone voltages were set to -1.5 kV and -40 V respectively. The source temperature was 120 °C, and the desolvation temperature was 450 °C. The cone, desolvation, and nebulizer gas flows were 40.0, 700.0, and 6.0 L/Hr, respectively. The diglucosyl diacylglycerol (DGDG) lipids were ionized using positive ESI polarity. The capillary and sampling cone voltages were set to +2.0 kV and +35.0 V respectively. The source temperature was 120 °C, and the desolvation temperature was 450 °C. The cone, desolvation, and nebulizer gas flows were 50.0, 700.0, and 6.0 L/Hr respectively. The ion mobility settings were the same for all samples. The manual transfer collision energy was 12.0 V, and the trap, helium, and IMS gas flows were 3.0, 180.0, and 10.0 mL/min respectively. The IMS wave velocity was 2320 m/s, and the IMS wave height was 6.0 V. Each precursor of interest (PG and DGDGs 32:1, 34:1, 35:1, 34:2, 32:2, 36:2,

and 37:2) was isolated for fragmentation using the quadrupole and exposed to ozone in the ion mobility cell, followed by additional collision induced dissociation in the transfer regions.

Data Collection and Analysis. In each MSMS spectrum, the extracted ion chromatograms (EICs) were taken for each corresponding OzID fragment. The peaks were integrated, their areas normalized to 1, and used to calculate the ratios of *9z*, *11z*, and *9z/11z* isomers within each sample. Certain PG lipids, specifically 36:2, 34:2, and 32:2, showed additional fragmentation patterns between 500-700 *m/z*. We concluded that these additional peaks correspond to PG lipids that have both *9z* and *11z* double bond positioning in their tails. Interestingly, this was not observed in any DGDG spectra.

Table S1. Summary of LC gradients used in the collection of chromatographic peaks of samples subjected to OzID.

**Alpha Pump**				
**Time (min)**	**Flow (mL/min)**	**%A**	**%B**	**Curve**
Initial	1.000	70.0	30.0	Initial
0.50	1.000	60.0	40.0	6
1.01	1.000	1.0	99.0	6
17.00	1.000	1.0	99.0	11
20.00	1.000	70.0	30.0	11
**Beta Pump**				
**Time (min)**	**Flow (mL/min)**	**%A**	**%B**	**Curve**
Initial	0.25	60.0	40.0	Initial
0.50	0.25	60.0	40.0	6
1.50	0.25	50.0	50.0	6
2.50	0.25	40.0	60.0	6
2.90	0.25	34.0	66.0	6
4.50	0.25	32.3	67.7	6
4.51	0.25	32.2	67.8	11
12.00	0.25	30.0	70.0	6
13.00	0.25	10.0	90.0	6
15.00	0.25	10.0	90.0	6
15.50	0.25	1.0	99.0	6
17.50	0.25	1.0	99.0	11
18.00	0.25	60.0	40.0	11

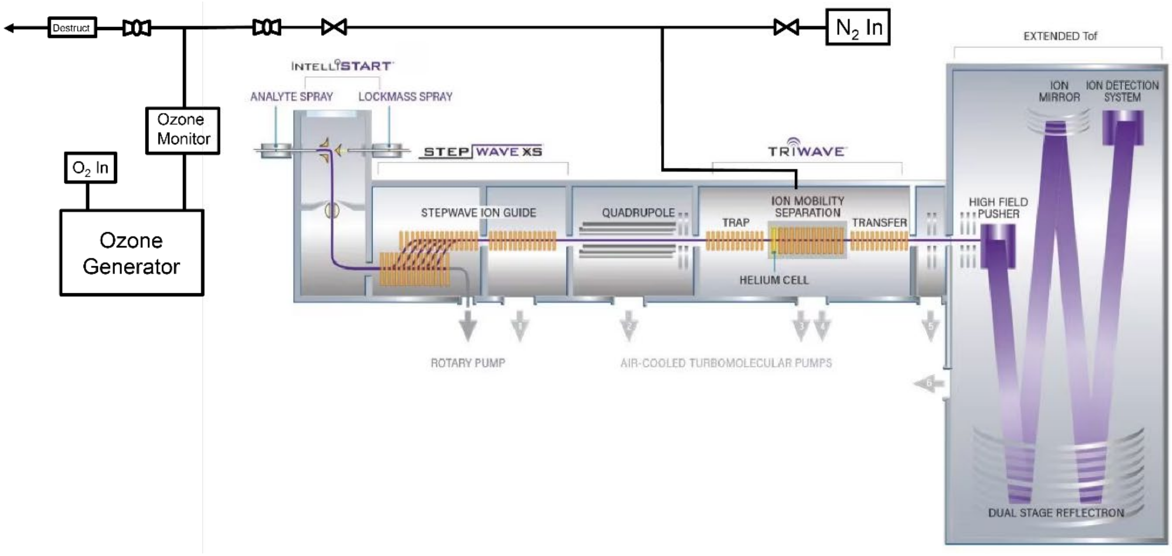

Figure S1: Schematic of the ozone generator plumbed into the Waters Synapt XS.

SUPPLEMENTAL RESULTS & FIGURES

1. Lipid Identifications

Table S2. IDs and mass errors (ppm) of PGs, DGDGs, and MGDGs from RPLC-IM-MS.

Compound	Adduct	RT	Observed *m/z*	Exact *m/z*	Mass Error
**PG 32:1**	$[M-H]^-$	8.97	719.4867	719.4868	-0.2
**PG 34:1**	$[M-H]^-$	9.93	747.5166	747.5181	-2.1
**PG 35:1**	$[M-H]^-$	10.52	761.5328	761.5338	-1.3
**PG 32:2**	$[M-H]^-$	8.15	717.4706	717.4712	-0.9
**PG 34:2**	$[M-H]^-$	9.06	745.5010	745.5025	-2.0
**PG 36:2**	$[M-H]^-$	10.00	773.5325	773.5338	-1.7
**PG 37:2**	$[M-H]^-$	10.60	787.5484	787.5494	-1.2
**DGDG 32:1**	$[M+HCOO]^-$	10.06	935.5940	935.5949	-0.9
**DGDG 34:1**	$[M+HCOO]^-$	10.95	963.6253	963.6262	-0.9
**DGDG 35:1**	$[M+HCOO]^-$	11.56	977.6419	977.6418	0.1
**DGDG 32:2**	$[M+HCOO]^-$	9.25	933.5786	933.5792	-0.7
**DGDG 34:2**	$[M+HCOO]^-$	10.13	961.6093	961.6105	-1.3
**DGDG 36:2**	$[M+HCOO]^-$	11.00	989.6415	989.6418	-0.3
**DGDG 37:2**	$[M+HCOO]^-$	11.61	1003.6569	1003.6575	-0.6
**MGDG 32:1**	$[M+HCOO]^-$	10.69	773.5413	773.5420	-0.9
**MGDG 34:1**	$[M+HCOO]^-$	11.52	801.5730	801.5733	-0.5
**MGDG 35:1**	$[M+HCOO]^-$	12.12	815.5885	815.5890	-0.5
**MGDG 32:2**	$[M+HCOO]^-$	9.81	771.5252	771.5264	-1.5
**MGDG 34:2**	$[M+HCOO]^-$	10.69	799.5570	799.5577	-0.8
**MGDG 36:2**	$[M+HCOO]^-$	11.61	827.5885	827.5890	-0.6
**MGDG 37:2**	$[M+HCOO]^-$	12.14	841.6039	841.6046	-0.8

Table S3. IDs and mass errors (ppm) of FA 18:1 (free and fragmented, eluting at the PG and DGDG retention times), CLs, and LysylPGs detected in HILIC-IM-MS.

Compound	Adduct	RT	Observed *m/z*	Exact *m/z*	Mass Error
**FFA 18:1**	$[M-H]^-$	0.74	281.2479	281.2486	-2.5
**10-HSA**	$[M-H]^-$	0.93	299.2602	299.2591	3.7
**FA 18:1**	$[M-H]^-$	1.19	281.2480	281.2486	-2.1
**FA 18:1**	$[M-H]^-$	1.96	281.2480	281.2486	-2.1
**PG 32:1**	$[M-H]^-$	1.98	719.4876	719.4868	1.1
**PG 34:1**	$[M-H]^-$	1.98	747.5191	747.5181	1.3
**PG 35:1**	$[M-H]^-$	1.95	761.5330	761.5338	-1.0
**PG 32:2**	$[M-H]^-$	1.98	717.4724	717.4712	1.6
**PG 34:2**	$[M-H]^-$	1.95	745.5043	745.5025	2.4
**PG 36:2**	$[M-H]^-$	1.95	773.5351	773.5338	1.7
**PG 37:2**	$[M-H]^-$	1.95	787.5502	787.5494	1.0
**DGDG 32:1**	$[M+CH3COO]^-$	1.21	949.6087	949.6105	-1.85
**DGDG 34:1**	$[M+CH3COO]^-$	1.21	977.6397	977.6418	-2.16
**DGDG 34:2**	$[M+CH3COO]^-$	1.17	975.6231	975.6262	-3.18
**DGDG 36:2**	$[M+CH3COO]^-$	1.17	1003.6557	1003.6575	-1.80
**CL 64:3**	$[M-2H]^{2-}$	2.79	672.4479	672.4553	-10.9
**CL 66:3**	$[M-2H]^{2-}$	2.84	686.4665	686.4710	-6.5
**CL 68:3**	$[M-2H]^{2-}$	2.88	700.4842	700.4866	-3.5
**CL 72:4**	$[M-2H]^{2-}$	2.84	727.5101	727.5101	0.0
**LysylPG 30:0**	[M-H]-	4.80	821.5636	821.5661	-3.0
**LysylPG 30:1**	[M-H]-	4.77	819.5485	819.5505	-2.4
**LysylPG 32:1**	[M-H]-	4.74	847.5808	847.5818	-1.1
**LysylPG 34:1**	[M-H]-	4.74	875.6126	875.6131	-0.6
**LysylPG 35:1**	[M-H]-	4.70	889.6283	889.6287	-0.5
**LysylPG 36:2**	[M-H]-	4.65	901.6269	901.6287	-2.0

2. Total Lipid Class Changes

Table S4. Summary table of the false-discovery rate adjusted *p*-values from multiple unpaired *t*-tests performed for the total PGs and DGDGs and fragmented or free FA 18:1 abundances from the RPLC/ HILIC-IM-MS data sets displayed in *Figure 1.*

Condition 1	Condition 2	PGs	PG FA 18:1	DGDGs	DGDG FA 18:1	FFA 18:1
**S613 Et**	**S613 OA**	<0.0001	0.00259	<0.0001	<0.0001	<0.0001
**S613 Et**	**S613 CV**	0.32	<0.0001	0.0087	0.013	<0.0001
**R712 Et**	**R712 OA**	0.46	<0.0001	0.16	0.0095	<0.0001
**R712 Et**	**R712 CV**	0.89	<0.0001	0.31	0.083	<0.0001

Table S5. Summary table of the log_2 fold changes of the FA conditions compared to EtOH controls calculated from the total lipid class and fragmented and free FA 18:1 abundances from the RPLC/HILIC-IM-MS data set displayed in *Figure 1.*

**LOG_2 FOLD CHANGE**						
**Condition 1**	**Condition 2**	**PGs**	**PG FA 18:1**	**DGDGs**	**DGDG FA 18:1**	**FFA 18:1**
**S613 Et**	**S613 OA**	-1.16	0.42	-2.10	-2.02	1.36
**S613 Et**	**S613 CV**	0.07^{ns}	0.97	-0.53	-0.14^{ns}	1.02
**R712 Et**	**R712 OA**	0.28^{ns}	1.26	-0.78^{ns}	-0.48	1.16
**R712 Et**	**R712 CV**	0.09^{ns}	0.86	-0.64^{ns}	0.09^{ns}	0.79

<u>Note:</u> the superscripted "ns" are the log_2 fold changes that are not statistically significant based on *SI Table S2*

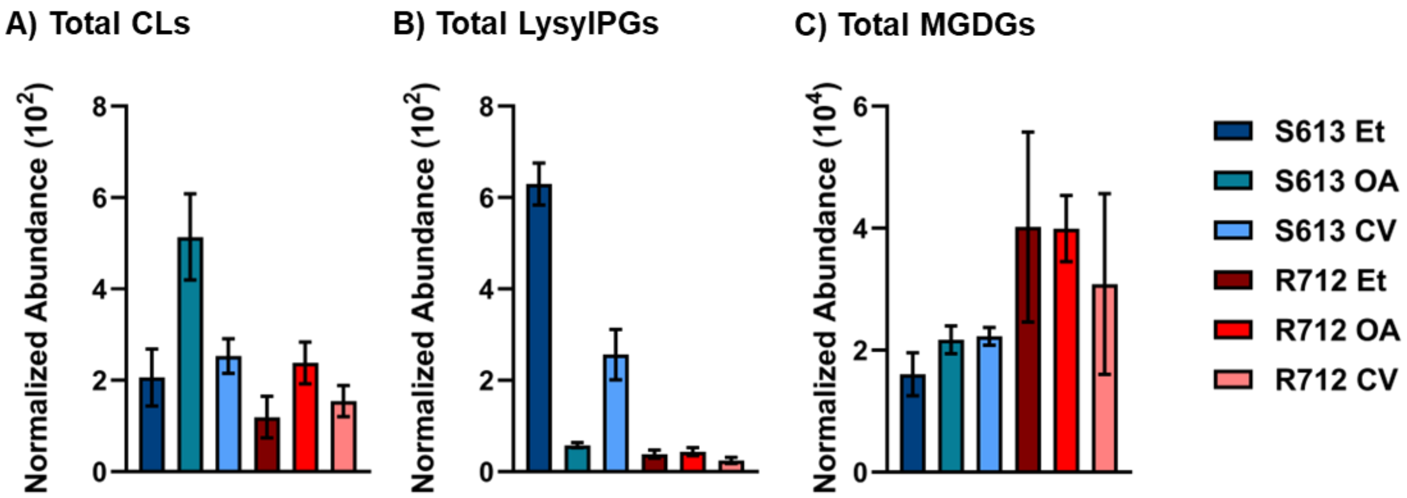

Figure S2. Impact of oleic and *cis*-vaccenic acid on A) total CLs and B) total LysylPGs, and C) total MGDGs. *P*-values and fold-changes provided in *SI Table 6*.

Table S6. Summary table of the false-discovery rate adjusted *p*-values from multiple unpaired *t*-tests and $\log_2$ fold changes for the CL, LysylPG, and MGDG total lipid class abundances from the data set displayed in *SI Figure S2.*

P-VALUES				
**Condition 1**	**S613 Et**	**S613 Et**	**R712 Et**	**R712 Et**
**Condition 2**	**S613 OA**	**S613 CV**	**R712 OA**	**R712 CV**
**CL**	0.0095	0.17	0.067	0.35
**LysylPG**	<0.0001	0.00087	0.56	0.21
**MGDG**	0.028	0.050	>0.99	0.78
**LOG2 FOLD-CHANGE**				
**Condition 1**	**S613 Et**	**S613 Et**	**R712 Et**	**R712 Et**
**Condition 2**	**S613 OA**	**S613 CV**	**R712 OA**	**R712 CV**
**CL**	1.32	0.30	1.00	0.37
**LysylPG**	-3.43	-1.30	0.18	-0.65
**MGDG**	0.43	0.47	-0.01	-0.38

3. Individual Lipid Changes

Table S7. Summary table of the false-discovery rate adjusted *p*-values from multiple unpaired *t*-tests performed for the PG and DGDG precursor abundances from the RPLC-IM-MS data set displayed in *Figures 2 and 3*.

**Condition 1**	**S613 Et**	**S613 Et**	**R712 Et**	**R712 Et**
**Condition 2**	**S613 OA**	**S613 CV**	**R712 OA**	**R712 CV**
**PG 32:1**	<0.0001	0.0075	0.038	0.99
**PG 34:1**	<0.0001	0.028	0.086	>0.99
**PG 35:1**	<0.0001	0.00051	0.0094	0.70
**PG 32:2**	<0.0001	0.00051	0.038	0.75
**PG 34:2**	<0.0001	0.18	0.056	0.99
**PG 36:2**	<0.0001	0.00033	<0.0001	0.70
**PG 37:2**	<0.0001	<0.0001	0.00026	0.75
**DGDG 32:1**	<0.0001	<0.0001	0.0060	0.058
**DGDG 34:1**	<0.0001	0.00010	0.0060	0.058
**DGDG 35:1**	0.00044	0.014	0.0067	0.058
**DGDG 32:2**	<0.0001	<0.0001	0.012	0.058
**DGDG 34:2**	<0.0001	<0.0001	0.0067	0.058
**DGDG 36:2**	0.00016	<0.0001	0.0060	0.11
**DGDG 37:2**	0.00022	0.00066	0.015	0.40

Table S8. Summary table of the log_2 fold changes of the FA conditions compared to the EtOH controls calculated from the PG and DGDG precursor abundances from the RPLC-IM-MS data set displayed in *Figures 2 and 3.*

**LOG_2 FOLD CHANGE**				
**Condition 1**	**S613 Et**	**S613 Et**	**R712 Et**	**R712 Et**
**Condition 2**	**S613 OA**	**S613 CV**	**R712 OA**	**R712 CV**
**PG 32:1**	-3.19	-0.24	-1.01	-0.11 ^{ns}
**PG 34:1**	-1.76	0.22	-0.50 ^{ns}	-0.01 ^{ns}
**PG 35:1**	-3.12	-0.50	-1.63	-0.55 ^{ns}
**PG 32:2**	-3.71	-0.61	-1.10	-0.37 ^{ns}
**PG 34:2**	-2.71	0.10 ^{ns}	-1.24 ^{ns}	-0.14 ^{ns}
**PG 36:2**	2.58	2.23	2.68	1.37 ^{ns}
**PG 37:2**	1.92	1.25	2.86	0.76 ^{ns}
**DGDG 32:1**	-5.61	-1.24	-2.52	-0.99 ^{ns}
**DGDG 34:1**	-4.86	-1.33	-2.57	-1.02 ^{ns}
**DGDG 35:1**	-4.76	-0.84	-3.02	-1.29 ^{ns}
**DGDG 32:2**	-6.52	-1.59	-3.16	-1.43 ^{ns}
**DGDG 34:2**	-5.57	-1.69	-3.06	-1.32 ^{ns}
**DGDG 36:2**	1.48	2.36	1.30	0.83 ^{ns}
**DGDG 37:2**	2.41	2.08	2.33	0.43 ^{ns}

Note: the superscripted "ns" are the log_2 fold changes that are not statistically significant based on *p*-values displayed in *SI Table S7*

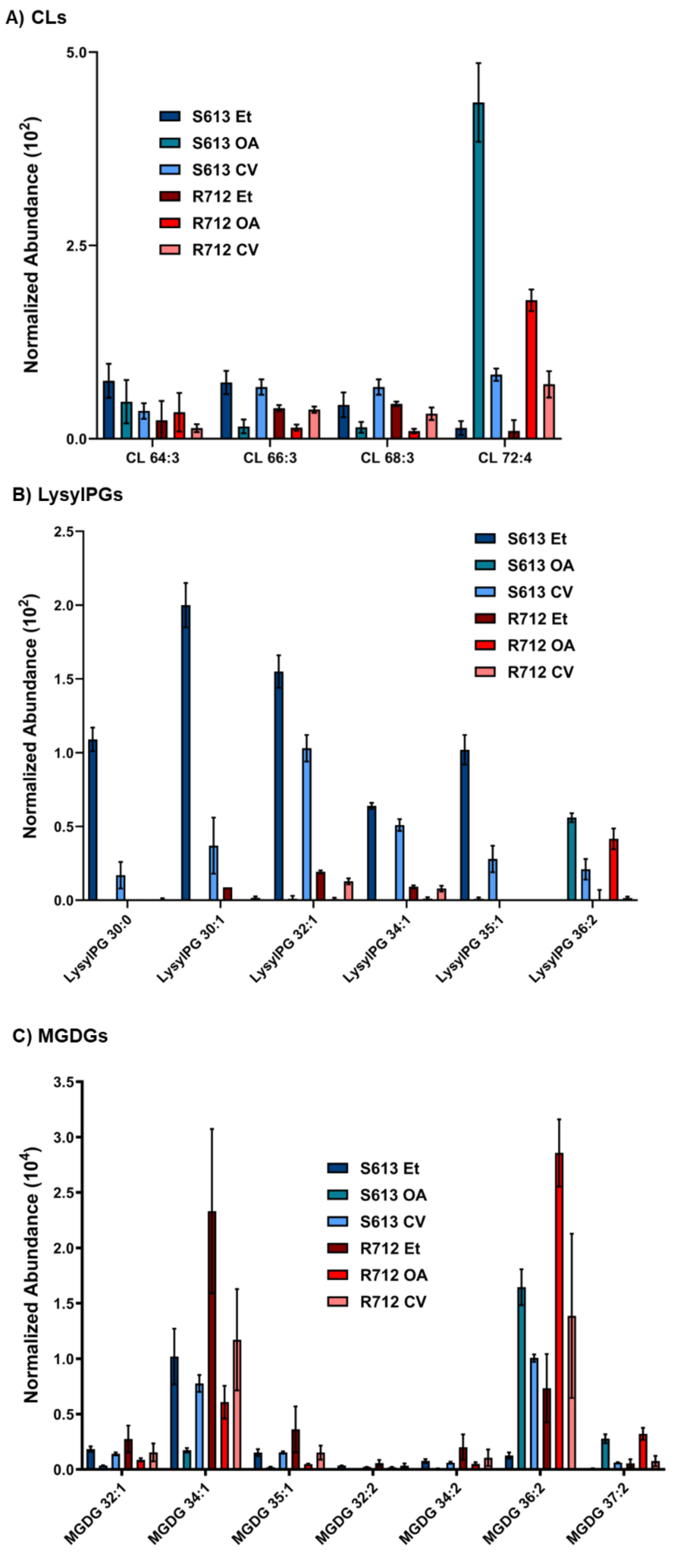

Figure S3. Impact of oleic and *cis*-vaccenic acid on A) individual CLs, B) individual LysylPGs, and C) individual MGDGs lipid species identified in *SI Table S5. P*-values and fold-changes provided in *SI Tables S9 and S10.*

Table S9. Summary table of the false-discovery rate adjusted *p*-values from multiple unpaired *t*-tests performed for the CL, LysylPG, and MGDG precursor abundances from the data displayed in SI *Figures S3*.

**Condition 1**	**S613 Et**	**S613 Et**	**R712 Et**	**R712 Et**
**Condition 2**	**S613 OA**	**S613 CV**	**R712 OA**	**R712 CV**
**CL 64:3**	0.14	0.082	0.17	0.45
**CL 66:3**	0.0051	0.50	0.00053	0.45
**CL 68:3**	0.032	0.11	<0.0001	0.098
**CL 72:4**	0.00031	0.0019	<0.0001	0.029
**LysylPG 30:0**	<0.0001	0.00097	N/A	0.17
**LysylPG 30:1**	<0.0001	0.00097	N/A	0.00084
**LysylPG 32:1**	<0.0001	0.0050	<0.0001	0.015
**LysylPG 34:1**	<0.0001	0.0073	0.00093	0.32
**LysylPG 35:1**	<0.0001	0.0014	N/A	N/A
**LysylPG 36:2**	<0.0001	0.0073	0.0020	0.46
**MGDG 32:1**	0.00081	0.054	0.049	0.36
**MGDG 34:1**	0.0046	0.15	0.024	0.36
**MGDG 35:1**	0.0024	0.56	0.049	0.36
**MGDG 32:2**	<0.0001	0.00036	0.050	0.36
**MGDG 34:2**	0.0024	0.15	0.052	0.36
**MGDG 36:2**	0.00033	<0.0001	0.0044	0.36
**MGDG 37:2**	0.00075	<0.0001	0.0044	0.60

Note: "N/A" indicates the absence of the lipid species in both of the conditions being compared

Table S10. Summary table of the log_2 fold changes of the FA conditions compared to the EtOH controls calculated from the CL, LysylPG, and MGDG precursor abundances from the data set displayed in SI *Figure S3*.

**LOG_2 FOLD CHANGE**				
**Condition 1**	**S613 Et**	**S613 Et**	**R712 Et**	**R712 Et**
**Condition 2**	**S613 OA**	**S613 CV**	**R712 OA**	**R712 CV**
**CL 64:3**	-0.63^{ns}	-1.03^{ns}	0.51^{ns}	-0.80^{ns}
**CL 66:3**	-2.22	-0.11^{ns}	-1.45	-0.08^{ns}
**CL 68:3**	-1.52^{ns}	0.59^{ns}	-2.17	-0.47^{ns}
**CL 72:4**	4.93	2.54	4.12	2.78
**LysylPG 30:0**	N/A	-2.69	N/A	-0.65^{ns}
**LysylPG 30:1**	N/A	-2.43	N/A	-2.45^{ns}
**LysylPG 32:1**	-6.75	-0.58	-4.63^{ns}	-0.58^{ns}
**LysylPG 34:1**	N/A	-0.33	-3.07	-0.20^{ns}
**LysylPG 35:1**	-7.39	-1.88	N/A	N/A
**LysylPG 36:2**	N/A	N/A	N/A	N/A
**MGDG 32:1**	-2.34	-0.38^{ns}	-1.64^{ns}	-0.83^{ns}
**MGDG 34:1**	-2.55	-0.39^{ns}	-1.94	-0.99^{ns}
**MGDG 35:1**	-2.80	0.02^{ns}	-2.92^{ns}	-1.24^{ns}
**MGDG 32:2**	-2.91	-0.63	-1.56^{ns}	-0.76^{ns}
**MGDG 34:2**	-3.09	-0.32^{ns}	-1.97^{ns}	-0.93^{ns}
**MGDG 36:2**	3.70	2.99	1.96	0.92^{ns}
**MGDG 37:2**	5.00	2.84	2.54	0.46^{ns}

Note: the superscripted "ns" are the log_2 fold changes that are not statistically significant based on *SI Table S9* and the "N/A" indicates the absence of the lipid species in one or both of the conditions being compared

4. Fatty Acyl Tail Patterns

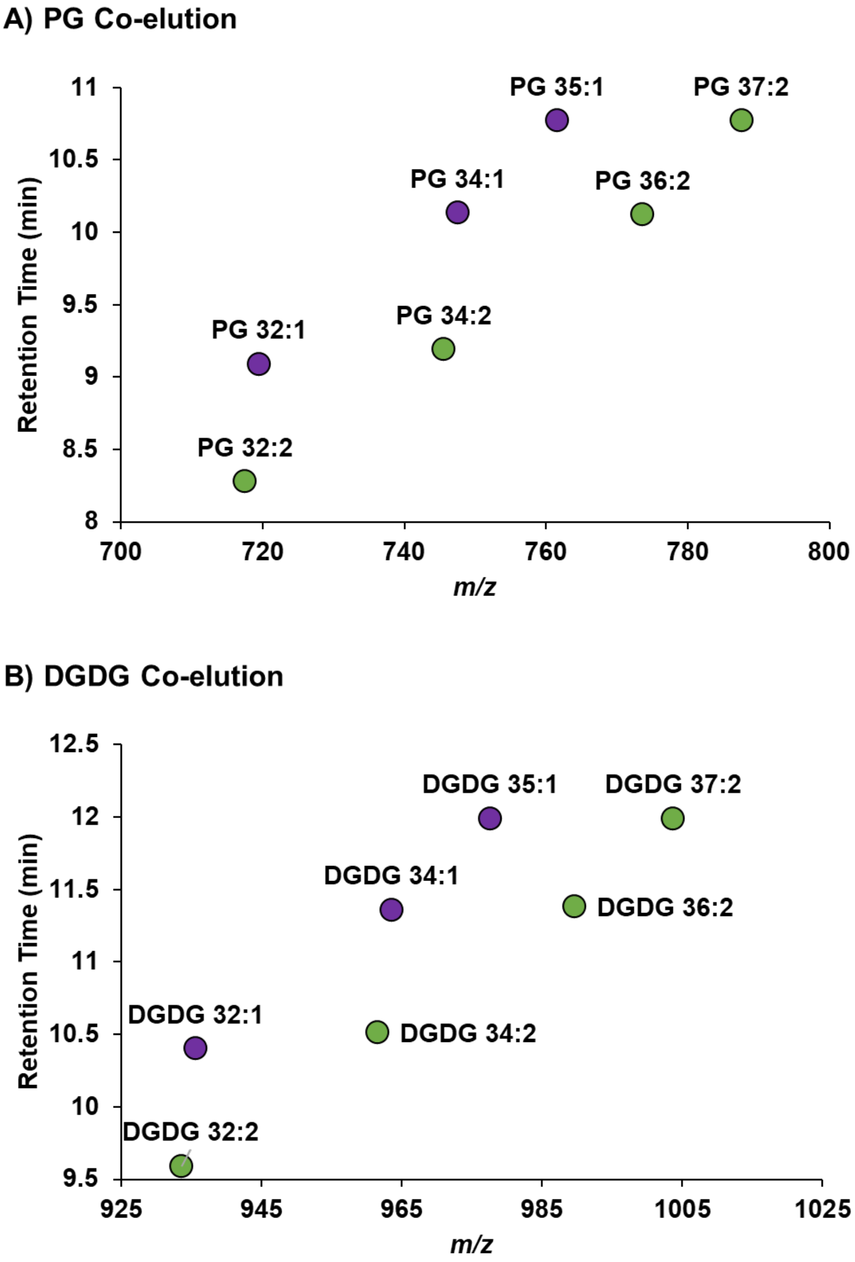

Figure S4. Co-elution pattern observed in RPLC-IM-MS between the of mono-unsaturated and doubly-unsaturated A) PGs and B) DGDGs.

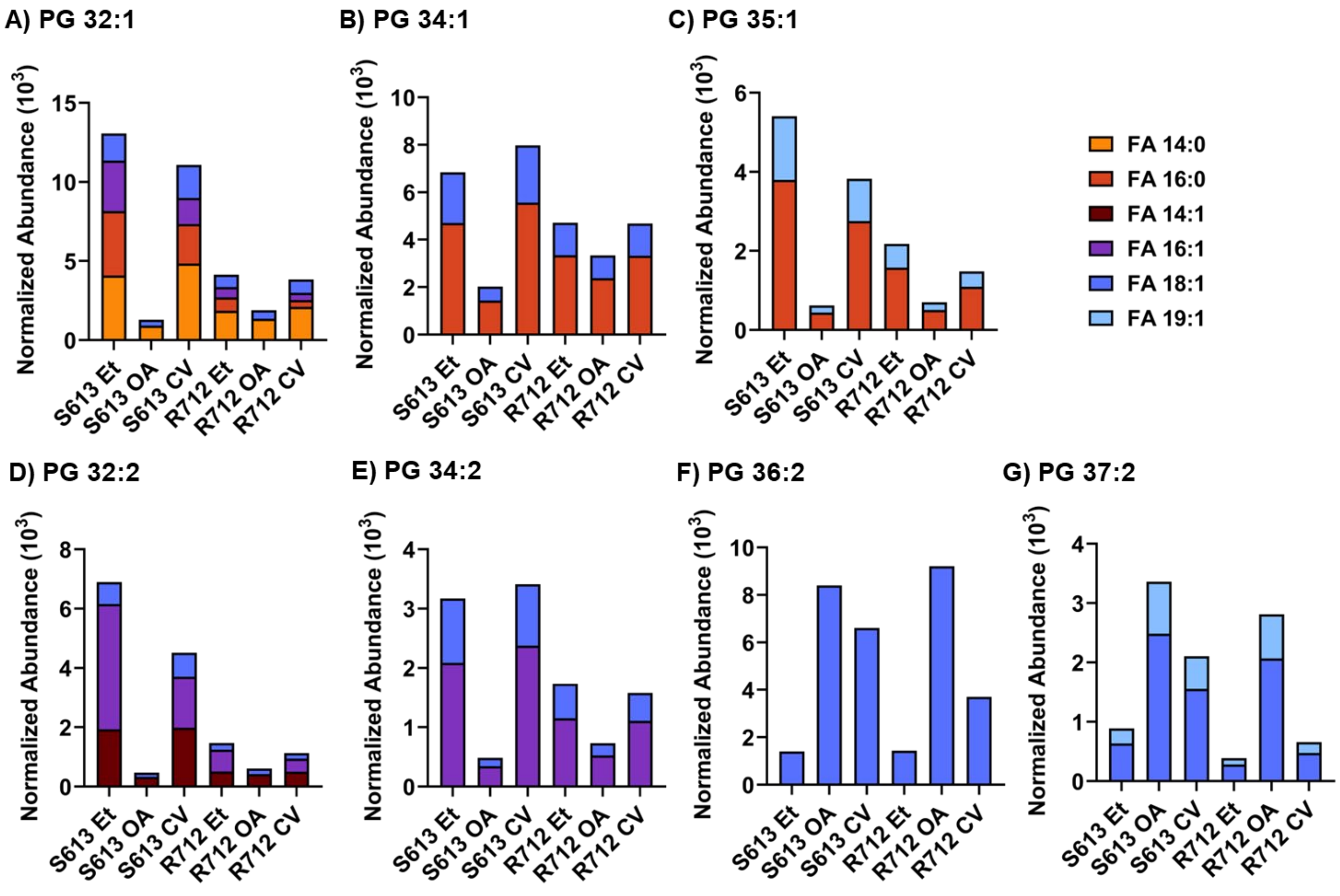

Figure S5. Targeted RPLC-IM-MS/MS of FA distributions of PG precursors.

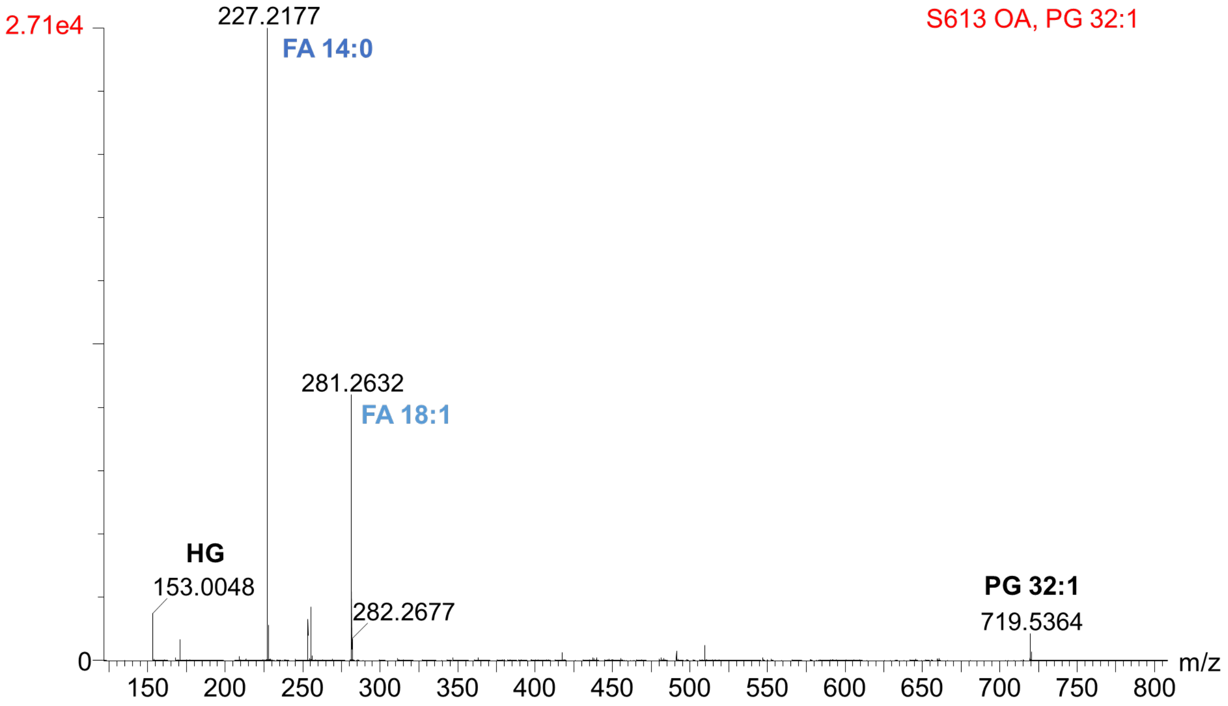

Figure S6a. MSMS fragmentation of PG 32:1 in condition S613 OA.

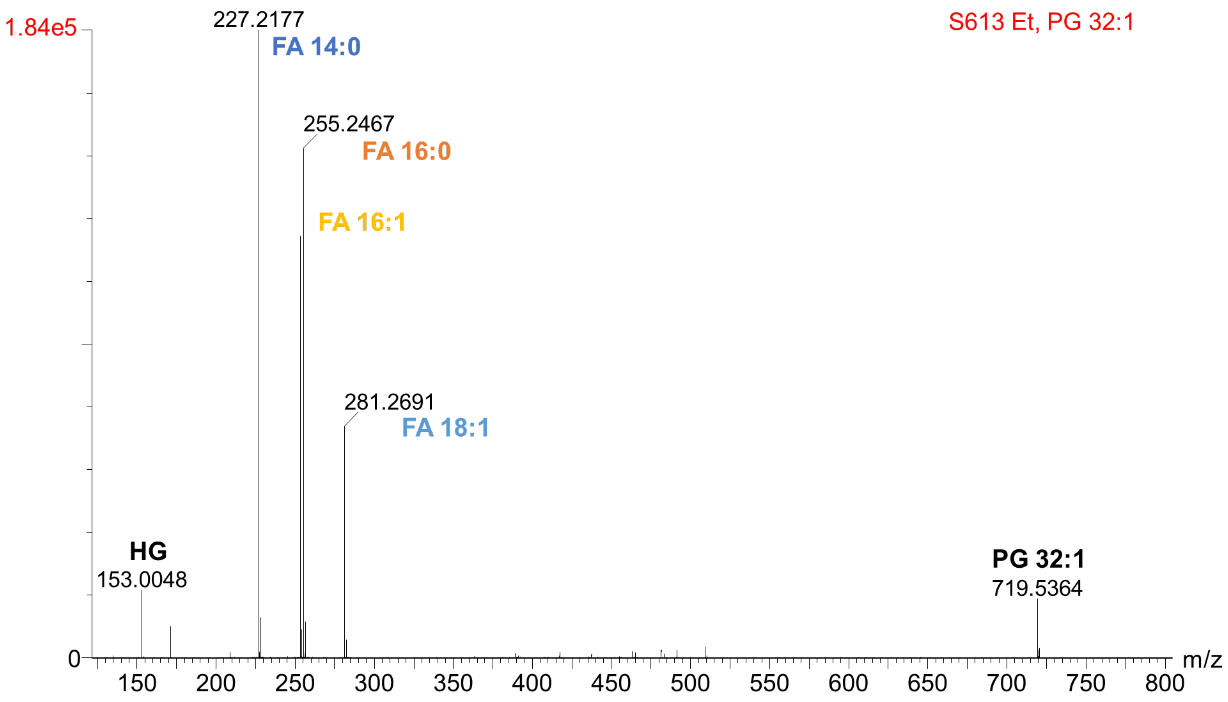

Figure S6b. MSMS fragmentation of PG 32:1 in condition S613 Et.

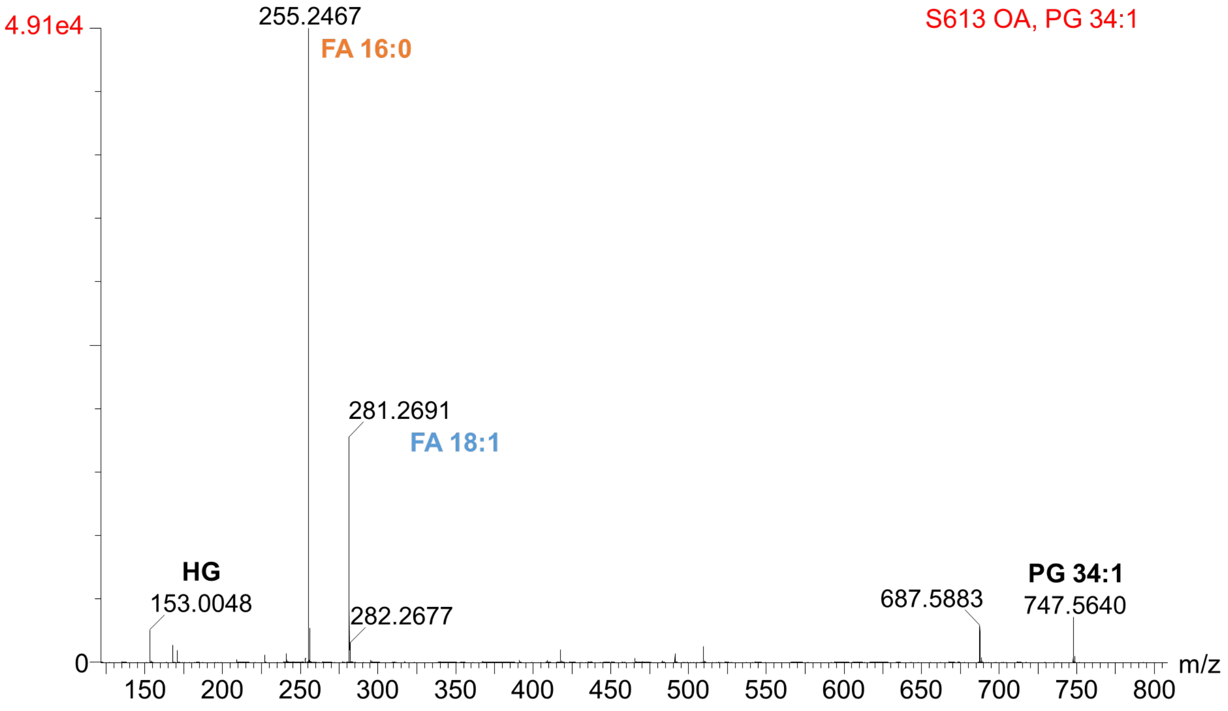

Figure S6c. MSMS fragmentation of PG 34:1 in condition S613 OA.

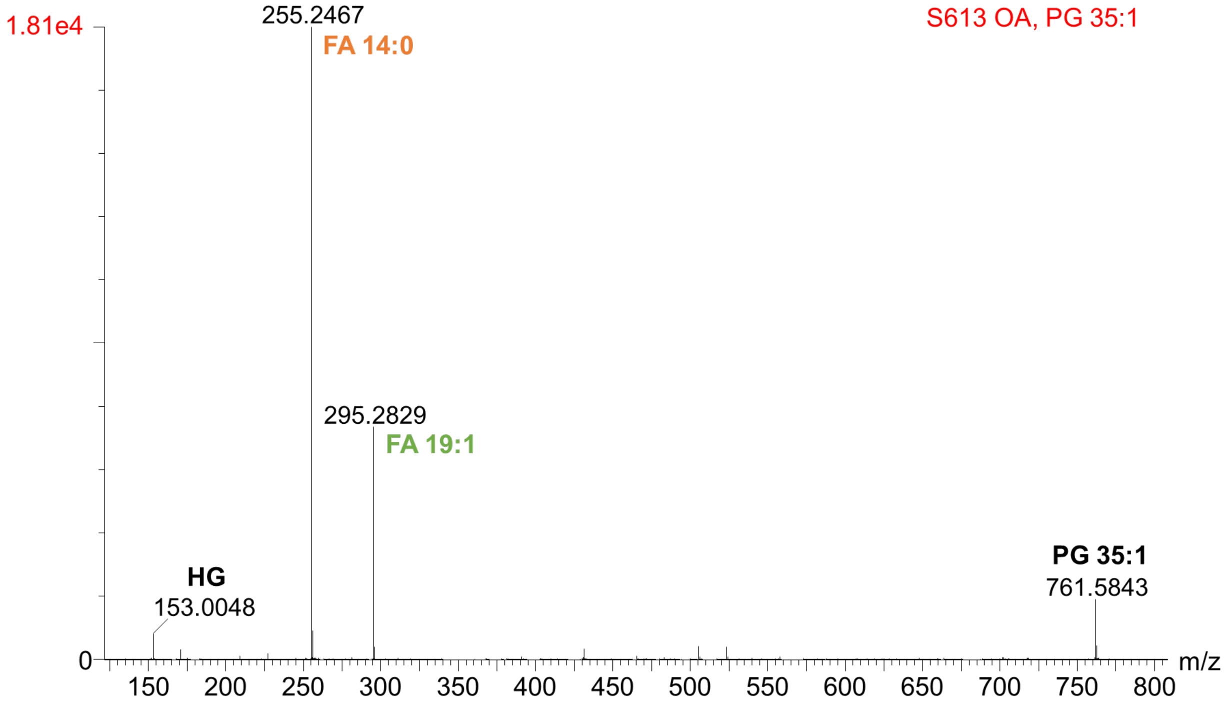

Figure S6d. MSMS fragmentation of PG 35:1 in condition S613 OA.

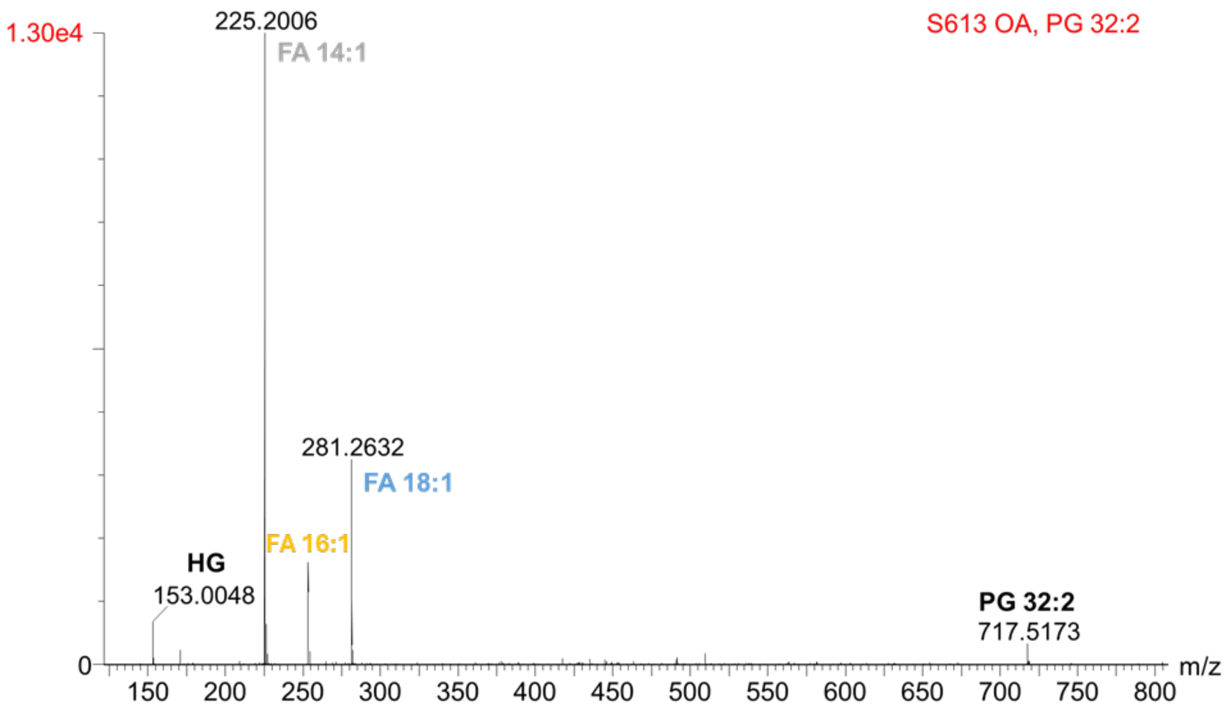

Figure S6e. MSMS fragmentation of PG 32:2 in condition S613 OA.

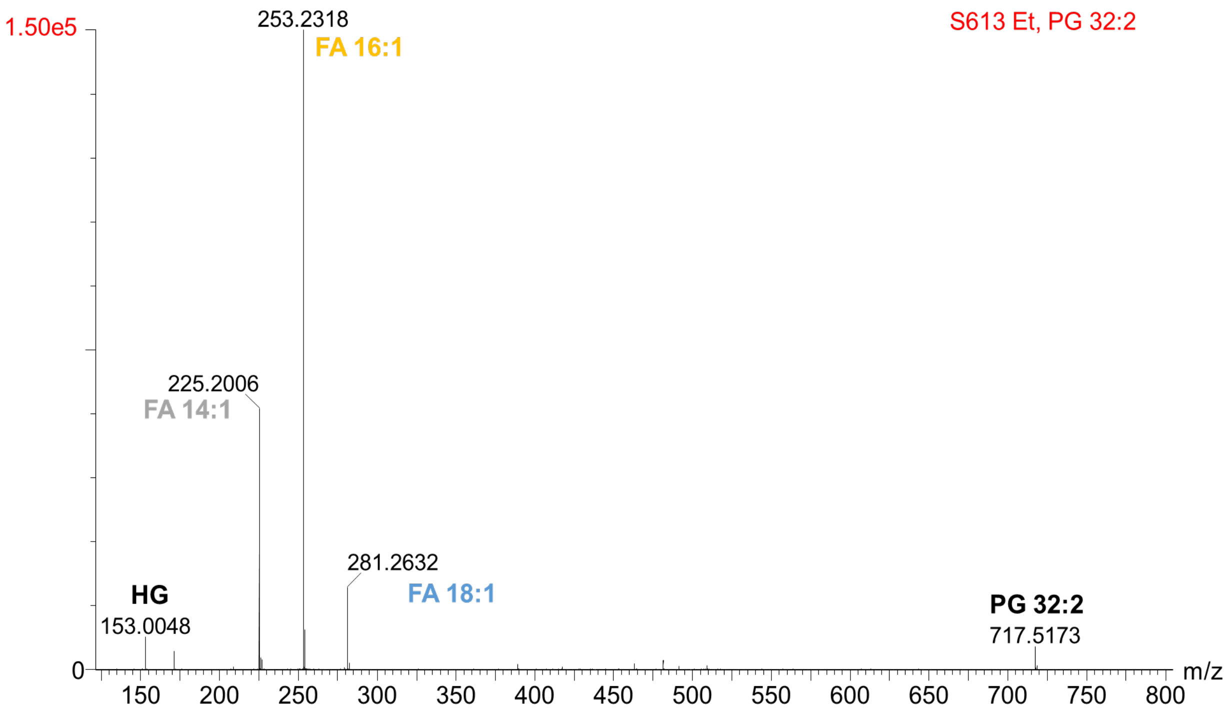

Figure S6f. MSMS fragmentation of PG 32:2 in condition S613 Et.

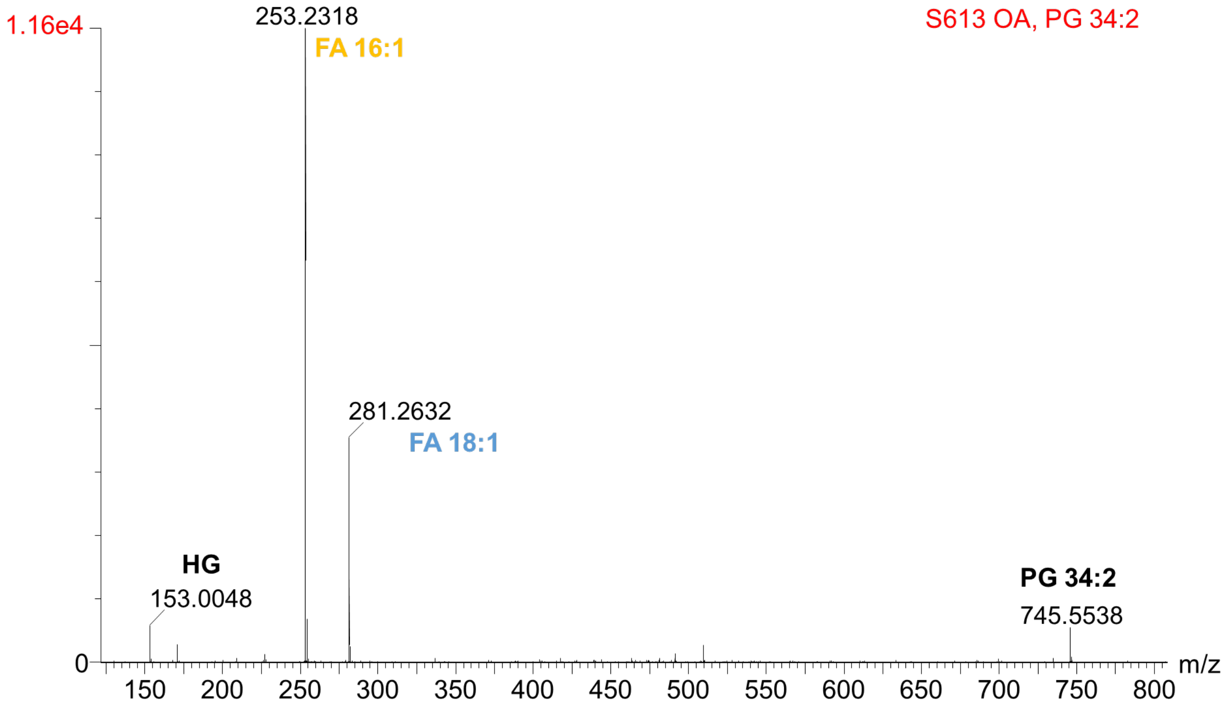

Figure S6g. MSMS fragmentation of PG 34:2 in condition S613 OA.

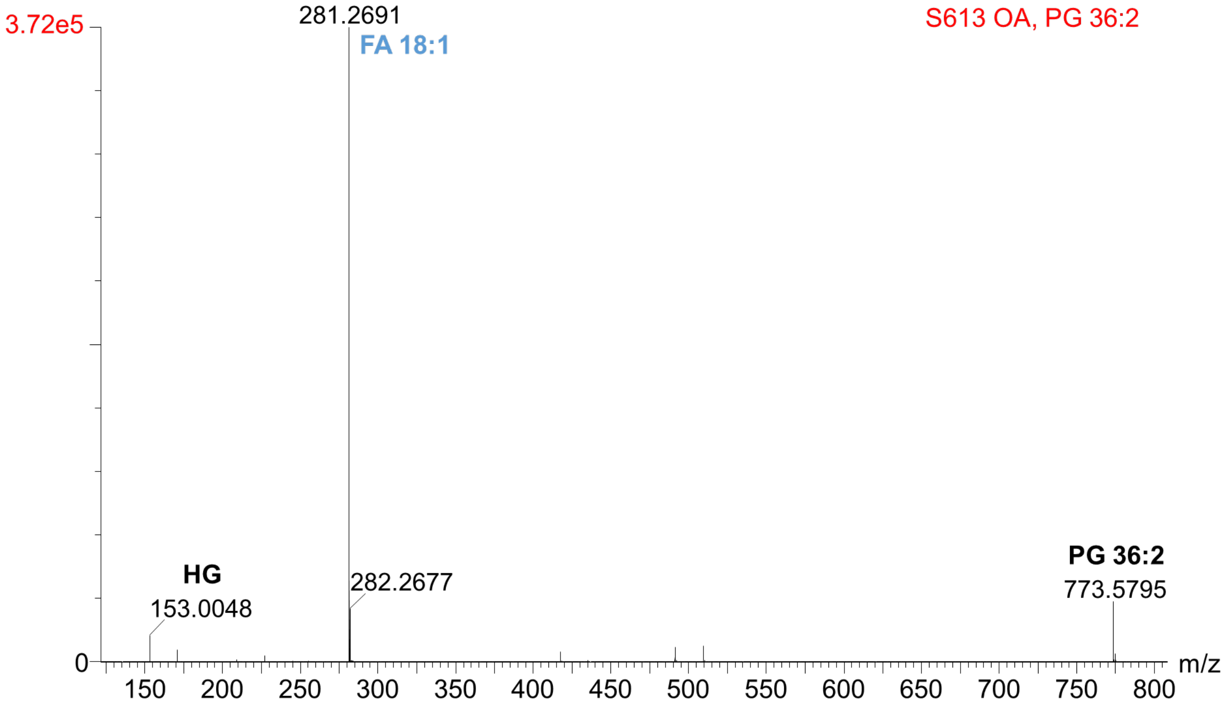

Figure S6h. MSMS fragmentation of PG 36:2 in condition S613 OA.

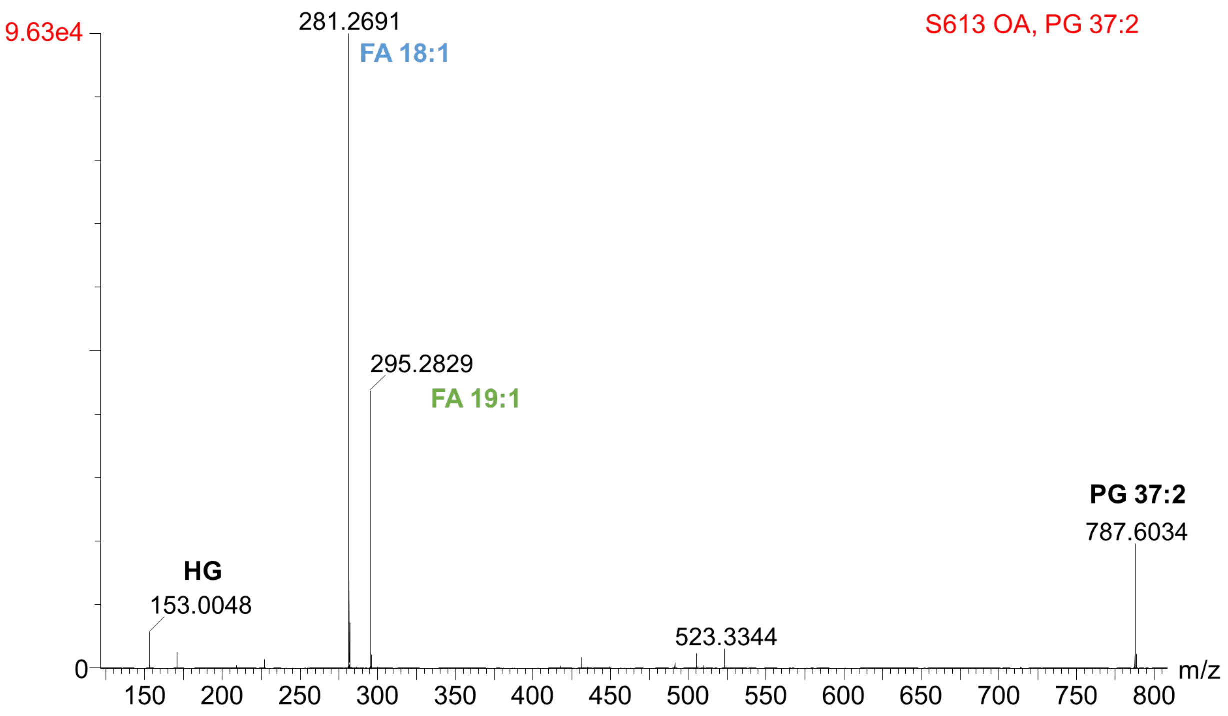

Figure S6i. MSMS fragmentation of PG 37:2 in condition S613 OA.

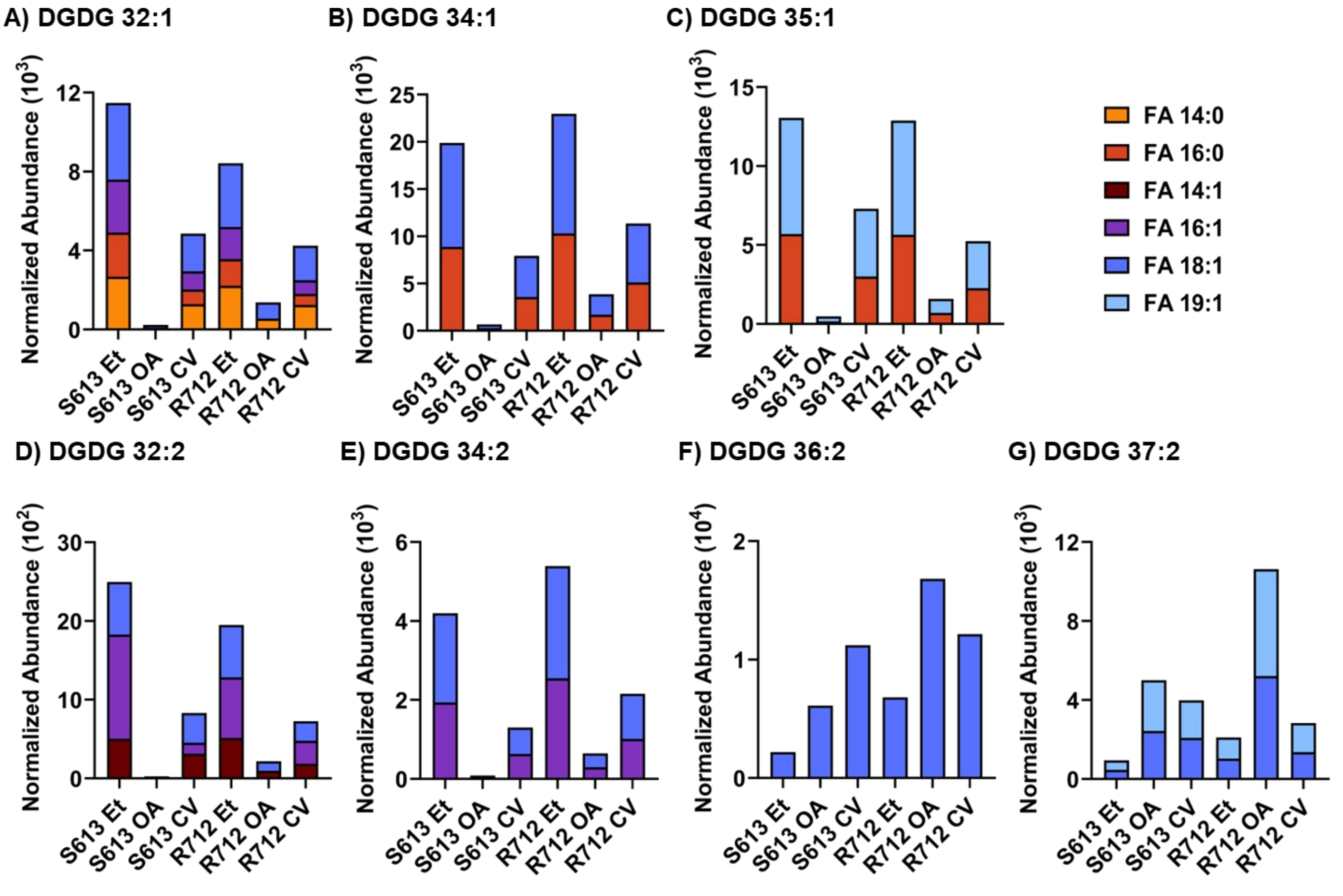

Figure S7. Targeted RPLC-IM-MS/MS of FA distributions of DGDG precursors.

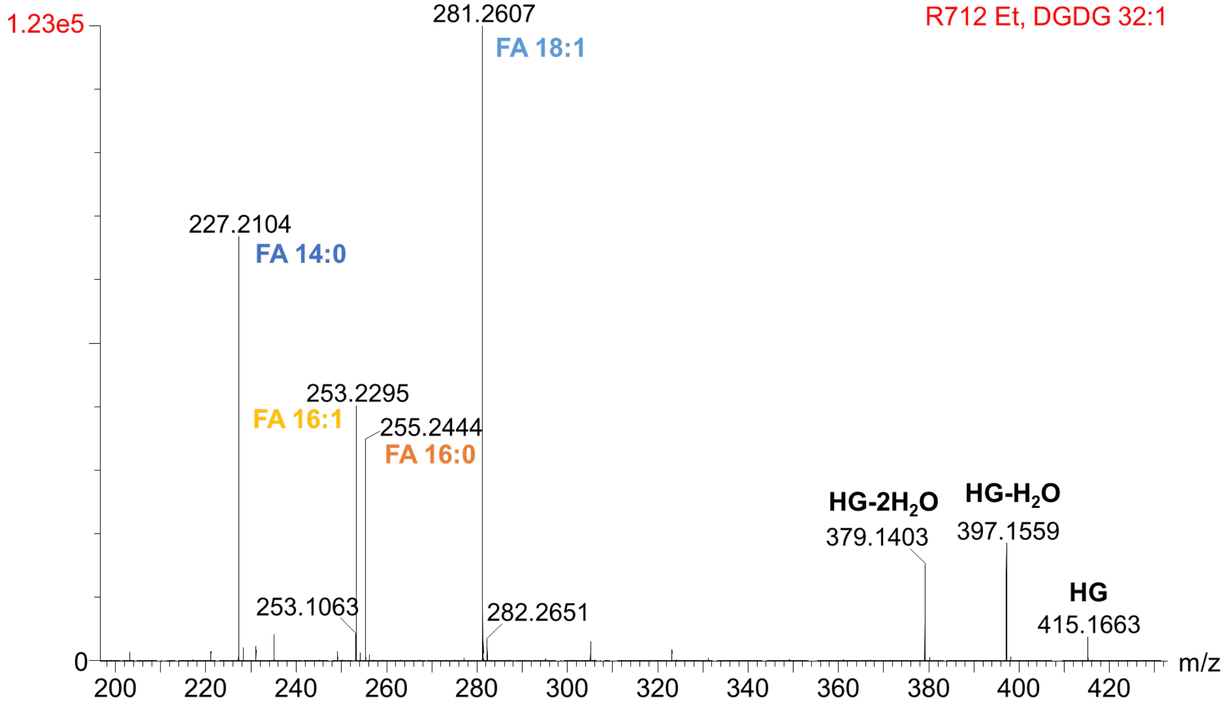

Figure S8a. MSMS fragmentation of DGDG 32:1 in condition R712 Et.

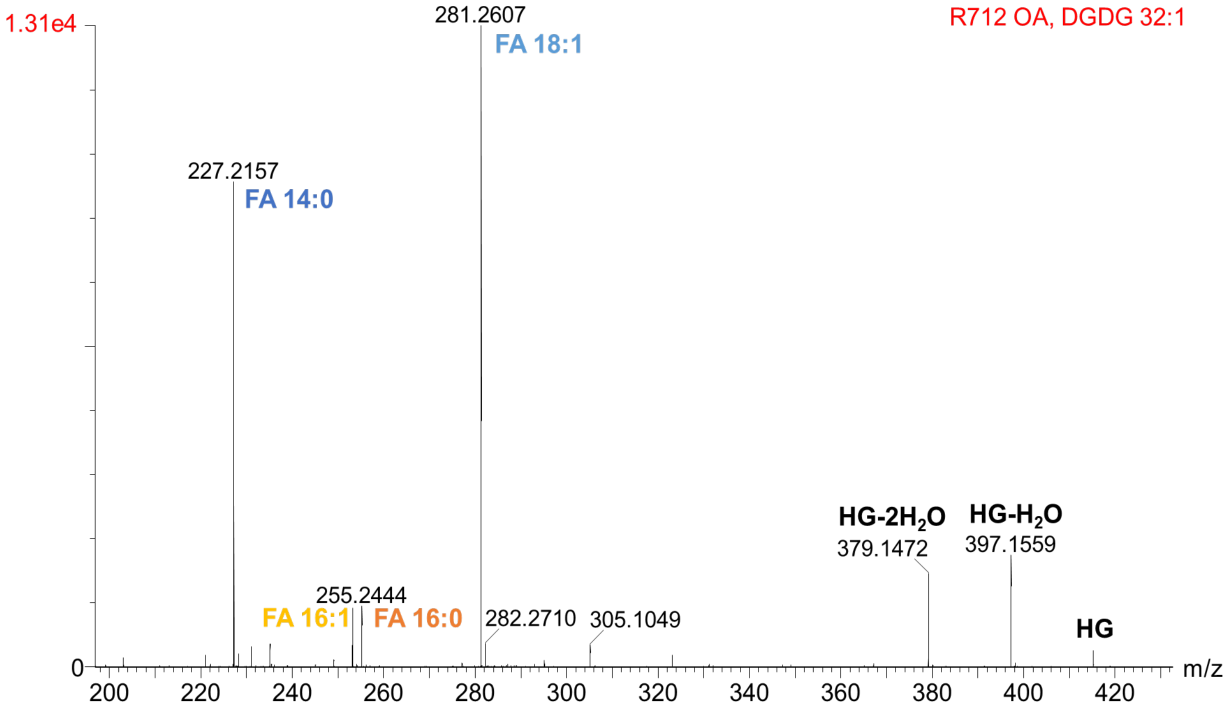

Figure S8b. MSMS fragmentation of DGDG 32:1 in condition R712 OA.

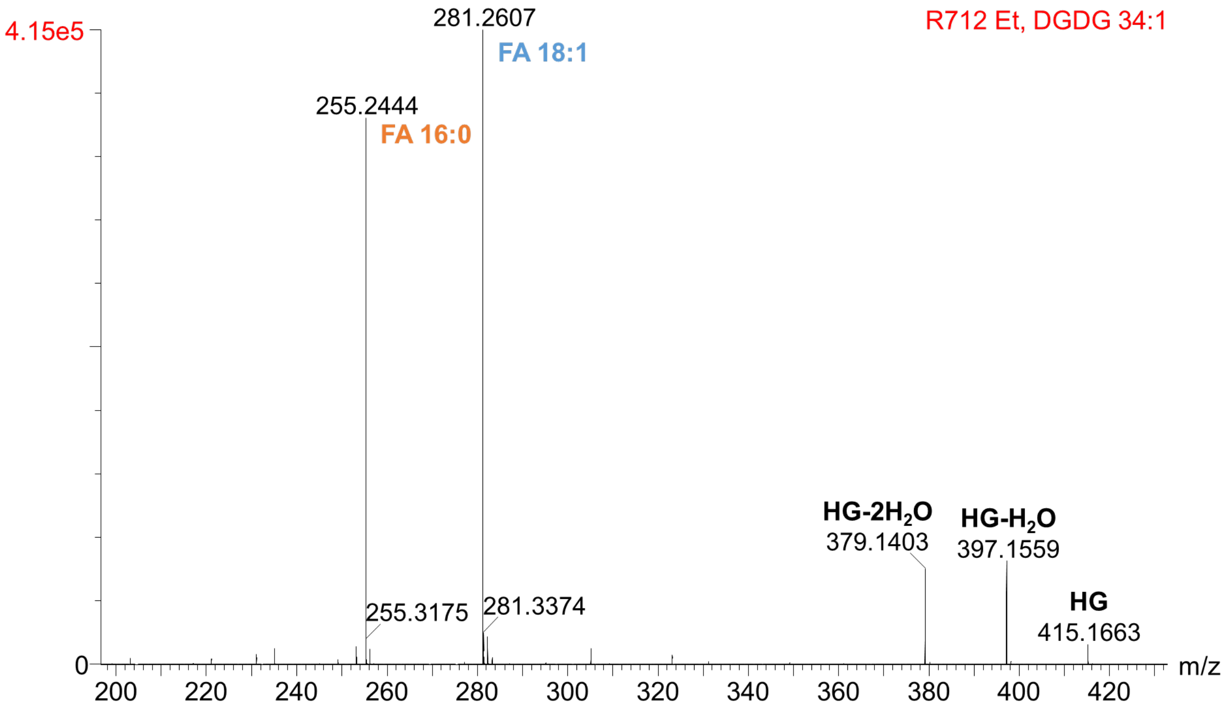

Figure S8c. MSMS fragmentation of DGDG 34:1 in condition R712 Et.

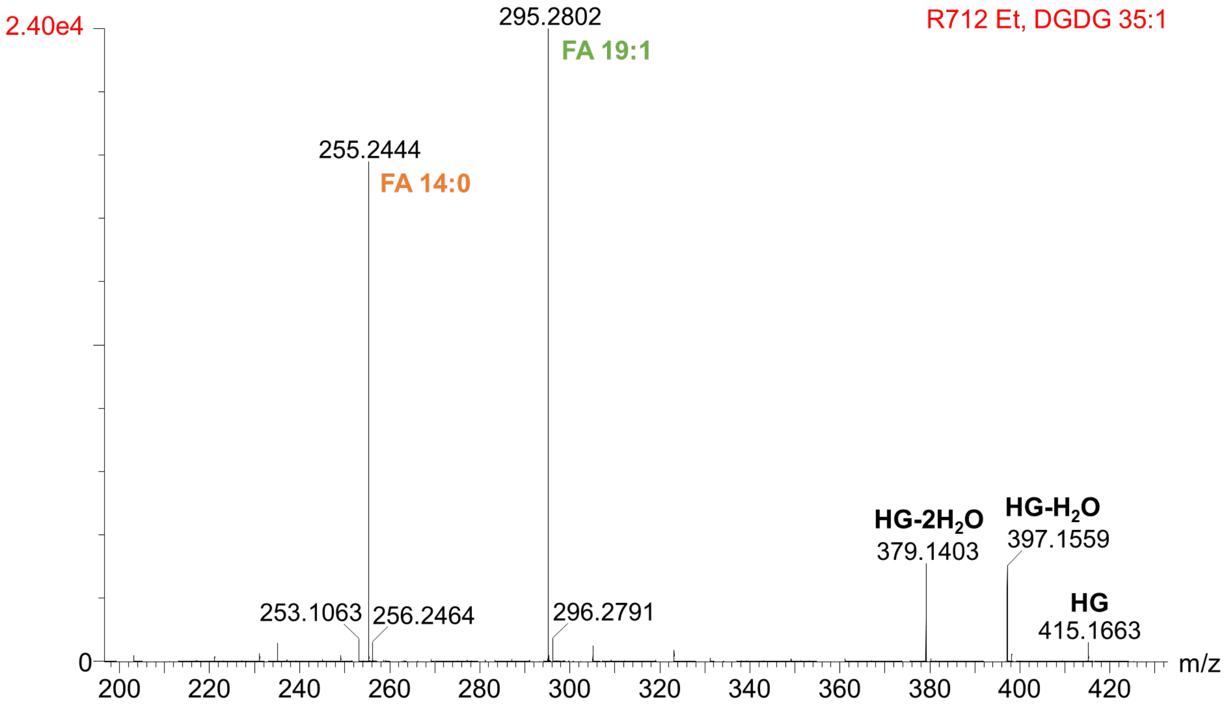

Figure S8d. MSMS fragmentation of DGDG 35:1 in condition R712 Et.

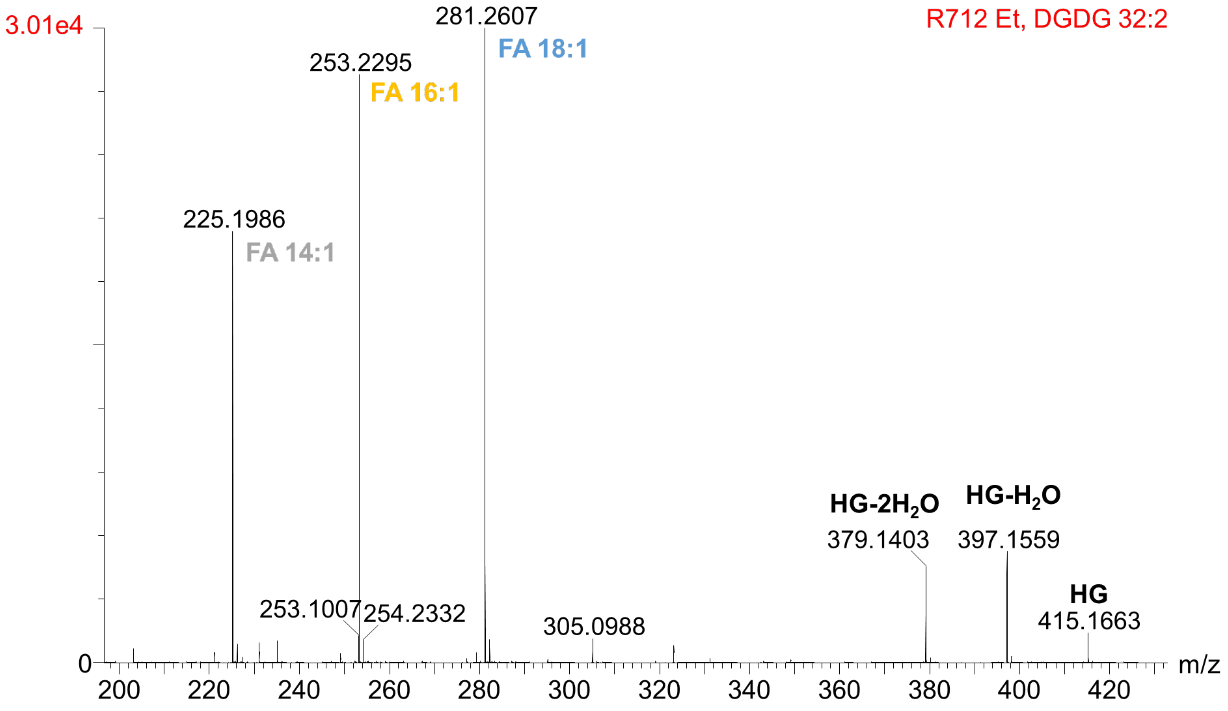

Figure S8e. MSMS fragmentation of DGDG 32:2 in condition R712 Et.

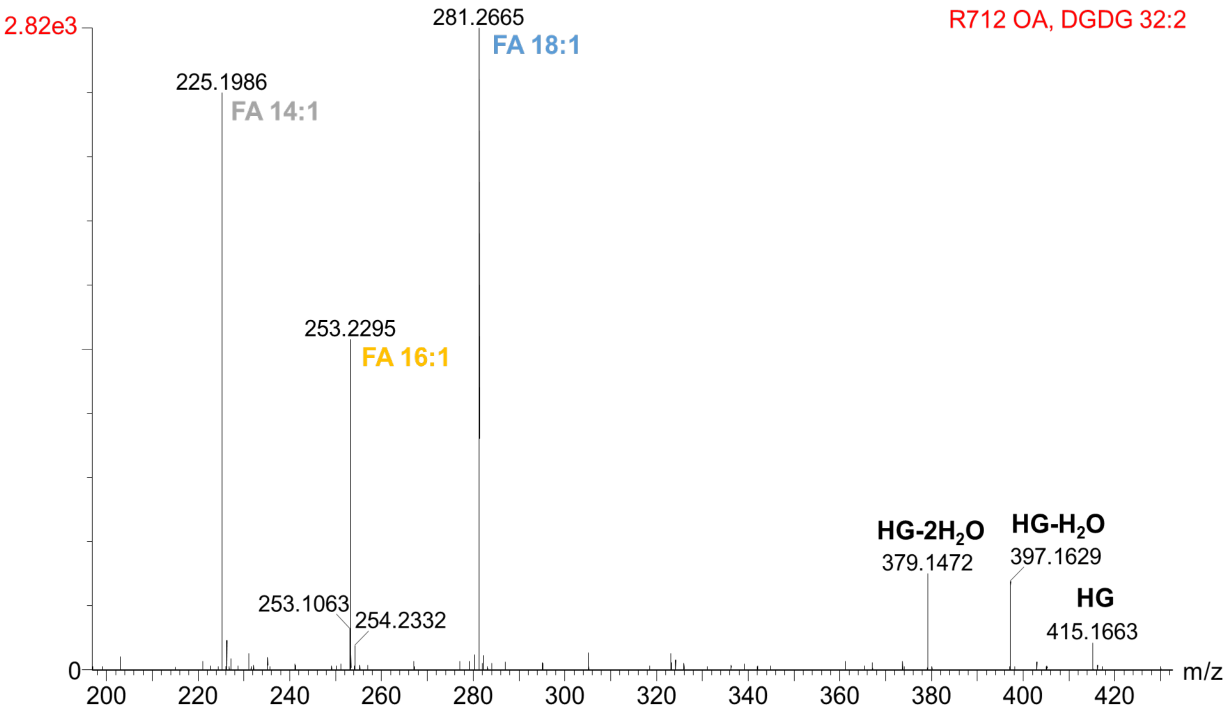

Figure S8f. MSMS fragmentation of DGDG 32:2 in condition R712 OA.

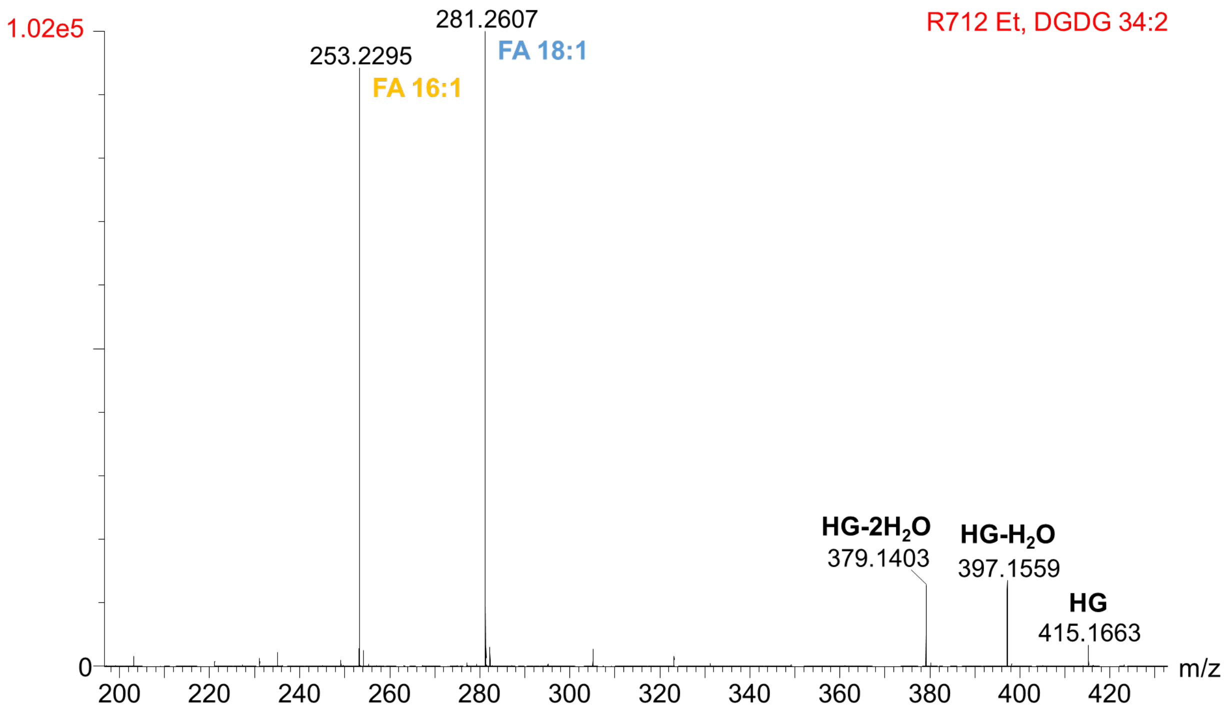

Figure S8g. MSMS fragmentation of DGDG 34:2 in condition R712 Et.

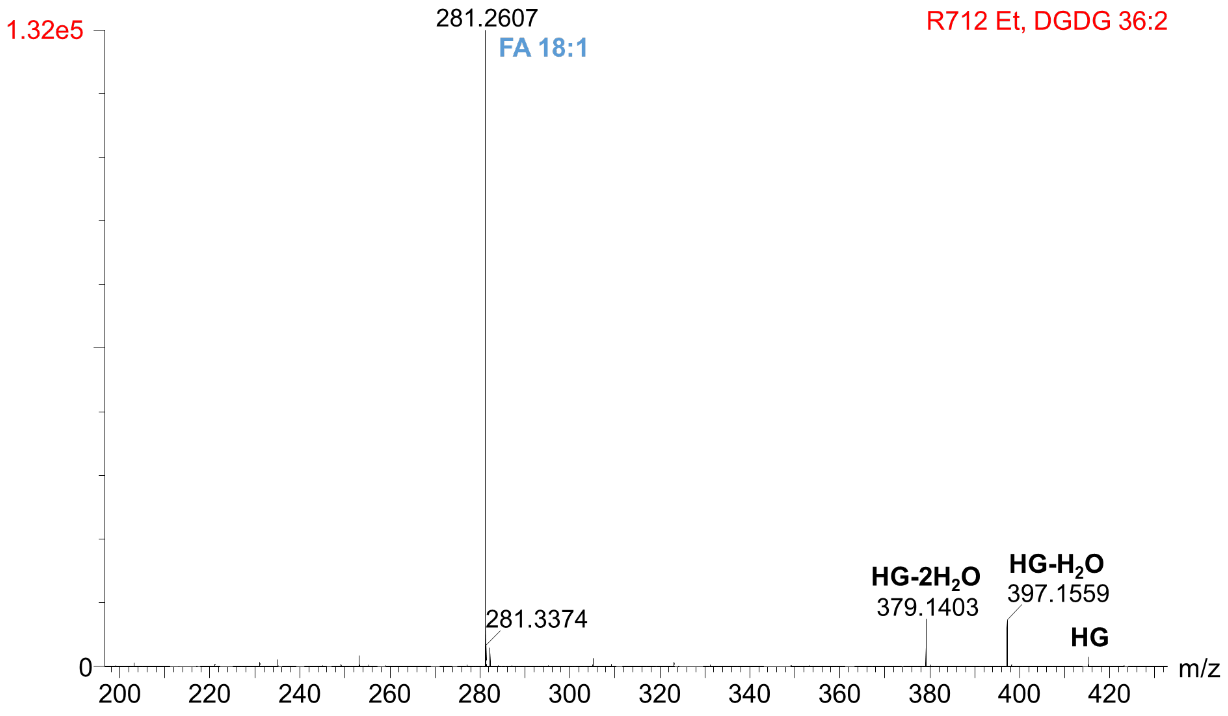

Figure S8h. MSMS fragmentation of DGDG 36:2 in condition R712 Et.

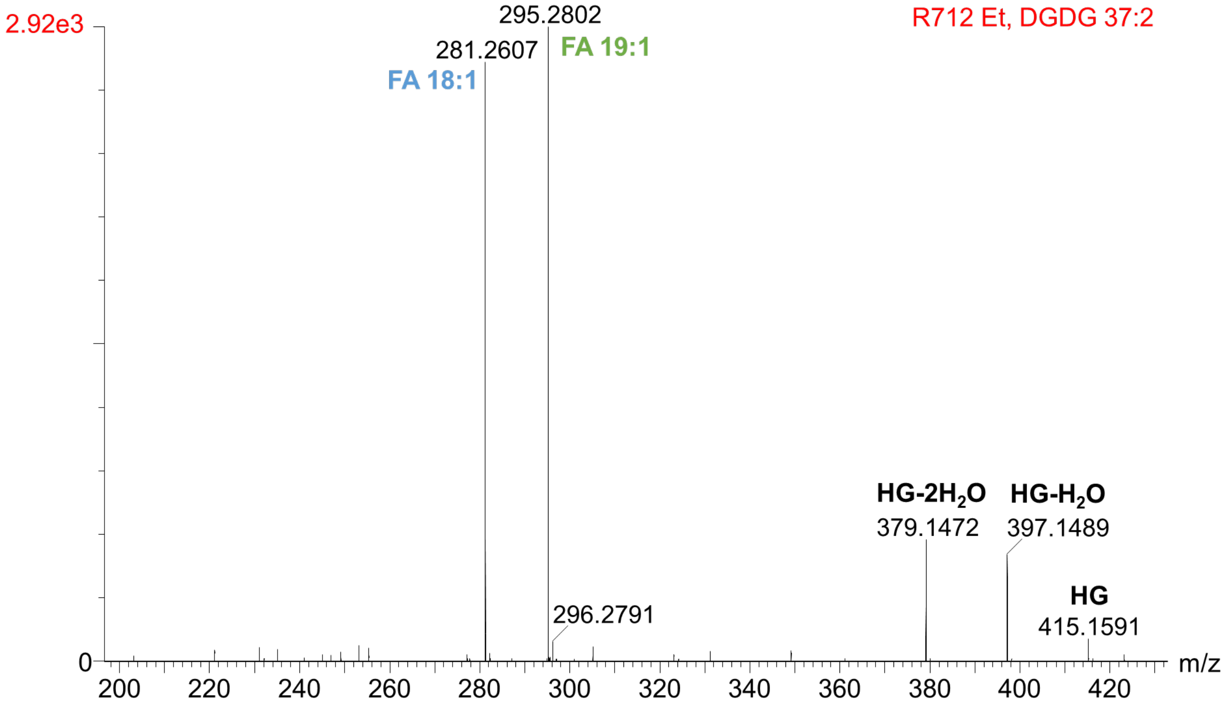

Figure S8i. MSMS fragmentation of DGDG 37:2 in condition R712 Et.

5. Determining Oleic Acid Incorporation

5a. Mixture of OA and CV

Table S11. IDs and mass errors (ppm) of FFA 18:1, 10-hydroxystearic acid, PG, and DGDG in HILIC-IM-MS data sets displaced in *SI Figures S9 and S11.*

Compound	Adduct	RT	Observed *m/z*	Exact *m/z*	Mass Error
**FFA 18:1**	$[M-H]^-$	0.71	281.2482	281.2486	-1.6
**10-HSA**	$[M-H]^-$	0.86	299.2584	299.2591	-2.2
**FA 18:1**	$[M-H]^-$	1.09	281.2482	281.2486	-1.6
**FA 18:1**	$[M-H]^-$	1.86	281.2482	281.2486	-1.6
**PG 32:1**	$[M-H]^-$	1.86	719.4873	719.4868	0.6
**PG 34:1**	$[M-H]^-$	1.83	747.5190	747.5181	1.2
**PG 35:1**	$[M-H]^-$	1.83	761.5331	761.5338	-1.0
**PG 32:2**	$[M-H]^-$	1.86	717.4728	717.4712	2.2
**PG 34:2**	$[M-H]^-$	1.83	745.5043	745.5025	2.4
**PG 36:2**	$[M-H]^-$	1.79	773.5346	773.5338	1.1
**PG 37:2**	$[M-H]^-$	1.79	787.5500	787.5494	0.7
**DGDG 32:1**	$[M+HCOO]^-$	1.09	935.5957	935.5949	0.9
**DGDG 34:1**	$[M+HCOO]^-$	1.09	963.6268	963.6262	0.7
**DGDG 35:1**	$[M+HCOO]^-$	1.09	977.6392	977.6418	-2.6
**DGDG 32:2**	$[M+HCOO]^-$	1.09	933.5814	933.5792	2.3
**DGDG 34:2**	$[M+HCOO]^-$	1.09	961.6104	961.6105	-0.1
**DGDG 36:2**	$[M+HCOO]^-$	1.05	989.6424	989.6418	0.6
**DGDG 37:2**	$[M+HCOO]^-$	1.05	1003.6558	1003.6575	-1.7

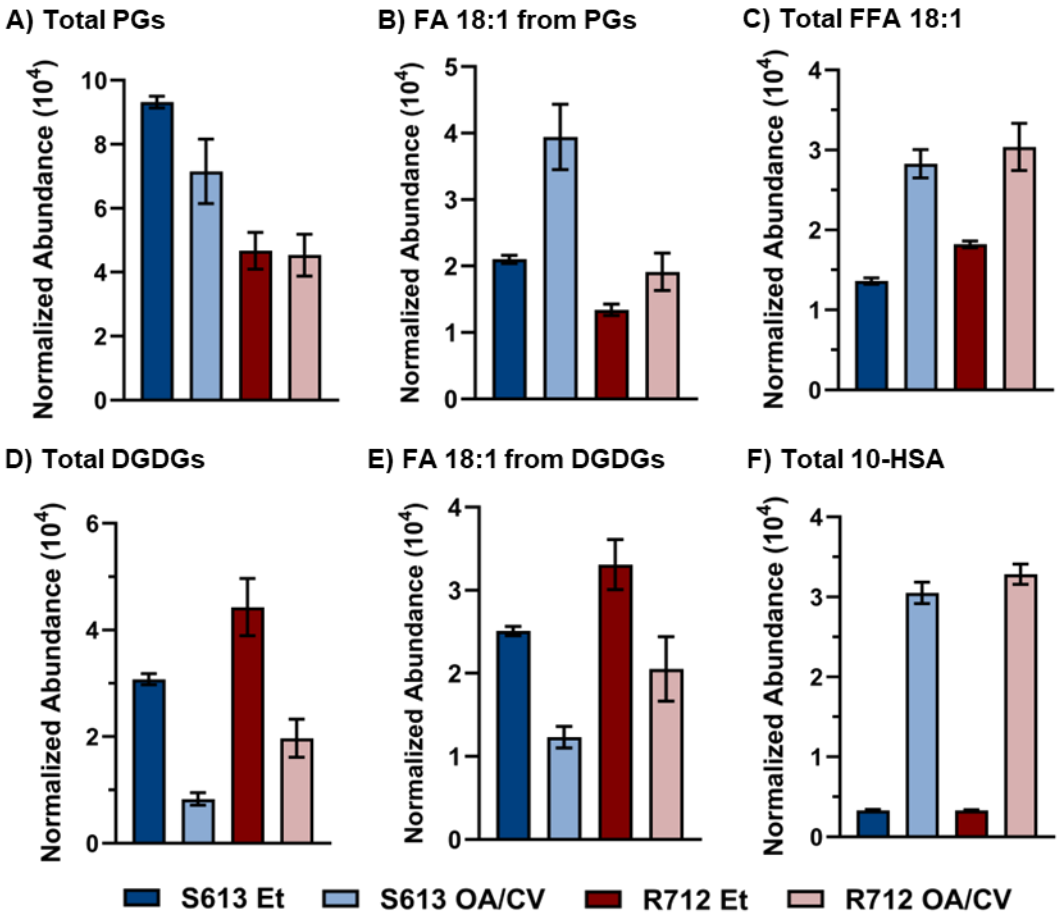

Figure S9: HILIC-IM-MS results of samples with a mix of oleic acid and *cis*-vaccenic acid of A) total PGs, B) total FA 18:1 on PG backbones, C) total free FA 18:1, D) total DGDGs, E) total FA 18:1 on DGDG backbones, and F) total 10-hydroxystearic acid.

Table S12. Summary table of the false-discovery rate adjusted *p*-values from multiple unpaired *t*-tests performed for the PG, DGDG, and FA 18:1 total abundance from the HILIC-IM-MS data set displayed in SI *Figures S9*.

**Condition 1**	**S613 Et**	**R712 Et**
**Condition 2**	**S613 OA/CV**	**R712OA/CV**
**PGs**	0.015	0.54
**PG FA 18:1**	0.0031	0.030
**DGDGs**	0.000033	0.0086
**DGDG FA 18:1**	0.00021	0.016
**FFA 18:1**	0.00021	0.0043

Table S13. Summary table of the log_2 fold changes for the PG, DGDG, and FA 18:1 total lipid class abundances from the HILIC-IM-MS data set displayed in *SI Figure S9*.

LOG2 FOLD CHANGE		
**Condition 1**	**S613 Et**	**R712 Et**
**Condition 2**	**S613 OA/CV**	**R712OA/CV**
**PGs**	-0.38	-0.04 ^{ns}
**PG FA 18:1**	0.91	0.51
**DGDGs**	-1.89	-1.17
**DGDG FA 18:1**	-1.03	-0.69
**FFA 18:1**	1.06	0.74

Note: the superscripted "ns" are the log_2 fold changes that are not statistically significant based on *SI Table S12*

Table S14. IDs and mass errors (ppm) of the CLs, LysylPGs, and MGDGs by HILIC-IM-MS displayed in *SI Figures S10 and S12*.

Compound	Adduct	RT	Observed *m/z*	Exact *m/z*	Mass Error
**CL 64:3**	$[M-2H]^{2-}$	2.76	672.4527	672.4553	-3.8
**CL 65:3**	$[M-2H]^{2-}$	2.79	679.4590	679.4632	-6.2
**CL 66:3**	$[M-2H]^{2-}$	2.79	686.4692	686.4710	-2.6
**CL 67:3**	$[M-2H]^{2-}$	2.79	693.4743	693.4788	-6.5
**CL 68:3**	$[M-2H]^{2-}$	2.79	700.4847	700.4866	-2.7
**CL 70:3**	$[M-2H]^{2-}$	2.79	714.4983	714.5023	-5.6
**CL 72:4**	$[M-2H]^{2-}$	2.79	727.5099	727.5101	-0.3
**CL 73:4**	$[M-2H]^{2-}$	2.84	734.5160	734.5179	-2.6
**LysylPG 30:1**	$[M-H]^{-}$	4.70	819.5496	819.5505	-1.1
**LysylPG 32:1**	$[M-H]^{-}$	4.65	847.5811	847.5818	-0.8
**LysylPG 34:1**	$[M-H]^{-}$	4.65	875.6121	875.6131	-1.2
**LysylPG 35:1**	$[M-H]^{-}$	4.62	889.6270	889.6287	-2.0
**MGDG 32:1**	$[M+CH3COO]^{-}$	0.55	787.5568	787.5577	-1.2
**MGDG 34:1**	$[M+CH3COO]^{-}$	0.55	815.5880	815.5890	-1.2
**MGDG 32:2**	$[M+CH3COO]^{-}$	0.55	785.5447	785.5420	3.4
**MGDG 34:2**	$[M+CH3COO]^{-}$	0.55	813.5718	813.5733	-1.9
**MGDG 36:2**	$[M+CH3COO]^{-}$	0.55	841.6044	841.6046	-0.2
**MGDG 37:2**	$[M+CH3COO]^{-}$	0.55	855.6205	855.6203	0.2

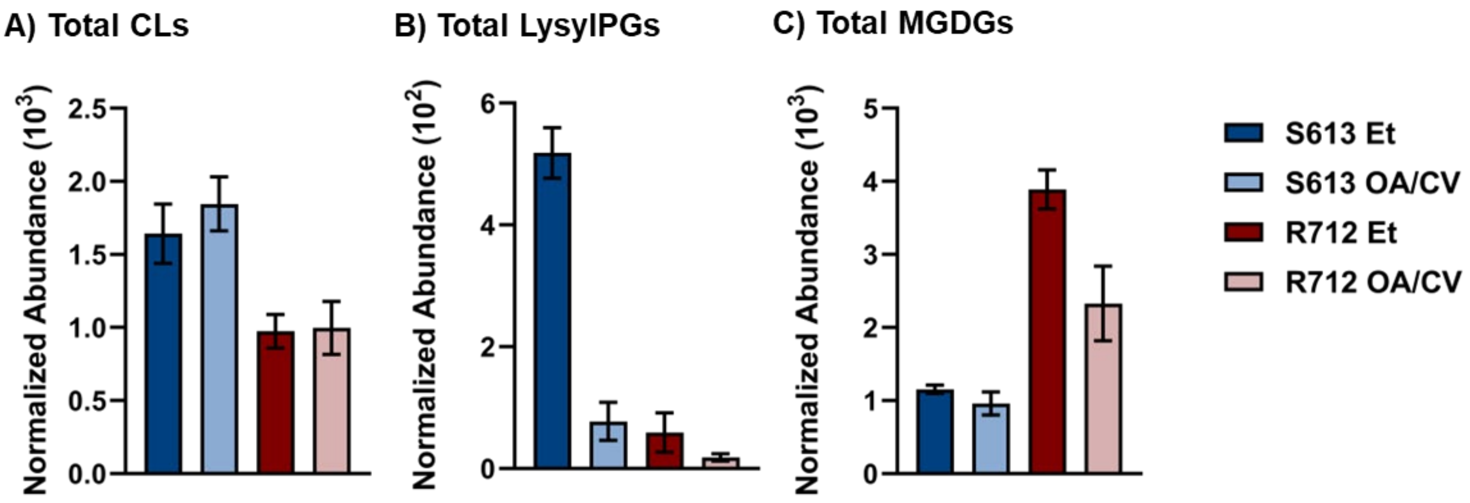

Figure S10. HILIC-IM-MS results of samples with a mix of oleic acid and *cis*-vaccenic acid of A) total CLs, B) total LysylPGs, and C) total MGDGs.

Table S15. Summary table of the false-discovery rate adjusted *p*-values from multiple unpaired *t*-tests and the $\log_2$ fold changes for the CL, LysylPG, and MGDG total lipid class abundances from the HILIC-IM-MS data set displayed in *SI Figure S10.*

**P-VALUES**		
**Condition 1**	**S613 Et**	**R712 Et**
**Condition 2**	**S613 OA/CV**	**R712OA/CV**
**CL**	0.11	0.54
**LysylPG**	0.00013	0.10
**MGDG**	0.061	0.015
**FOLD-CHANGE**		
**Condition 1**	**S613 Et**	**R712 Et**
**Condition 2**	**S613 OA/CV**	**R712OA/CV**
**CL**	-2.74	-1.70
**LysylPG**	0.17	0.03
**MGDG**	-1.89	-1.17

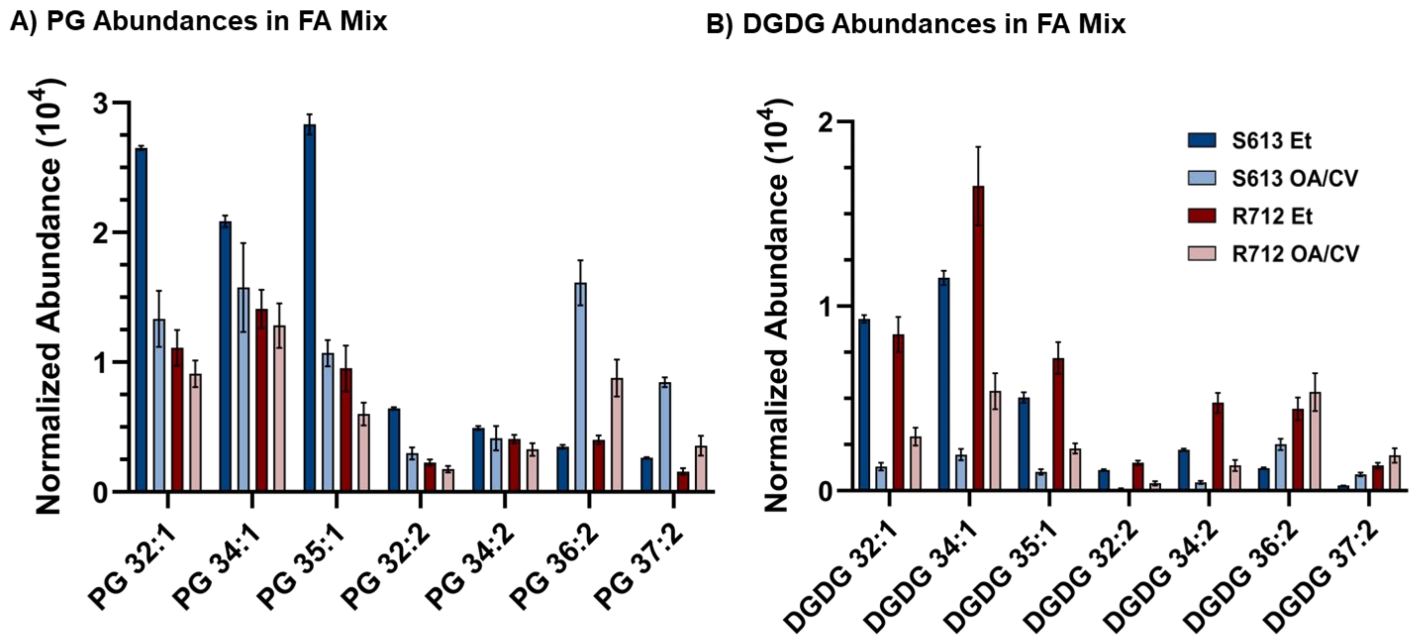

Figure S11. HILIC-IM-MS results of samples with a mix of oleic acid and *cis*-vaccenic acid of individual A) PG abundances and B) DGDG abundances.

Table S16. Summary table of the false-discovery rate adjusted *p*-values from multiple unpaired *t*-tests performed for the PG and DGDG precursor abundances from the HILIC-IM-MS data set displayed in SI *Figures S11*.

**Condition 1**	**S613 Et**	**R712 Et**
**Condition 2**	**S613 OA/CV**	**R712OA/CV**
**PG 32:1**	0.00019	0.098
**PG 34:1**	0.022	0.29
**PG 35:1**	<0.0001	0.064
**PG 32:2**	0.00012	0.081
**PG 34:2**	0.066	0.082
**PG 36:2**	0.00012	0.026
**PG 37:2**	<0.0001	0.034
**DGDG 32:1**	<0.0001	0.00045
**DGDG 34:1**	<0.0001	0.00050
**DGDG 35:1**	<0.0001	0.00045
**DGDG 32:2**	<0.0001	0.00045
**DGDG 34:2**	<0.0001	0.00045
**DGDG 36:2**	0.0020	0.077
**DGDG 37:2**	0.00066	0.031

Table S17. Summary table of the $\log_2$ fold changes for the PG and DGDG precursor abundances from the HILIC-IM-MS data set displayed in *SI Figure S11*.

**LOG2 FOLD CHANGE**		
**Condition 1**	**S613 Et**	**R712 Et**
**Condition 2**	**S613 OA/CV**	**R712OA/CV**
**PG 32:1**	-0.99	-0.29 ^{ns}
**PG 34:1**	-0.40	-0.14 ^{ns}
**PG 35:1**	-1.40	-0.66 ^{ns}
**PG 32:2**	-1.11	-0.37 ^{ns}
**PG 34:2**	-0.25 ^{ns}	-0.32 ^{ns}
**PG 36:2**	2.21	1.13
**PG 37:2**	1.68	1.18
**DGDG 32:1**	-2.82	-1.53
**DGDG 34:1**	-2.55	-1.61
**DGDG 35:1**	-2.31	-1.65
**DGDG 32:2**	-3.28	-1.90
**DGDG 34:2**	-2.29	-1.80
**DGDG 36:2**	1.04	0.27 ^{ns}
**DGDG 37:2**	1.63	0.48

Note: the superscripted "ns" are the $\log_2$ fold changes that are not statistically significant based on *SI Table S16*.

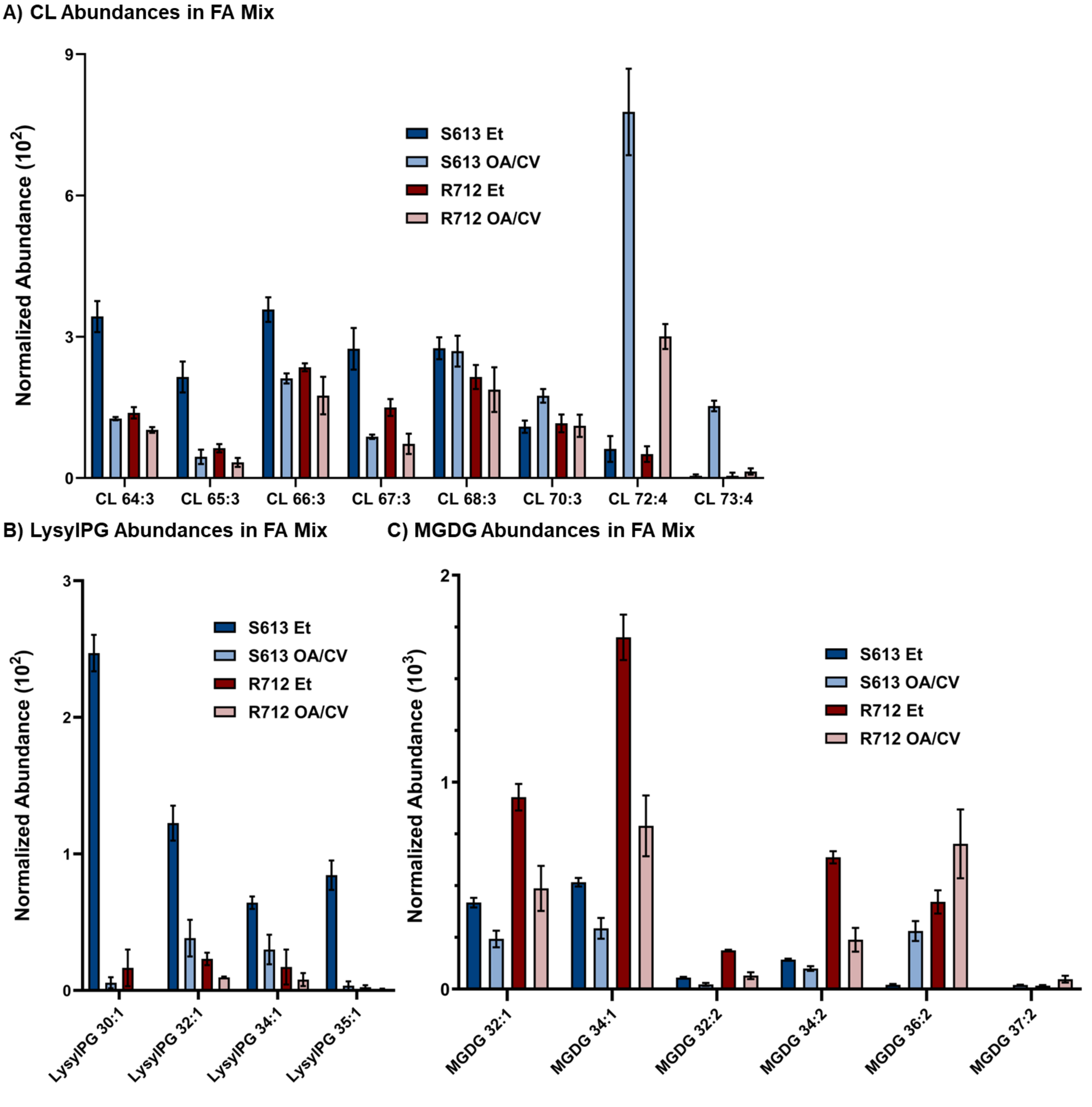

Figure S12. HILIC-IM-MS results of samples with a mix of oleic acid and *cis*-vaccenic acid of individual A) CL abundances, B) LysylPG abundances, and C) MGDG abundances.

Table S18. Summary table of the false-discovery rate adjusted *p*-values from multiple unpaired *t*-tests performed for the CL, LysylPG, and MGDG precursor abundances from the HILIC-IM-MS data set displayed in SI *Figures S12*.

**Condition 1**	**S613 Et**	**R712 Et**
**Condition 2**	**S613 OA/CV**	**R712OA/CV**
**CL 64:3**	0.00012	0.013
**CL 65:3**	0.00026	0.016
**CL 66:3**	0.00023	0.053
**CL 67:3**	0.00033	0.013
**CL 68:3**	0.11	0.26
**CL 70:3**	0.00064	0.41
**CL 72:4**	0.00011	0.00067
**CL 73:4**	<0.0001	0.12
**LysylPG 30:1**	<0.0001	0.16
**LysylPG 32:1**	0.0020	0.022
**LysylPG 34:1**	0.0076	0.24
**LysylPG 35:1**	0.00049	0.18
**MGDG 32:1**	0.0036	0.00099
**MGDG 34:1**	0.0036	0.00035
**MGDG 32:2**	0.0036	0.00022
**MGDG 34:2**	0.0039	0.00023
**MGDG 36:2**	0.0024	0.0089
**MGDG 37:2**	0.00037	0.0062

Table S19. Summary table of the log_2 fold changes for the CL, LysylPG, and MGDG precursor abundances from the HILIC-IM-MS data set displayed in *SI Figure S12*.

**LOG2 FOLD CHANGE**		
**Condition 1**	**S613 Et**	**R712 Et**
**Condition 2**	**S613 OA/CV**	**R712OA/CV**
**CL 64:3**	-1.44	-0.44
**CL 65:3**	-2.25	-0.93
**CL 66:3**	-0.76	-0.43^{ns}
**CL 67:3**	-1.65	-1.05
**CL 68:3**	-0.03^{ns}	-0.19^{ns}
**CL 70:3**	0.68	-0.07^{ns}
**CL 72:4**	3.65	2.56
**CL 73:4**	4.98	1.55^{ns}
**LysylPG 30:1**	-5.45	N/A
**LysylPG 32:1**	-1.68	-1.27
**LysylPG 34:1**	-1.10	-1.10^{ns}
**LysylPG 35:1**	-4.59	-2.04^{ns}
**MGDG 32:1**	-0.79	-0.93
**MGDG 34:1**	-0.82	-1.11
**MGDG 32:2**	-1.29	-1.53
**MGDG 34:2**	-0.52	-1.42
**MGDG 36:2**	3.75	0.74
**MGDG 37:2**	N/A	1.54

Note: the superscripted "ns" are the log_2 fold changes that are not statistically significant based on *SI Table S18* and the "N/A" indicates the absence of the lipid species in one or both of the conditions being compared

5b. OzID Fragmentation

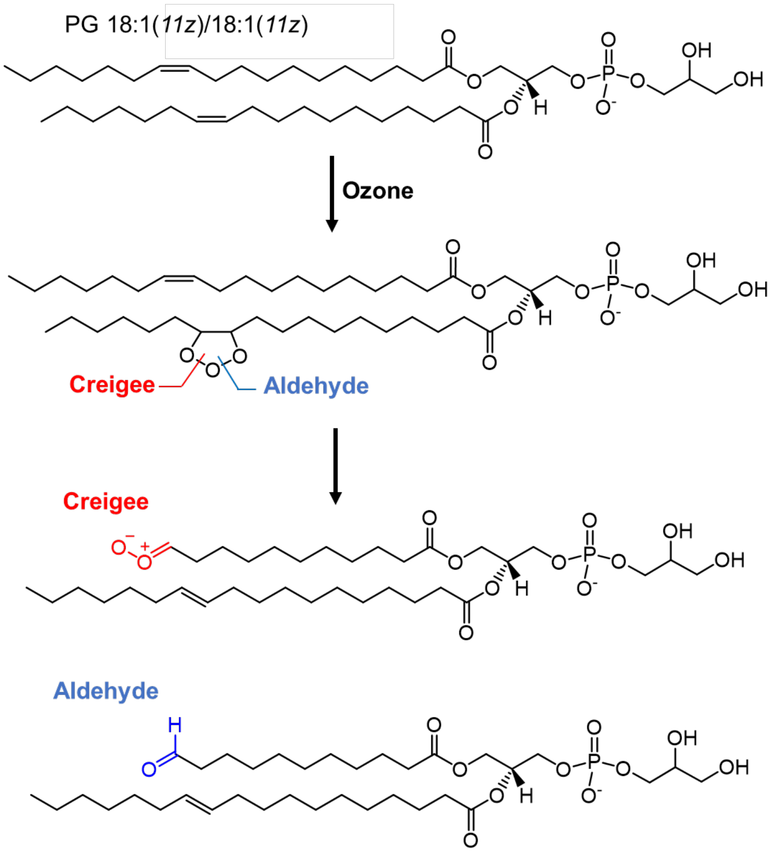

Figure S13: The ozonolysis mechanism in which ozone will react with a double bond in a single fatty acyl tail and form an ozonide ring that will break apart into a Creigee and aldehyde fragment.

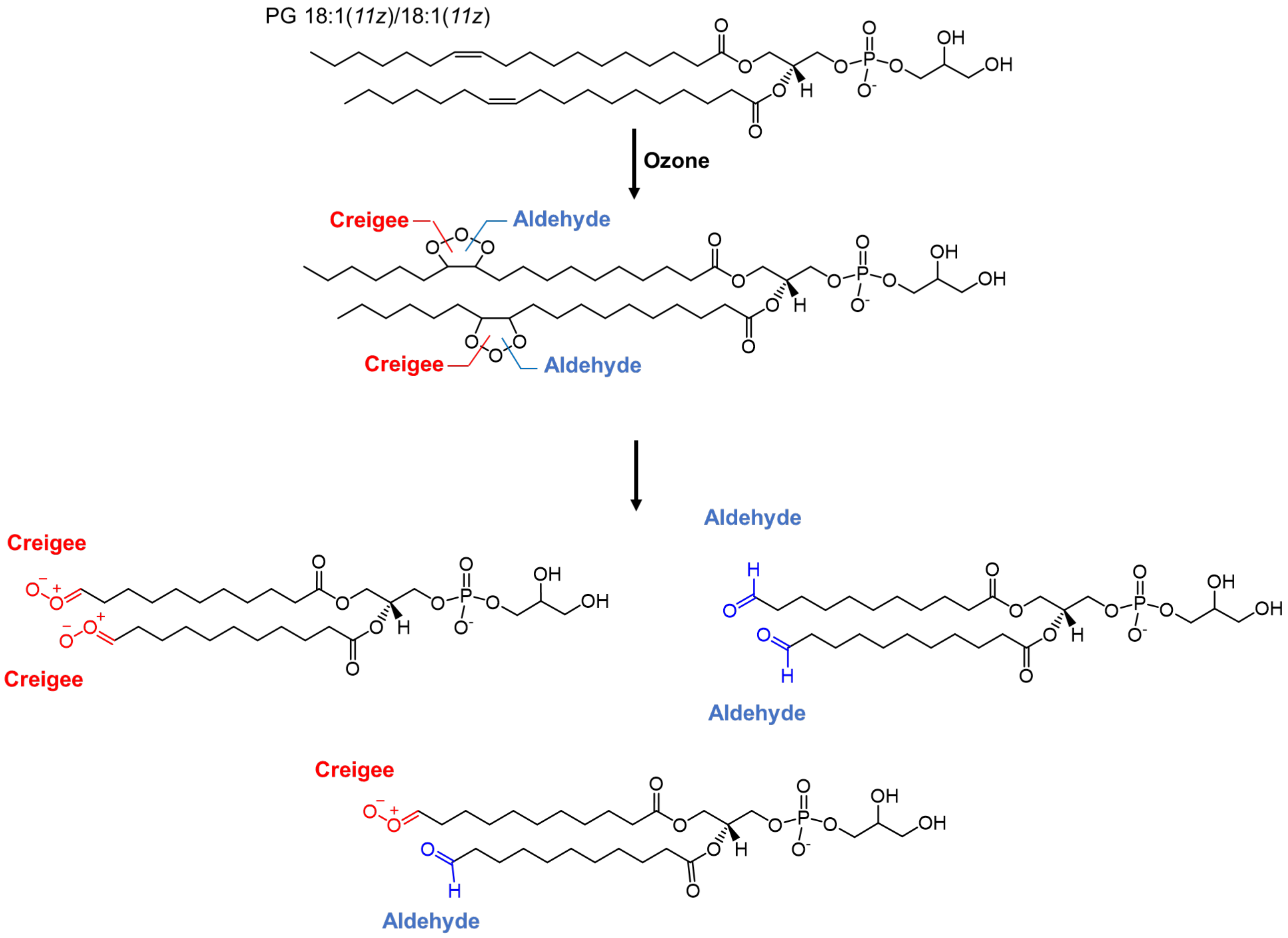

Figure S14: The OzID mechanism in which ozone will react with the double bonds in both fatty acyl tails and form an ozonide ring that will break apart into Creigee and aldehyde fragments of different combinations.

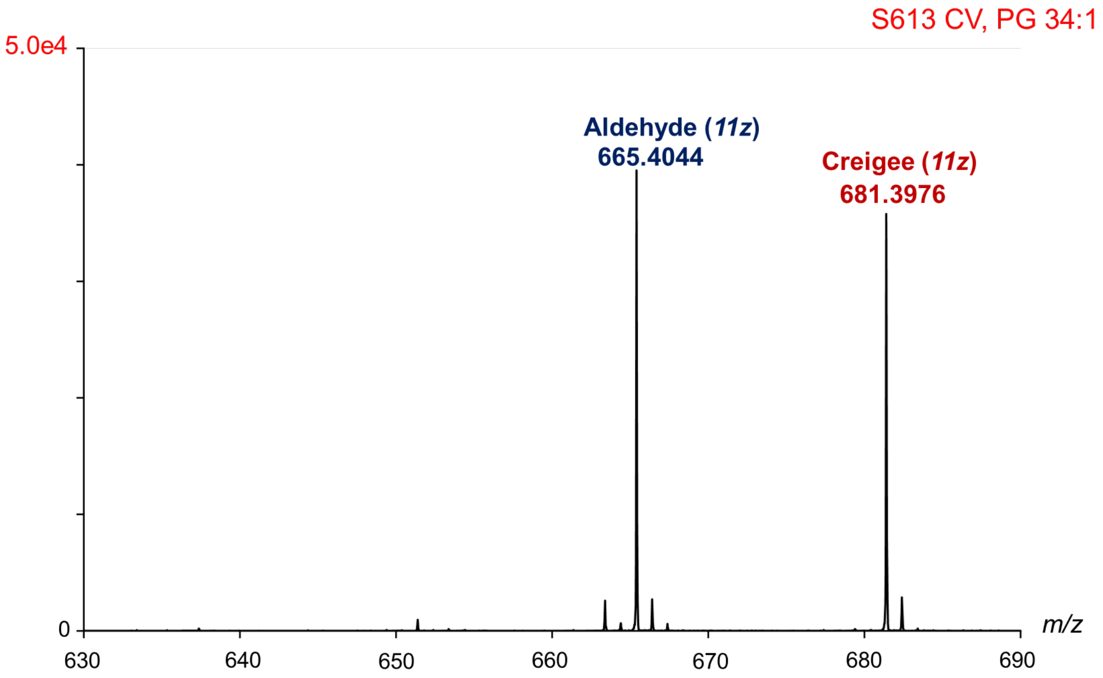

Figure S15a. MSMS fragmentation of PG 34:1 OzID fragments in condition S613 CV.

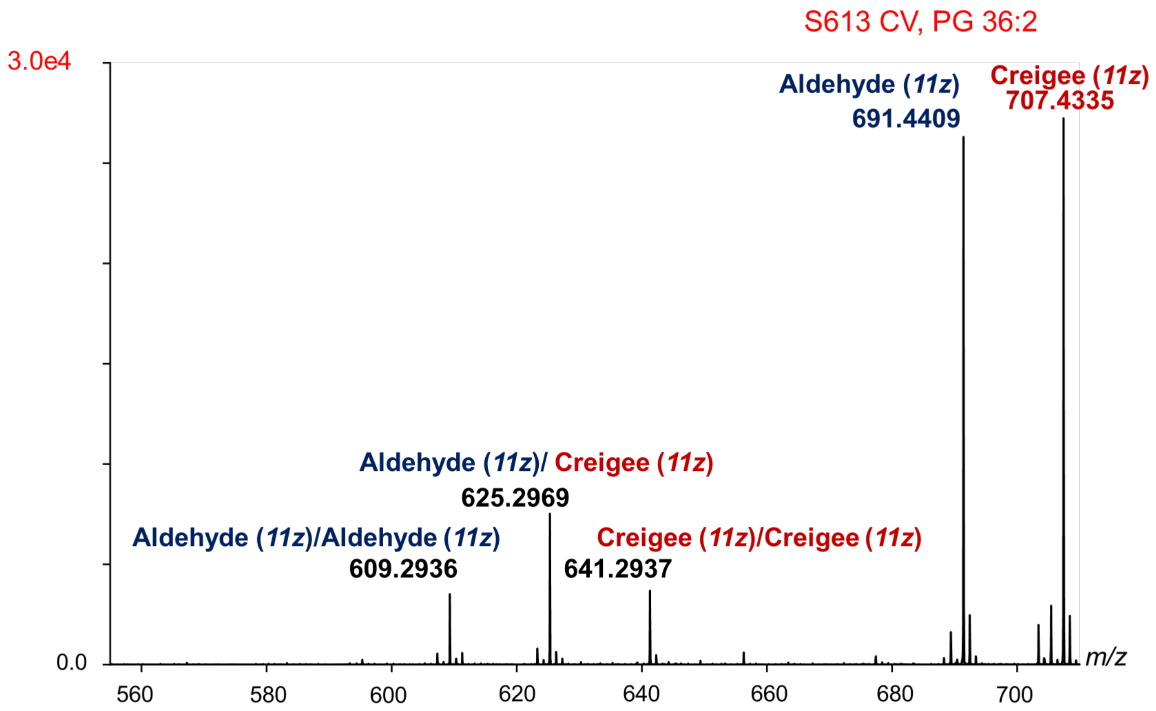

Figure S15b. OzID fragmentation of PG 36:2 in condition S613 CV.

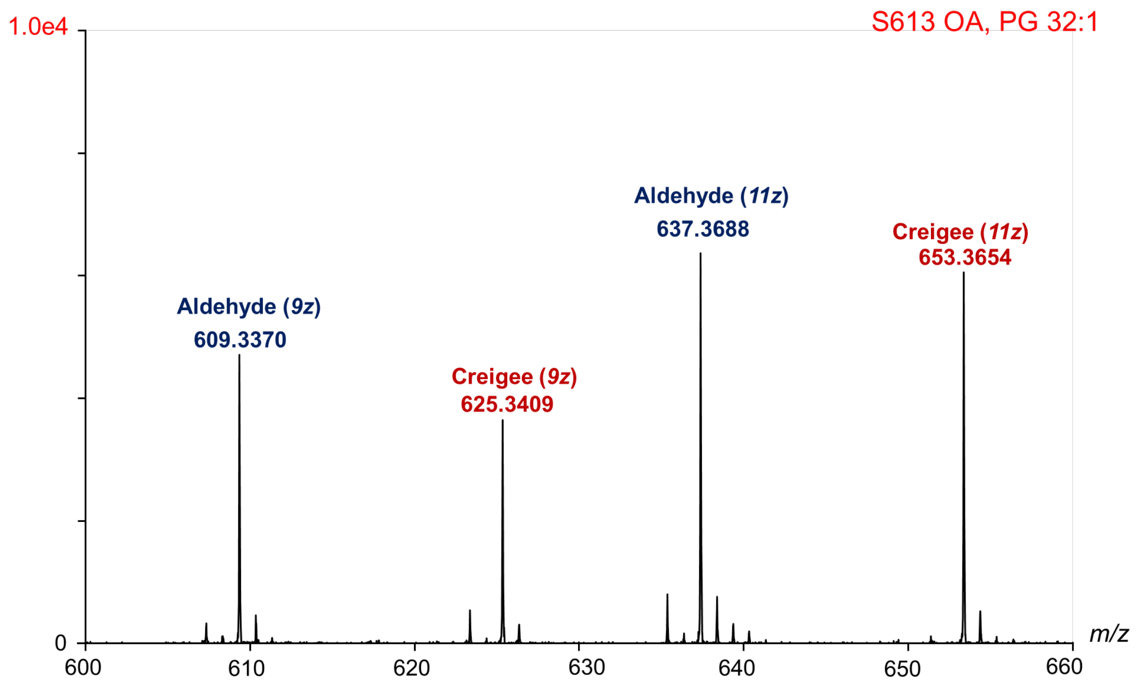

Figure S15c. OzID fragmentation of PG 32:1 in condition S613 OA.

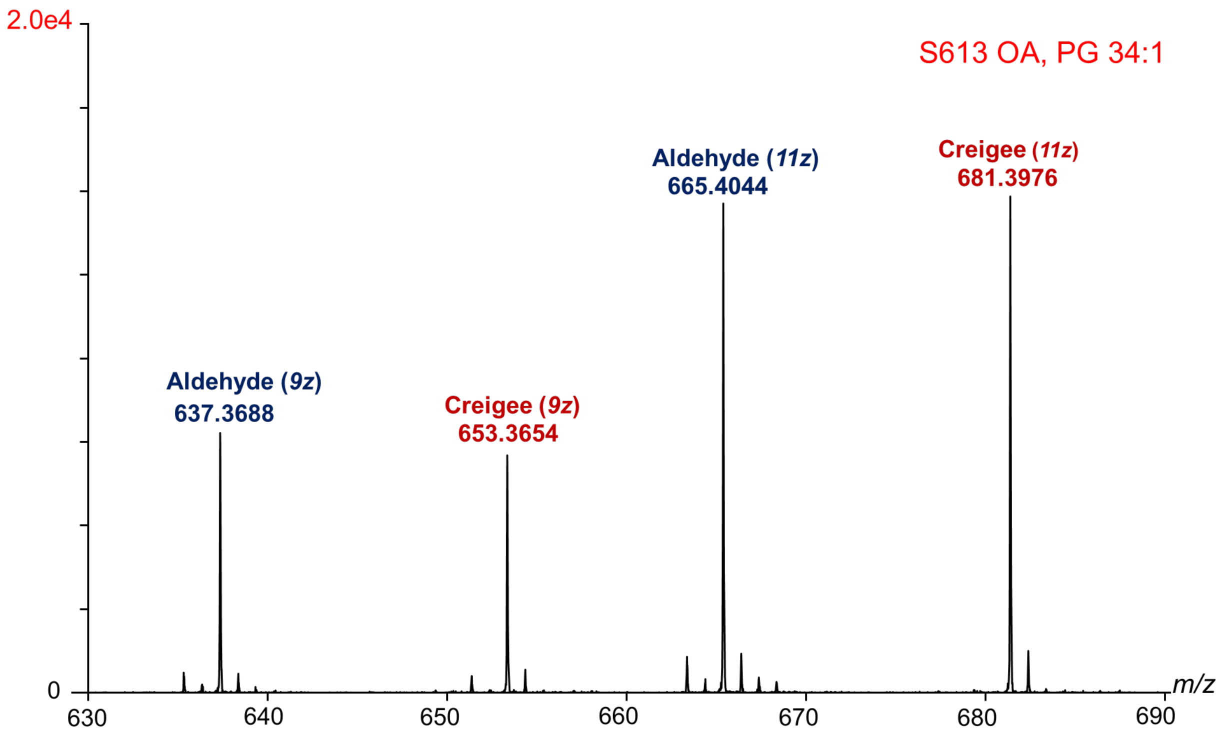

Figure S15d. OzID fragmentation of PG 34:1 in condition S613 OA.

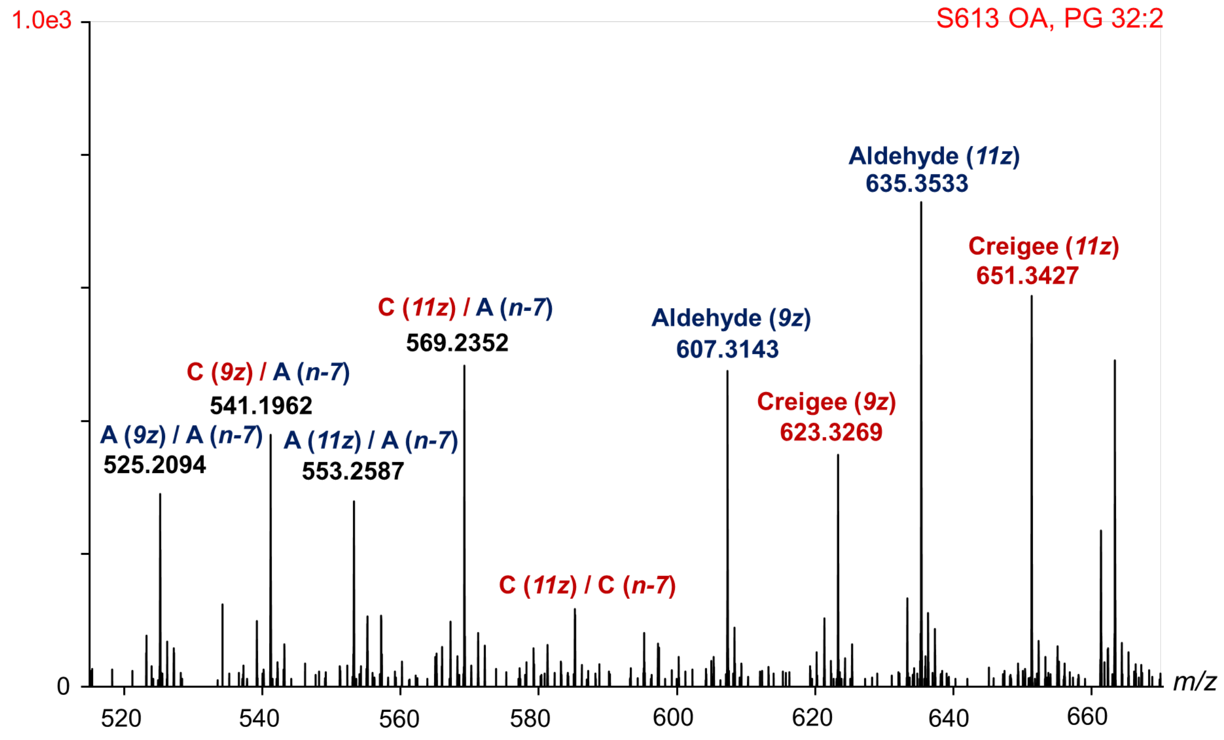

Figure S15e. OzID fragmentation of PG 32:2 in condition S613 OA.

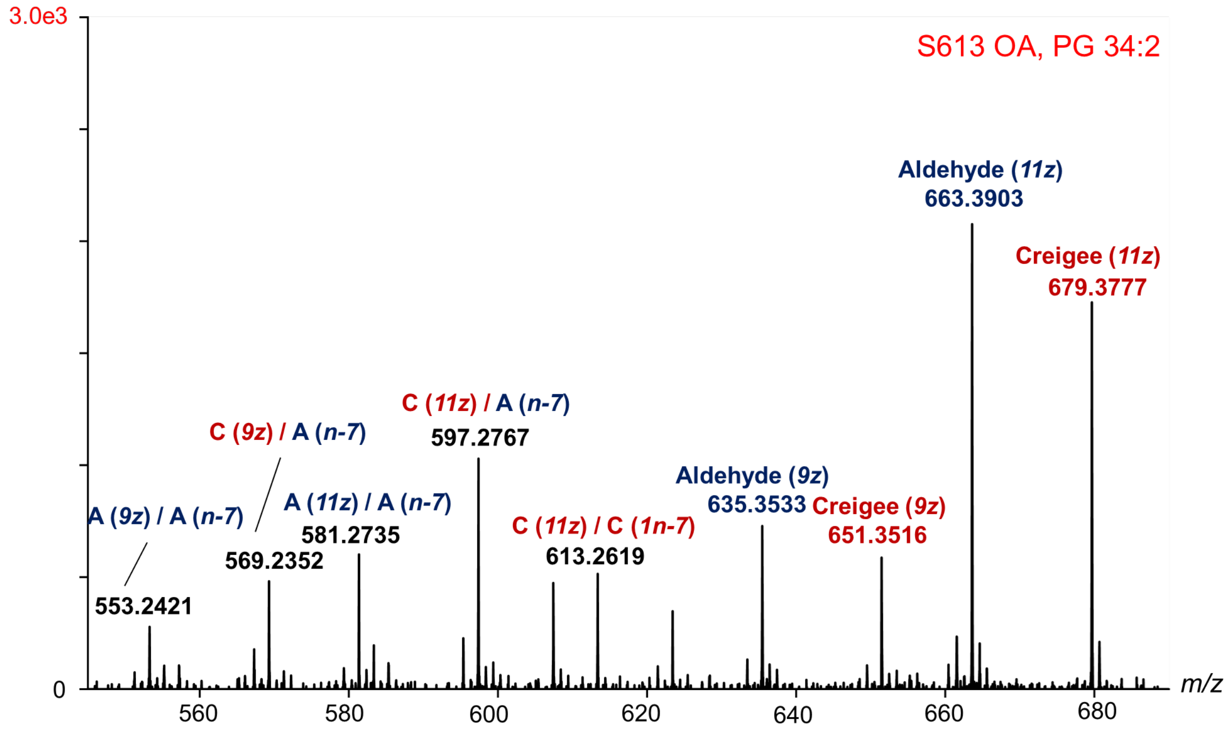

Figure S15f. OzID fragmentation of PG 34:2 in condition S613 OA.

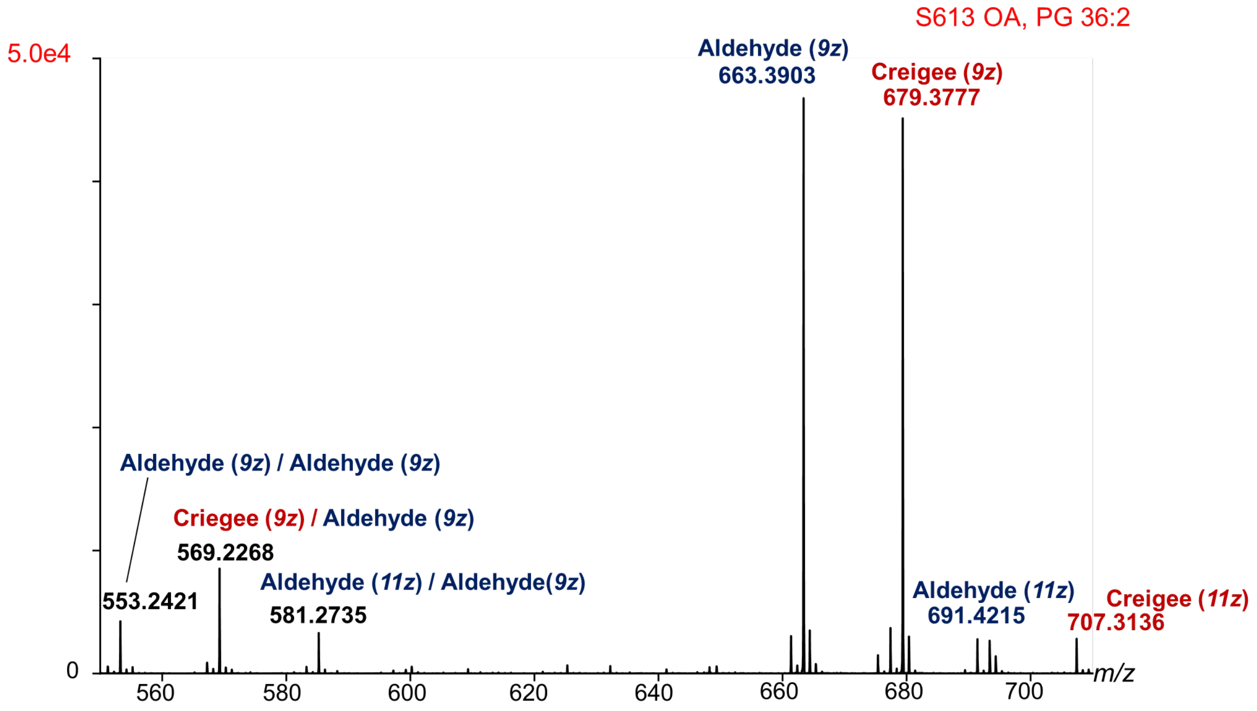

Figure S15g. OzID fragmentation of PG 36:2 in condition S613 OA.

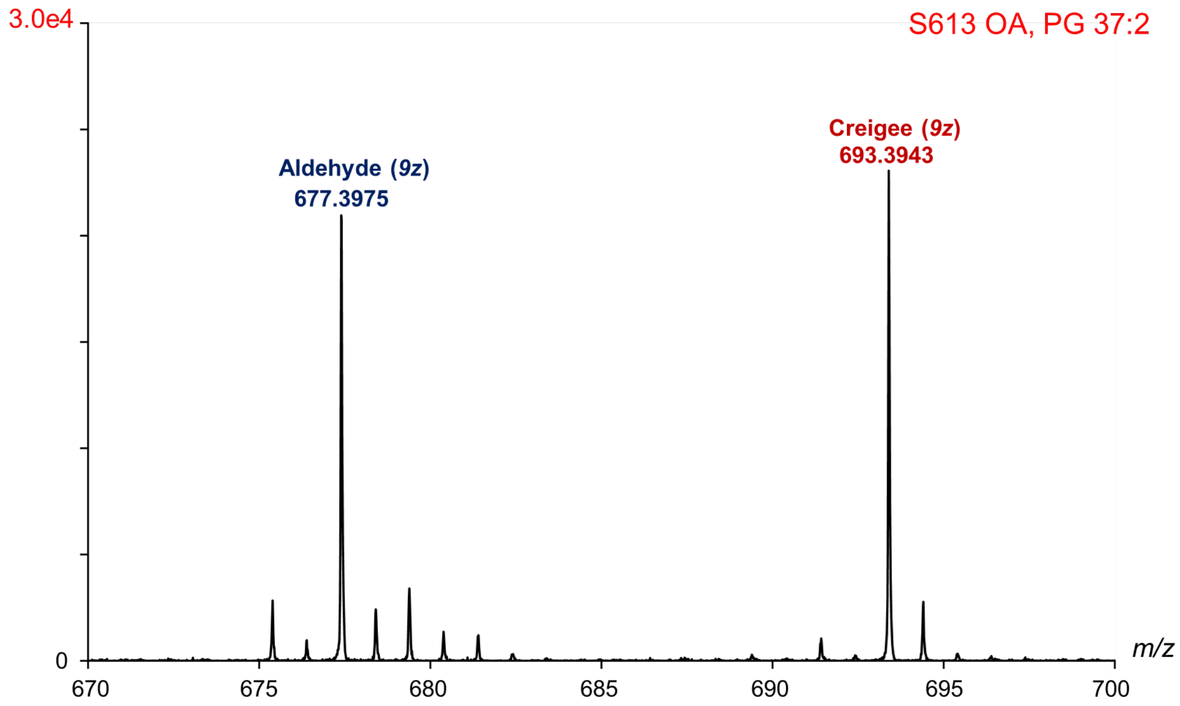

Figure S15h. OzID fragmentation of PG 37:2 in condition S613 OA.

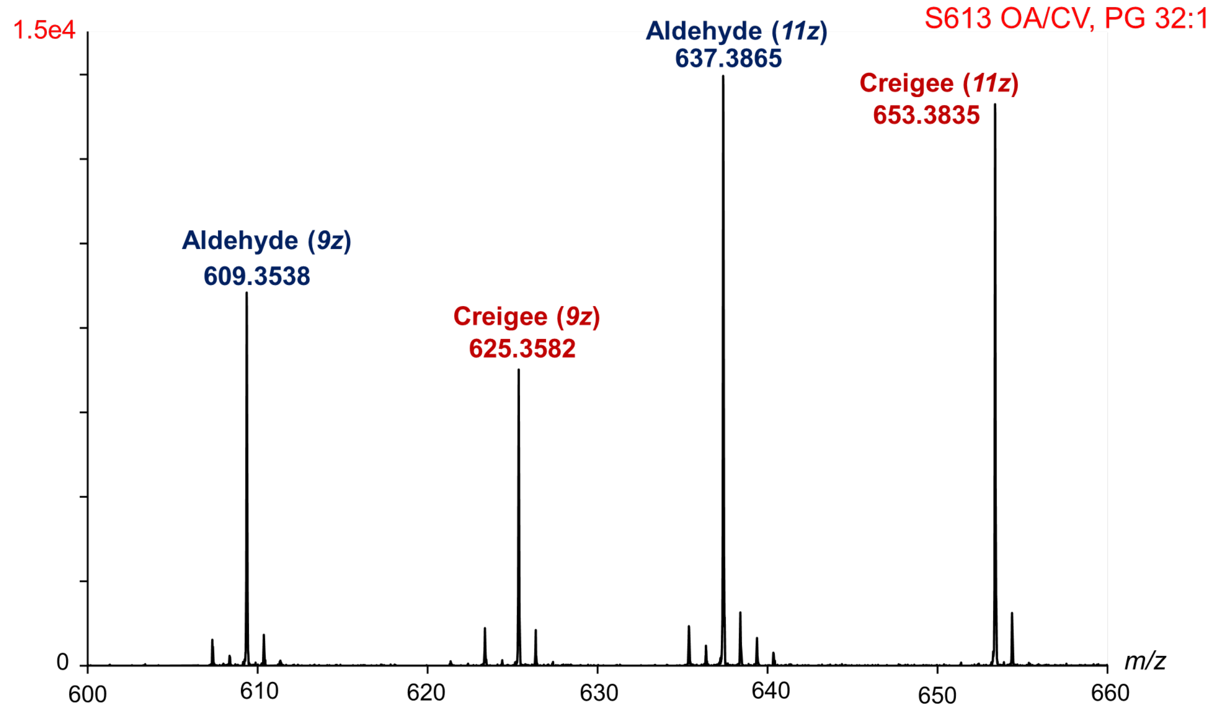

Figure S15i. OzID fragmentation of PG 32:1 in condition S613 OA/CV.

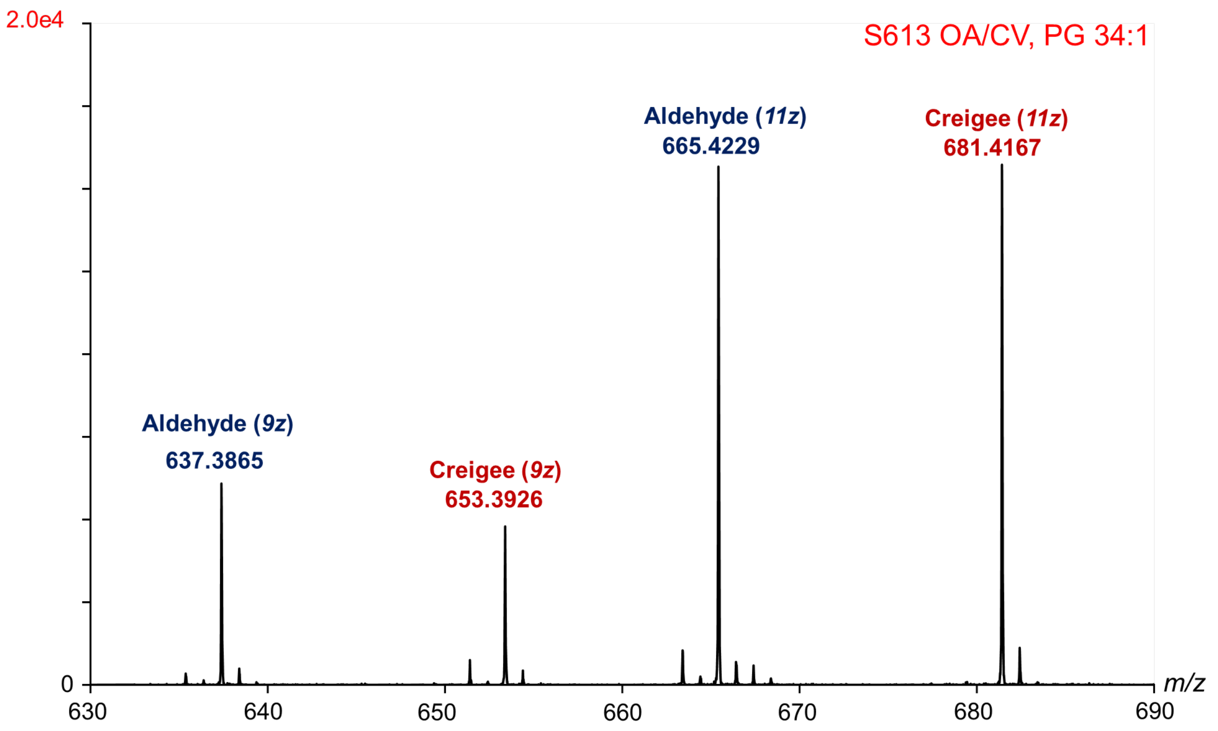

Figure S15j. OzID fragmentation of PG 34:1 in condition S613 OA/CV.

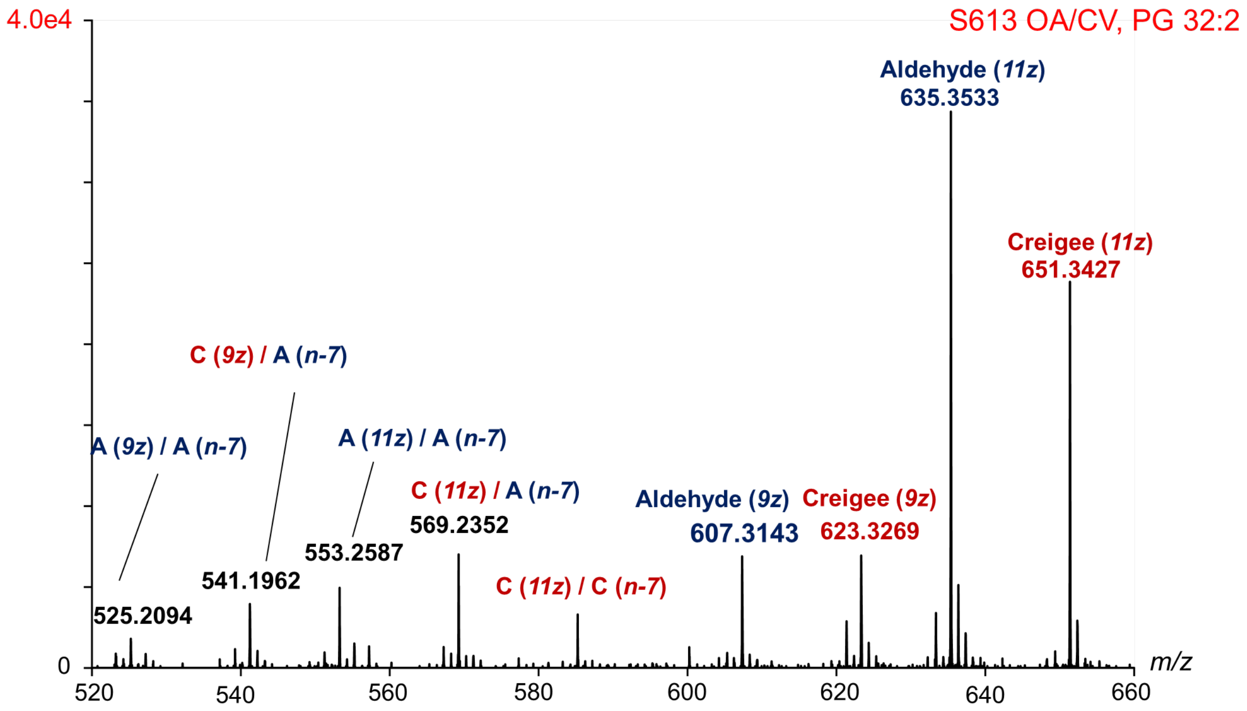

Figure S15k. OzID fragmentation of PG 32:2 in condition S613 OA/CV.

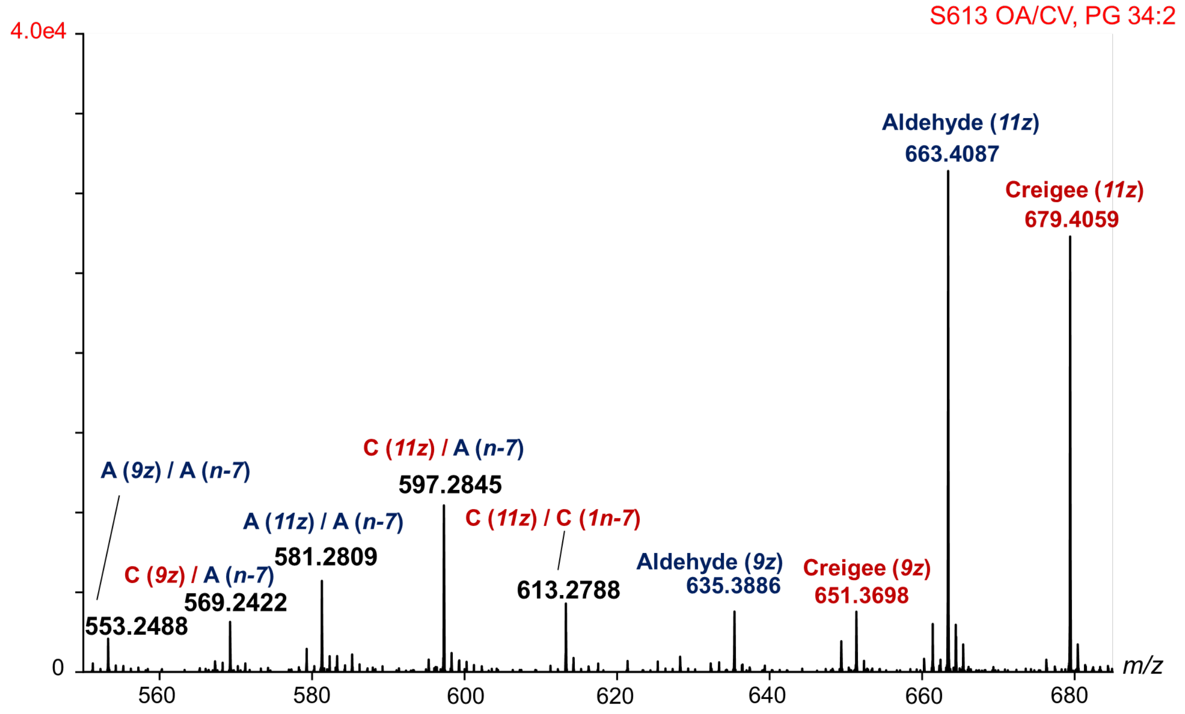

Figure S15l. OzID fragmentation of PG 34:2 in condition S613 OA/CV.

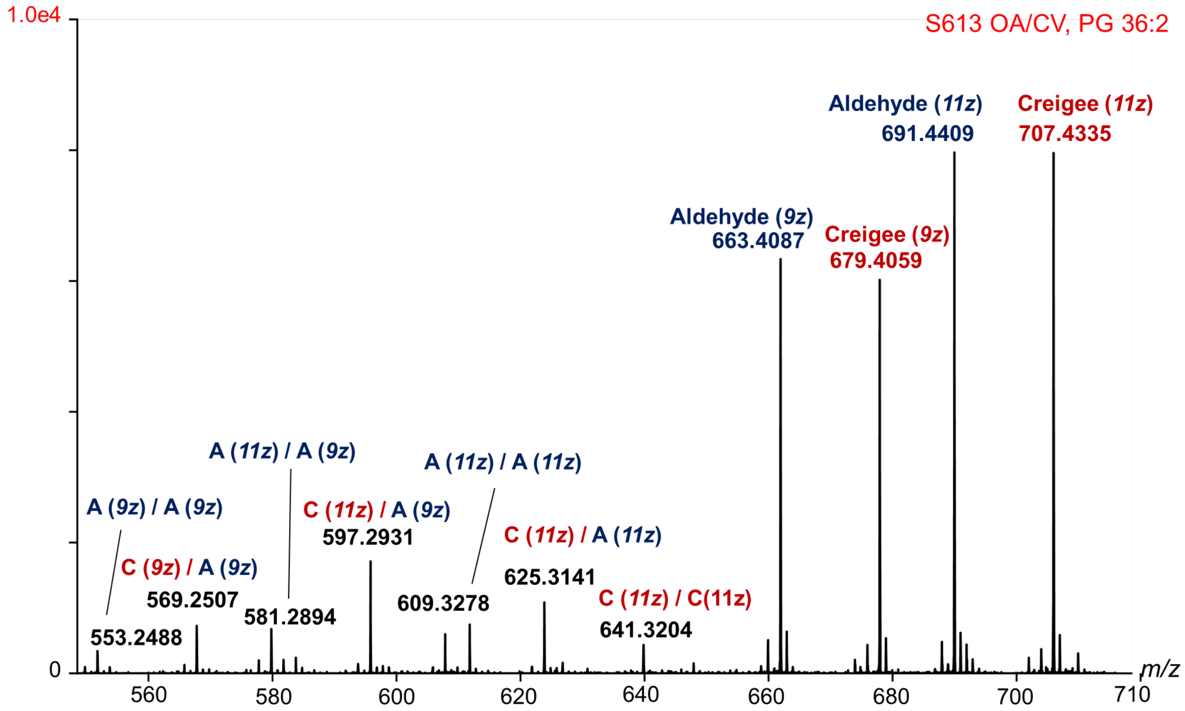

Figure S15m. OzID fragmentation of PG 36:2 in condition S613 OA/CV.

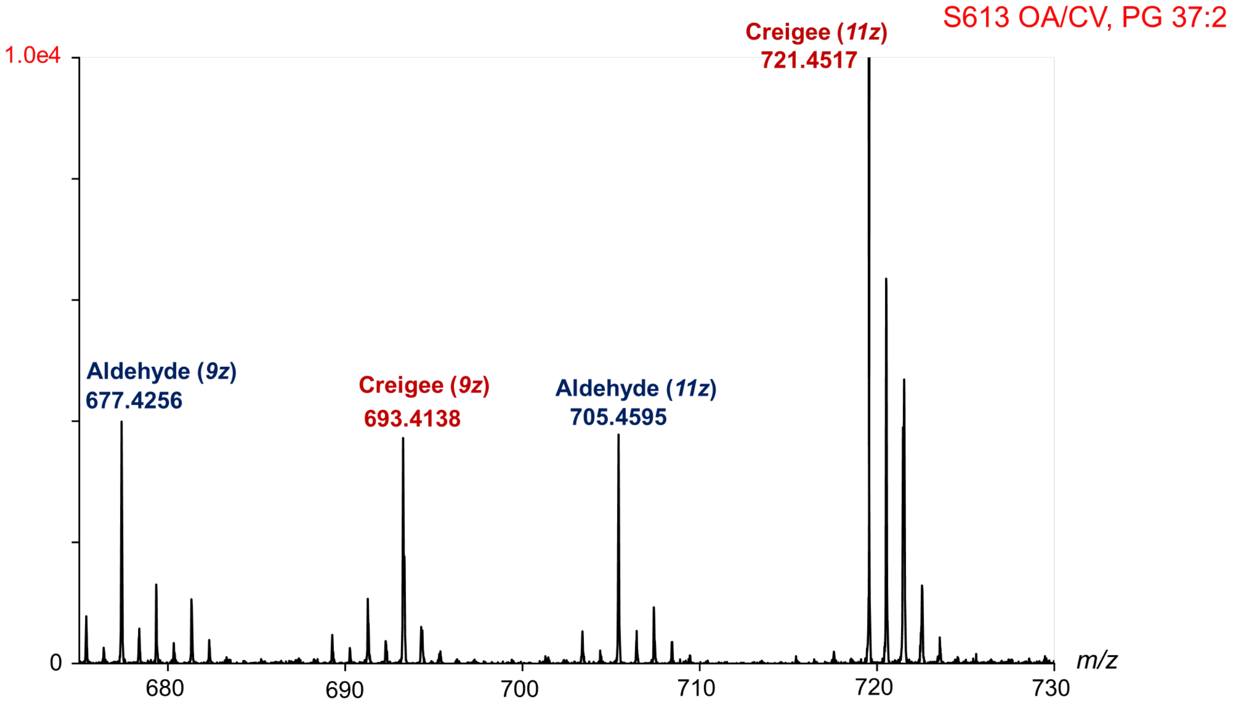

Figure S15n. OzID fragmentation of PG 37:2 in condition S613 OA/CV.

Table S20. Summary table of the ratio of PGs detected with only FA 18:1(*11z*), one FA 18:1(*9z*), or two FA 18:1(*9z*) based on the ozonolysis MS/MS displayed in *SI Figure 15.*

Condition	S613 CV	S613 OA	S613 OA/CV	R712 CV	R712 OA	R712 OA/CV
**PG 32:1-CV**	1	0.60	0.64	1	0.40	0.70
**PG 32:1-1 OA**	0	0.40	0.36	0	0.60	0.30
**PG 32:1-2 OA**	0	0	0	0	0	0
**PG 34:1-CV**	1	0.65	0.74	1	0.50	0.74
**PG 34:1-1 OA**	0	0.35	0.26	0	0.50	0.26
**PG 34:1-2 OA**	0	0	0	0	0	0
**PG 32:2-CV**	1	0.59	0.79	1	0.45	0.78
**PG 32:2-1 OA**	0	0.41	0.21	0	0.55	0.22
**PG 32:2-2 OA**	0	0	0	0	0	0
**PG 34:2-CV**	1	0.93	0.94	1	0.69	0.88
**PG 34:2-1 OA**	0	0.073	0.059	0	0.31	0.12
**PG 34:2-2 OA**	0	0	0	0	0	0
**PG 36:2-CV**	1	0.055	0.53	1	0.11	0.55
**PG 36:2-1 OA**	0	0.76	0.44	0	0.74	0.42
**PG 36:2-2 OA**	0	0.18	0.024	0	0.15	0.03
**PG 37:2-CV**	1	0	0.53	1	0	0.51
**PG 37:2-1 OA**	0	1	0.47	0	1	0.49
**PG 37:2-2 OA**	0	0	0	0	0	0

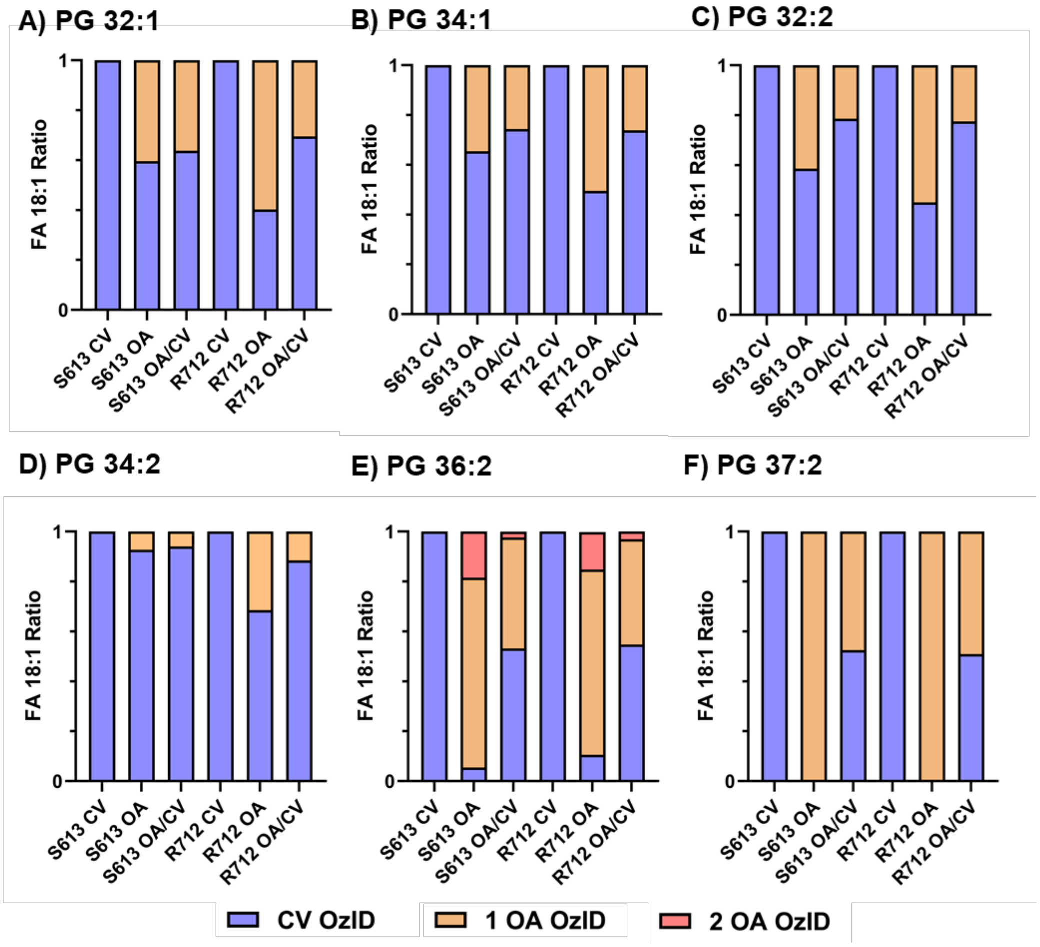

Figure S16: Ratio summary adapted from *SI Table S20* of PG OzID fragments corresponding to FA 18:1(*11z*) and FA 18:1(*9z*).

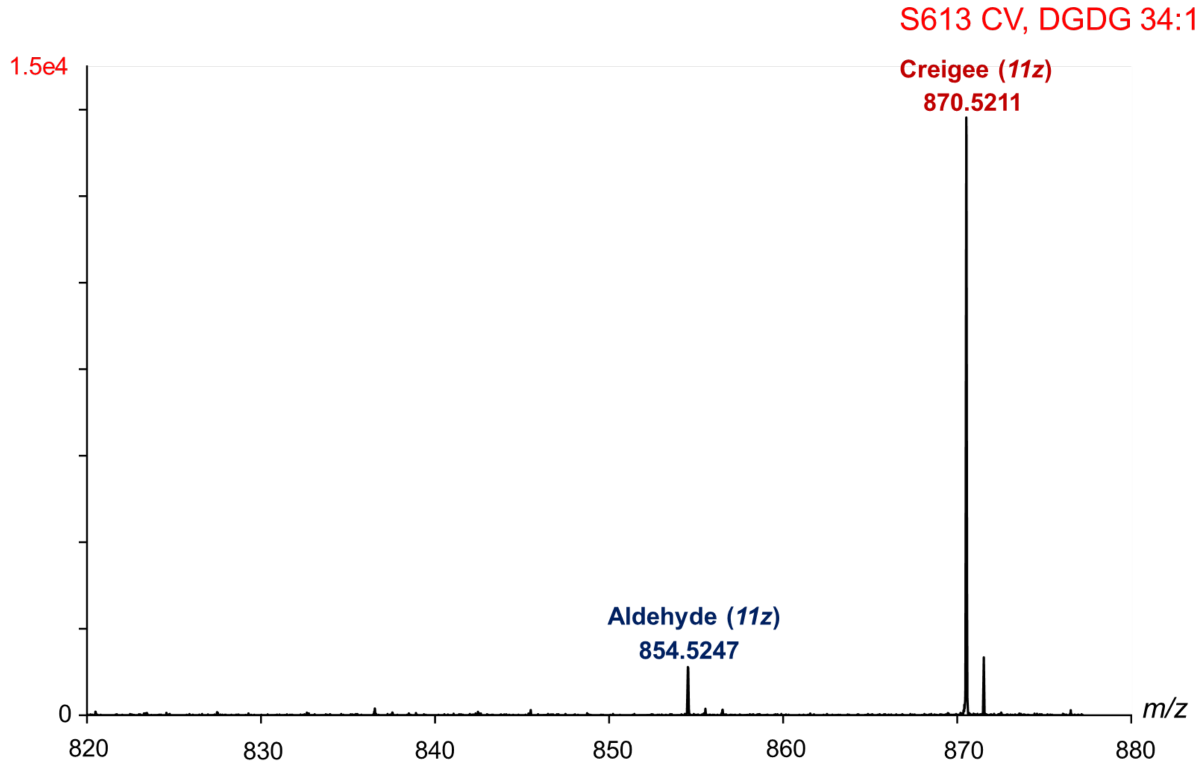

Figure S17a. OzID fragmentation of DGDG 34:1 in condition S613 CV.

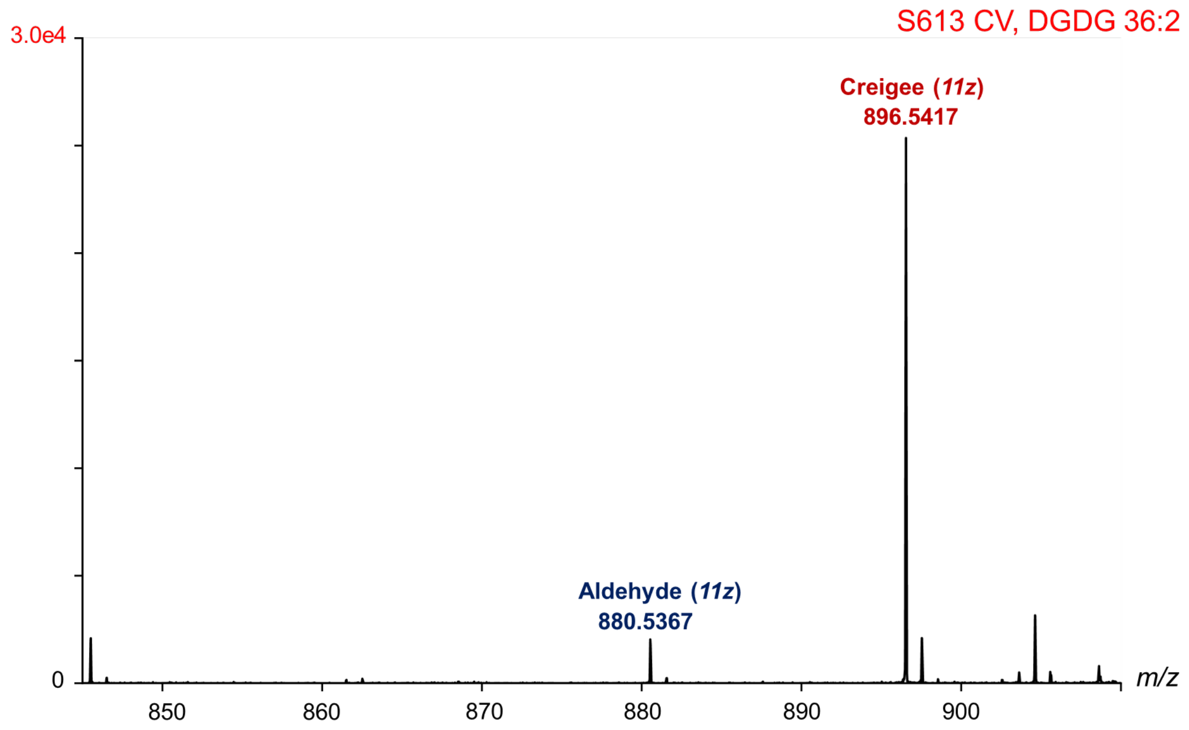

Figure S17b. OzID fragmentation of DGDG 36:2 in condition S613 CV.

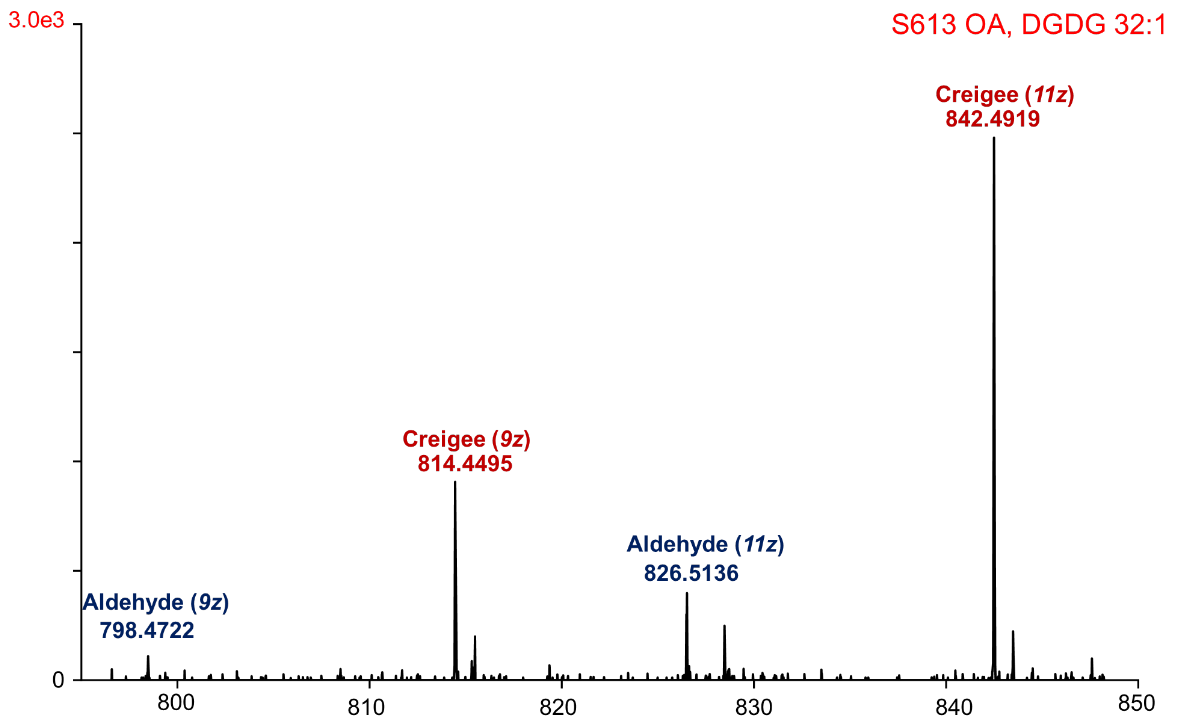

Figure S17c. OzID fragmentation of DGDG 37:2 in condition S613 CV.

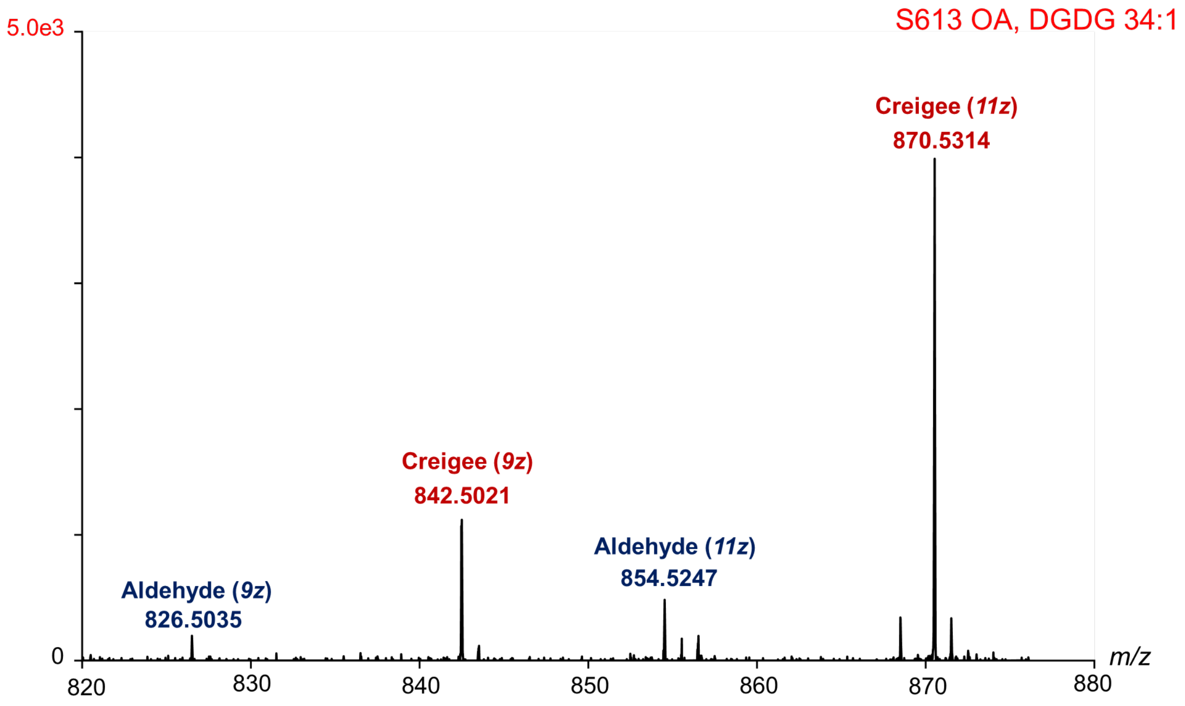

Figure S17d. OzID fragmentation of DGDG 34:1 in condition S613 OA.

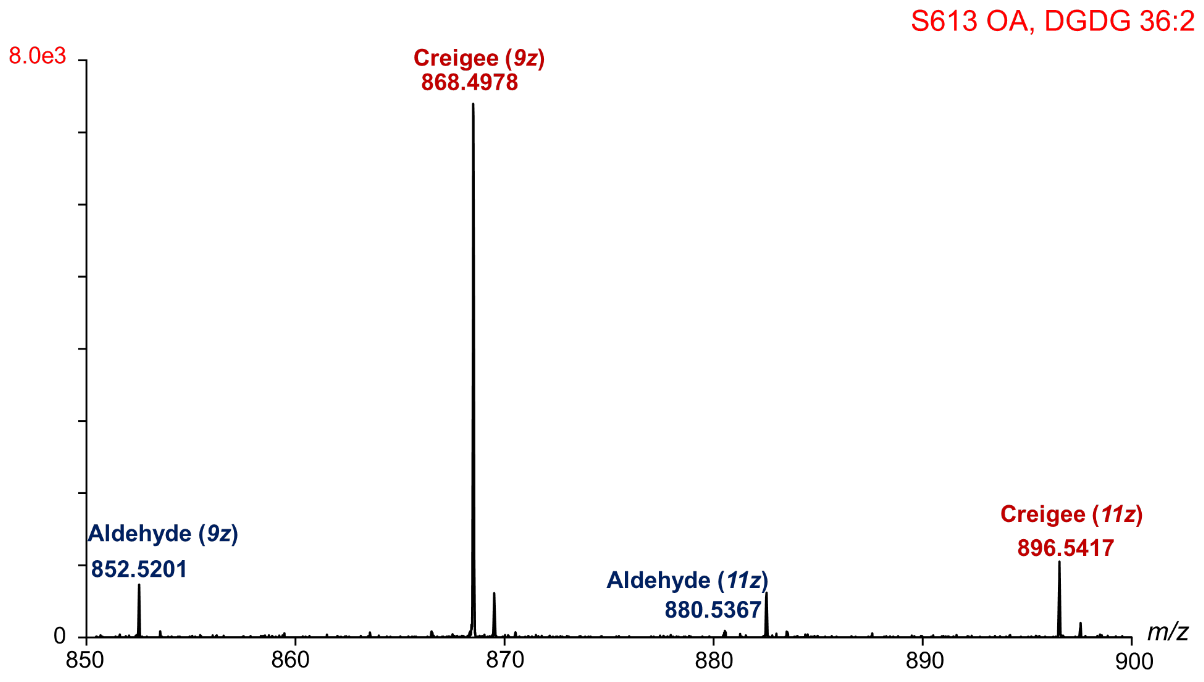

Figure S17e. OzID fragmentation of DGDG 36:2 in condition S613 OA.

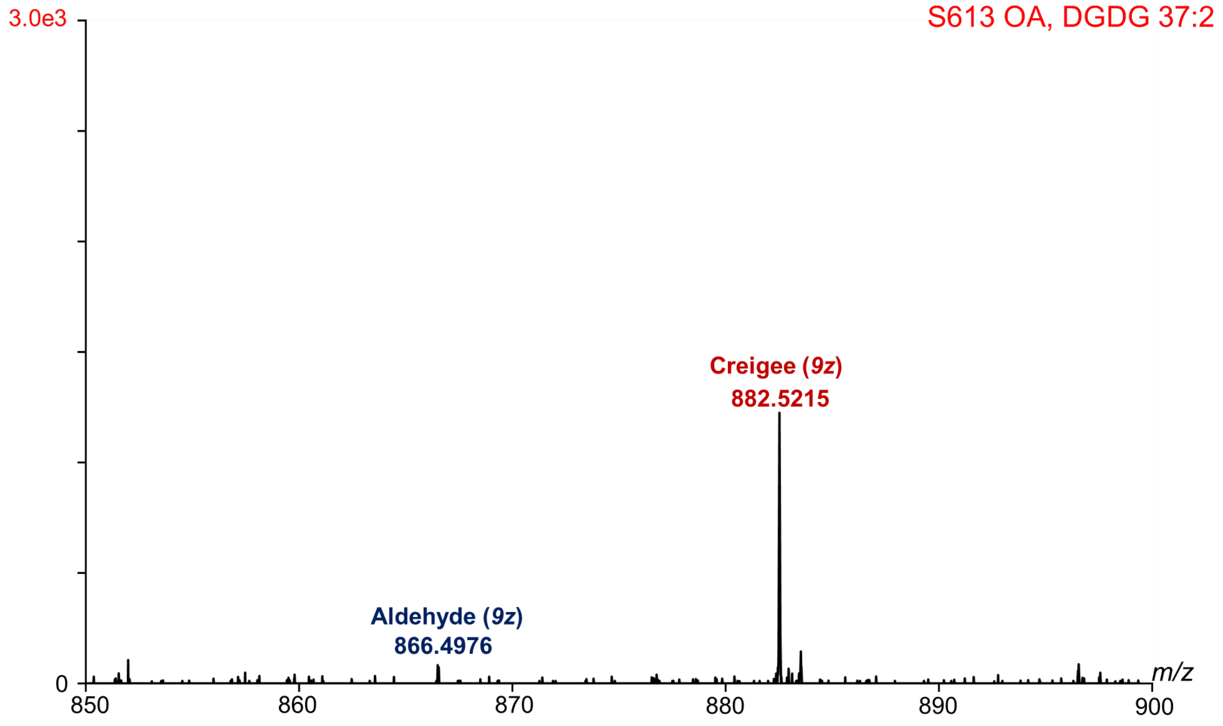

Figure S17f. OzID fragmentation of DGDG 37:2 in condition S613 OA.

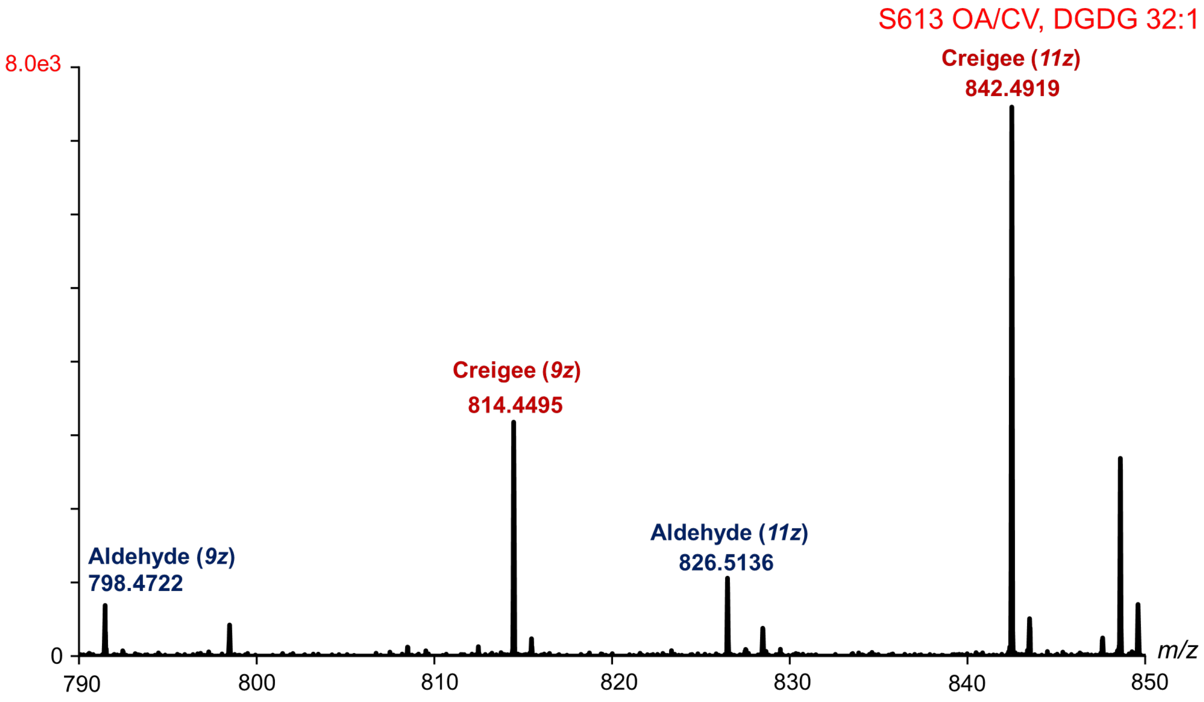

Figure S17g. OzID fragmentation of DGDG 32:1 in condition S613 OA/CV.

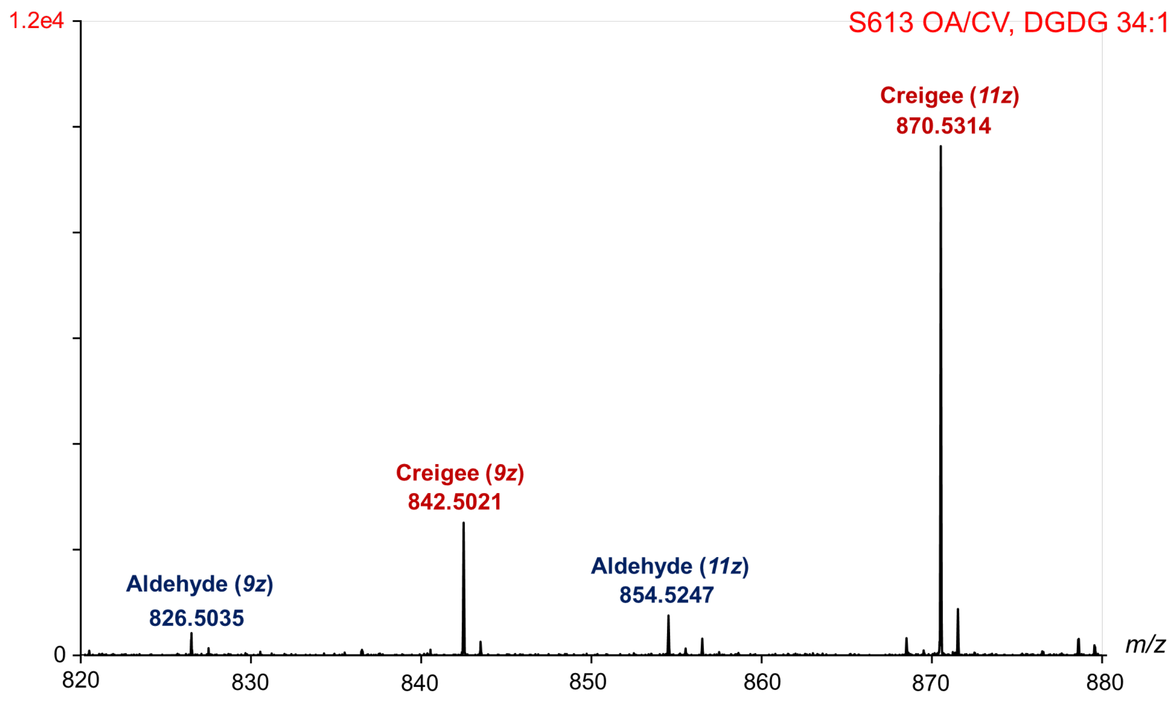

Figure S17h. OzID fragmentation of DGDG 34:1 in condition S613 OA/CV.

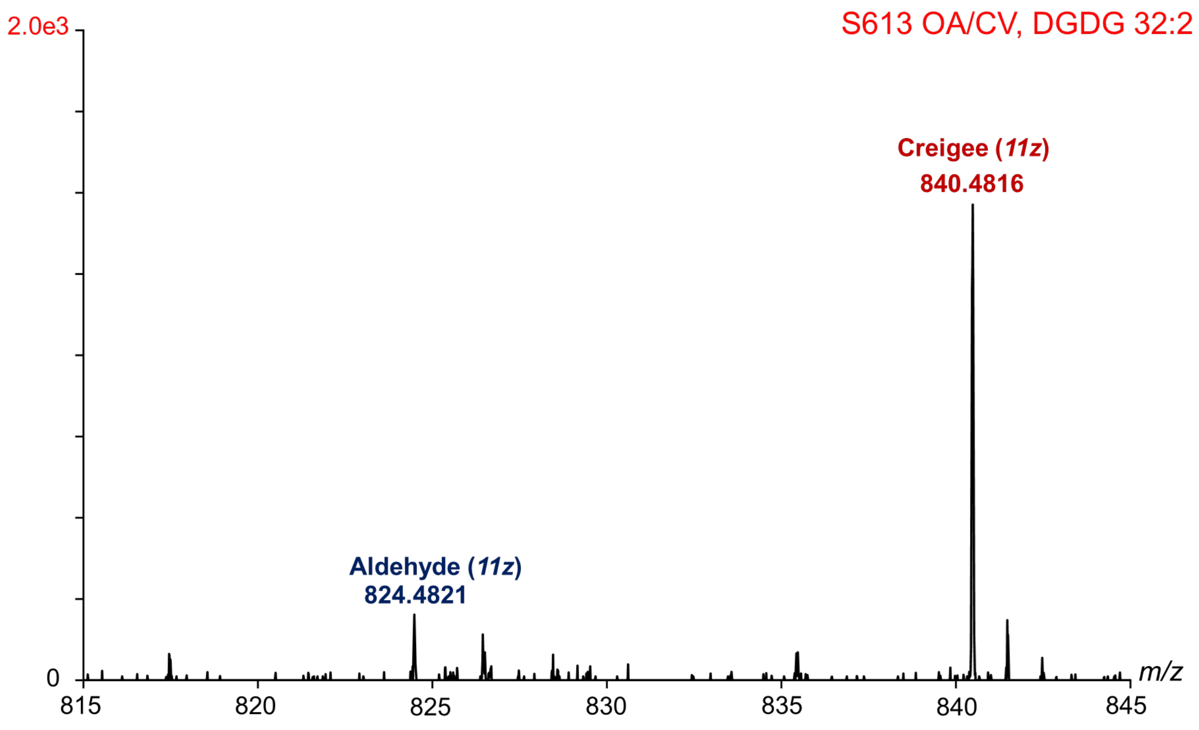

Figure S17i. OzID fragmentation of DGDG 32:2 in condition S613 OA/CV.

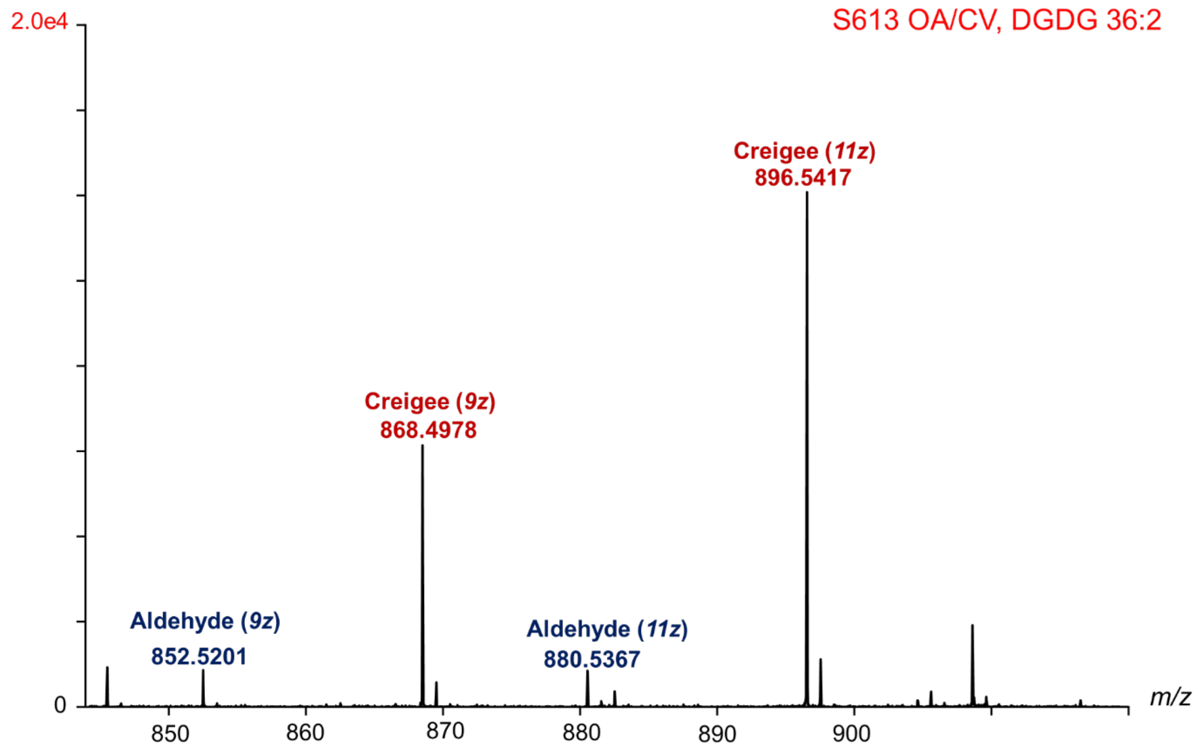

Figure S17j. OzID fragmentation of DGDG 36:2 in condition S613 OA/CV

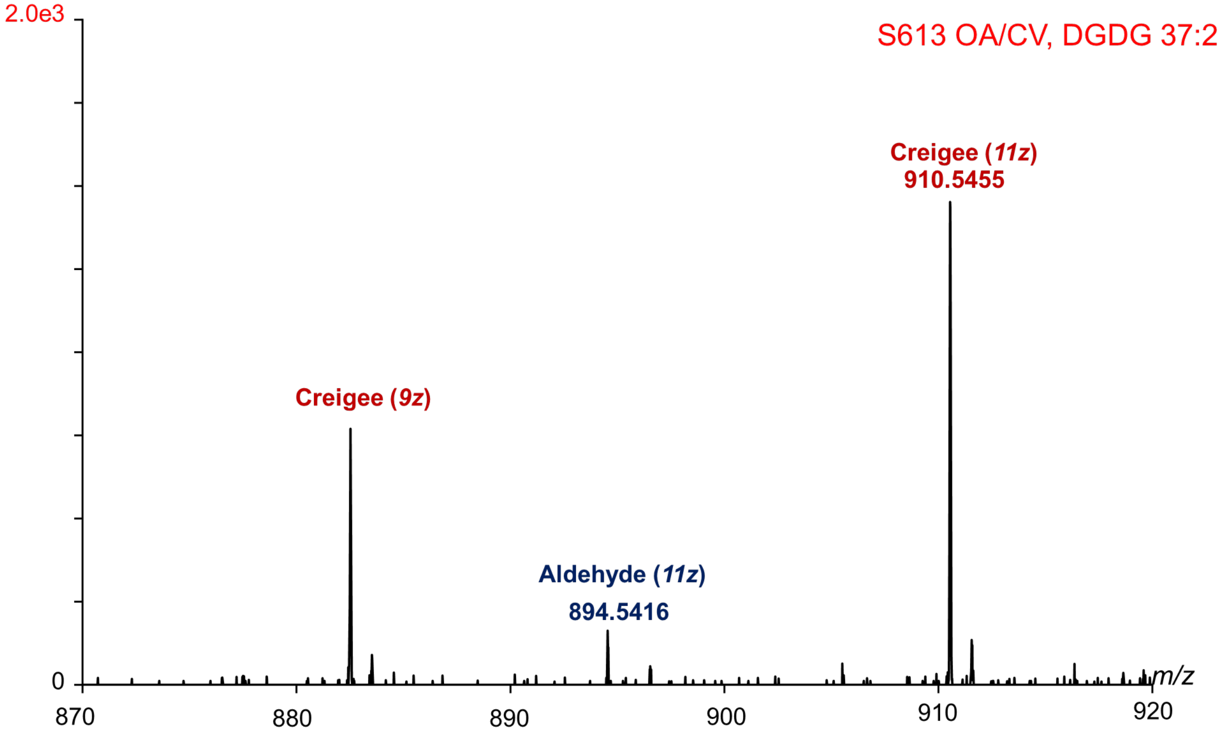

Figure S17k. OzID fragmentation of DGDG 37:2 in condition S613 OA/CV.

Table S21. Summary table of the ratio of DGDGs detected with only FA 18:1(*11z*), one FA 18:1(*9z*), or two FA 18:1(*9z*) based on the OzID MS/MS displayed in *SI Figure 17*.

Condition	S613 CV	S613 OA	S613 OA/CV	R712 CV	R712 OA	R712 OA/CV
**DGDG 32:1-CV**	1	0.74	0.71	1	0.50	0.78
**DGDG 32:1-1 OA**	0	0.26	0.29	0	0.50	0.22
**DGDG 32:1-2 OA**	0	0	0	0	0	0
**DGDG 34:1-CV**	1	0.78	0.78	1	0.69	0.84
**DGDG 34:1-1 OA**	0	0.22	0.22	0	0.31	0.16
**DGDG 34:1-2 OA**	0	0	0	0	0	0
**DGDG 32:2-CV**	1	0	1	1	0	0.88
**DGDG 32:2-1 OA**	0	0	0	0	0	0.12
**DGDG 32:2-2 OA**	0	0	0	0	0	0
**DGDG 34:2-CV**	0	0	1	1	1	1
**DGDG 34:2-1 OA**	0	0	0	0	0	0
**DGDG 34:2-2 OA**	0	0	0	0	0	0
**DGDG 36:2-CV**	1	0.12	0.66	1	0.17	0.65
**DGDG 36:2-1 OA**	0	0.88	0.34	0	0.83	0.35
**DGDG 36:2-2 OA**	0	0	0	0	0	0
**DGDG 37:2-CV**	1	0	0.67	1	0	0.73
**DGDG 37:2-1 OA**	0	1	0.33	0	1	0.27
**DGDG 37:2-2 OA**	0	0	0	0	0	0

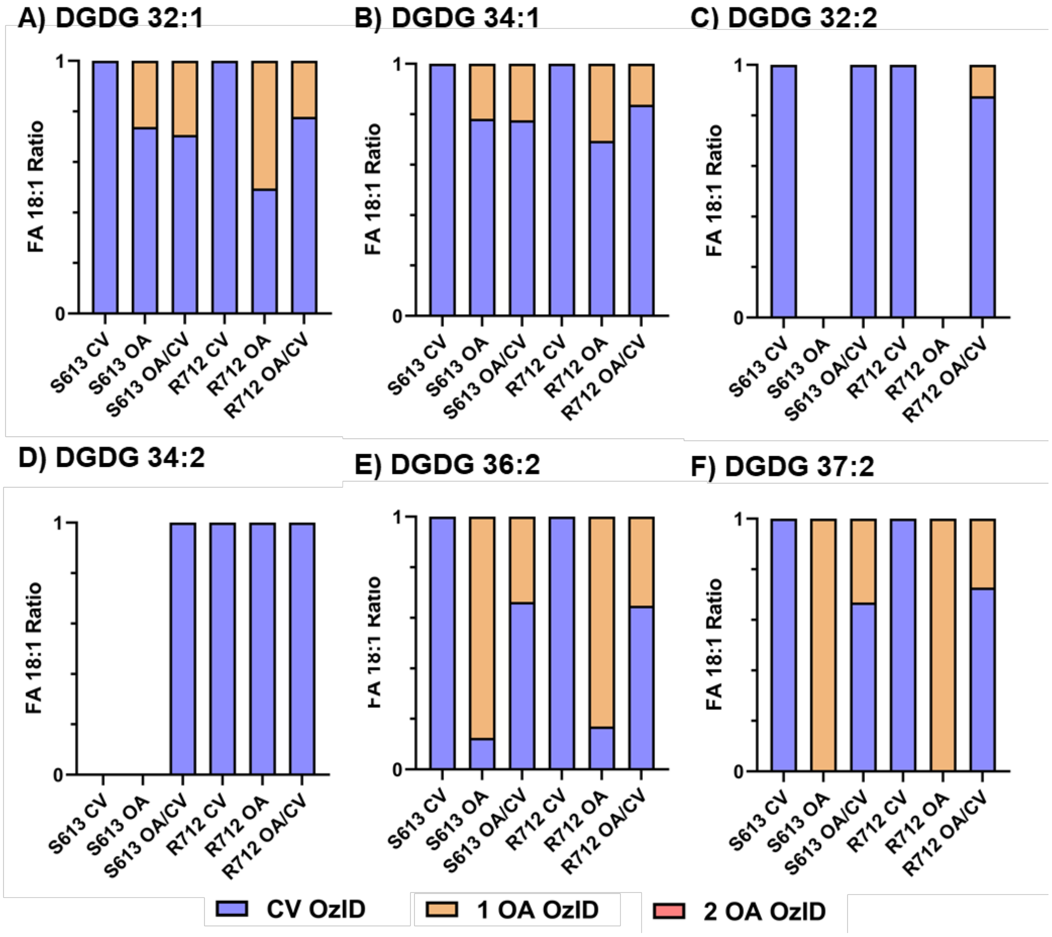

Figure S18: Ratio summary adapted from *SI Table S21* of DGDG OzID fragments corresponding to FA 18:1(*11z*) and FA 18:1(*9z*).

5c. Deuterium Labeling

Table S22. IDs and mass errors (ppm) of the un-labeled and labeled PGs and DGDGs by HILIC-IM-MS data set displayed in *Figures 5 and 6* and *SI Figures S19-S22.*

Compound	Adduct	RT	Mass	Exact Mass	Mass Error
**FFA 18:1**	$[M-H]^-$	0.71	281.2482	281.2486	-1.6
**d9-FFA 18:1**	$[M-H]^-$	0.71	290.3043	290.3046	-1.0
**10-HSA**	$[M-H]^-$	0.86	308.3150	308.3151	-0.3
**PG 32:1**	$[M-H]^-$	1.86	719.4878	719.4868	1.4
**d9-PG 32:1**	$[M-H]^-$	1.86	728.5443	728.5428	2.0
**PG 34:1**	$[M-H]^-$	1.86	747.5189	747.5181	1.1
**d9-PG 34:1**	$[M-H]^-$	1.83	756.5749	756.5741	1.0
**PG 35:1**	$[M-H]^-$	1.86	761.5333	761.5338	-0.6
**d9-PG 35:1**	$[M-H]^-$	1.83	770.5900	770.5898	0.3
**PG 32:2**	$[M-H]^-$	1.86	717.4729	717.4712	2.3
**d9-PG 32:2**	$[M-H]^-$	1.83	726.5289	726.5272	2.3
**PG 34:2**	$[M-H]^-$	1.83	745.5044	745.5025	2.6
**d9-PG 34:2**	$[M-H]^-$	1.83	754.5613	754.5585	3.7
**PG 36:2**	$[M-H]^-$	1.83	773.5348	773.5338	1.3
**d9-PG 36:2**	$[M-H]^-$	1.79	782.5911	782.5898	1.6
**2d9-PG 36:2**	$[M-H]^-$	1.79	791.6476	791.6458	2.3
**PG 37:2**	$[M-H]^-$	1.83	787.5502	787.5494	1.0
**d9-PG 37:2**	$[M-H]^-$	1.79	796.6065	796.6054	1.4
**2d9-PG 37:2**	$[M-H]^-$	1.79	805.6632	805.6614	2.2
**DGDG 32:1**	$[M+HCOO]^-$	1.14	935.5972	935.5949	2.4
**DGDG 34:1**	$[M+HCOO]^-$	1.09	963.6282	963.6262	2.0
**d9-DGDG 34:1**	$[M+HCOO]^-$	1.09	972.6820	972.6822	-0.2
**DGDG 35:1**	$[M+HCOO]^-$	1.09	977.5888	977.6418	-54.2
**d9-DGDG 35:1**	$[M+HCOO]^-$	1.09	986.6949	986.6978	-2.9
**DGDG 34:2**	$[M+HCOO]^-$	1.09	961.6153	961.6105	5.0
**d9-DGDG 34:2**	$[M+HCOO]^-$	1.09	970.6647	970.6665	-1.8
**DGDG 36:2**	$[M+HCOO]^-$	1.09	989.6455	989.6418	3.7
**2d9-DGDG 36:2**	$[M+HCOO]^-$	1.05	1007.7556	1007.7538	1.8
**DGDG 37:2**	$[M+HCOO]^-$	1.09	1003.6569	1003.6575	-0.6
**d9-DGDG 37:2**	$[M+HCOO]^-$	1.05	1012.7147	1012.7135	1.2
**2d9-DGDG 37:2**	$[M+HCOO]^-$	1.05	1021.7688	1021.7695	-0.7

Table S23. Summary table of the decimal ratios of unlabeled PGs and their corresponding *d9*-PGs (with either one or 2 *d9*-FA 18:1(*9z*) tails), based on the species detected in *SI Table S22*.

Lipid Species	S613 CV	S613 OA	S613 OA/CV	R712 CV	R712 OA	R712 OA/CV
**PG 32:1**	0.999	0.604	0.62	0.98	0.40	0.61
**PG 32:1 d9**	0.0006	0.37	0.38	0.0185	0.60	0.39
**PG 34:1**	0.996	0.63	0.72	0.989	0.48	0.69
**PG 34:1 d9**	0.004	0.37	0.28	0.011	0.52	0.31
**PG 35:1**	0.996	0.45	0.54	0.991	0.37	0.50
**PG 35:1 d9**	0.00384	0.55	0.46	0.009	0.63	0.50
**PG 32:2**	0.999	0.40	0.51	0.92	0.26	0.53
**PG 32:2 d9**	0.0012	0.60	0.49	0.08	0.74	0.47
**PG 34:2**	0.9997	0.68	0.75	0.9997	0.50	0.69
**PG 34:2 d9**	0.0003	0.32	0.25	0.0003	0.50	0.31
**PG 36:2**	0.9996	0.072	0.31	0.9999	0.073	0.32
**PG 36:2 d9**	<0.0001	0.041	0.46	<0.0001	0.077	0.45
**PG 36:2 2-d9**	0.00033	0.887	0.23	<0.0001	0.85	0.23
**PG 37:2**	0.9996	0.014	0.11	0.9999	0.021	0.12
**PG 37:2 d9**	0.0002	0.022	0.44	<0.0001	0.043	0.45
**PG 37:2 2-d9**	0.0002	0.9642	0.45	<0.0001	0.94	0.43

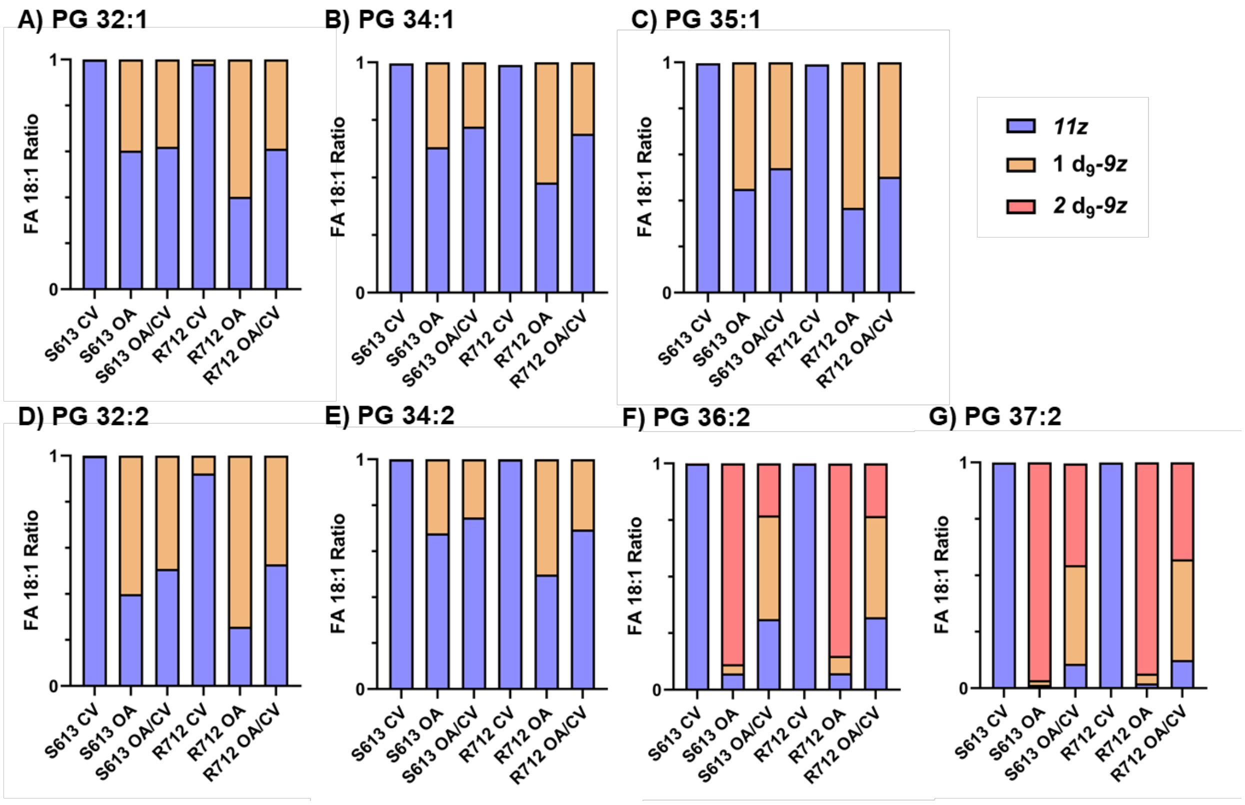

Figure S19: FA 18:1(*11z*) and FA 18:1(*9z*) ratios from PGs based on the deuterium labeled oleic acid experiment adapted from *SI Table S23*.

Table S24. Summary table of the decimal ratios of unlabeled DGDGs and their corresponding *d9*-DGDGs (with either one or 2 *d9*-FA 18:1(*9z*) tails), based on the species detected in *SI Table S22*.

Lipid Species	S613 CV	S613 OA	S613 OA/CV	R712 CV	R712 OA	R712 OA/CV
**DGDG 34:1**	0.994	0.67	0.73	0.98	0.57	0.70
**DGDG 34:1 d9**	0.006	0.33	0.27	0.02	0.43	0.30
**DGDG 35:1**	0.999	0.66	0.69	0.991	0.53	0.68
**DGDG 35:1 d9**	0.001	0.34	0.31	0.009	0.47	0.32
**DGDG 34:2**	0.993	0.82	0.82	0.996	0.67	0.75
**DGDG 34:2 d9**	0.007	0.18	0.18	0.004	0.33	0.25
**DGDG 36:2**	0.998	0.09	0.65	0.996	0.18	0.63
**DGDG 36:2 2-d9**	0.002	0.91	0.34	0.004	0.82	0.36
**DGDG 37:2**	0.9984	0.018	0.23	0.997	0.050	0.26
**DGDG 37:2 d9**	0.00064	0.043	0.45	0.001	0.063	0.42
**DGDG 37:2 2-d9**	0.001	0.94	0.32	0.002	0.89	0.33

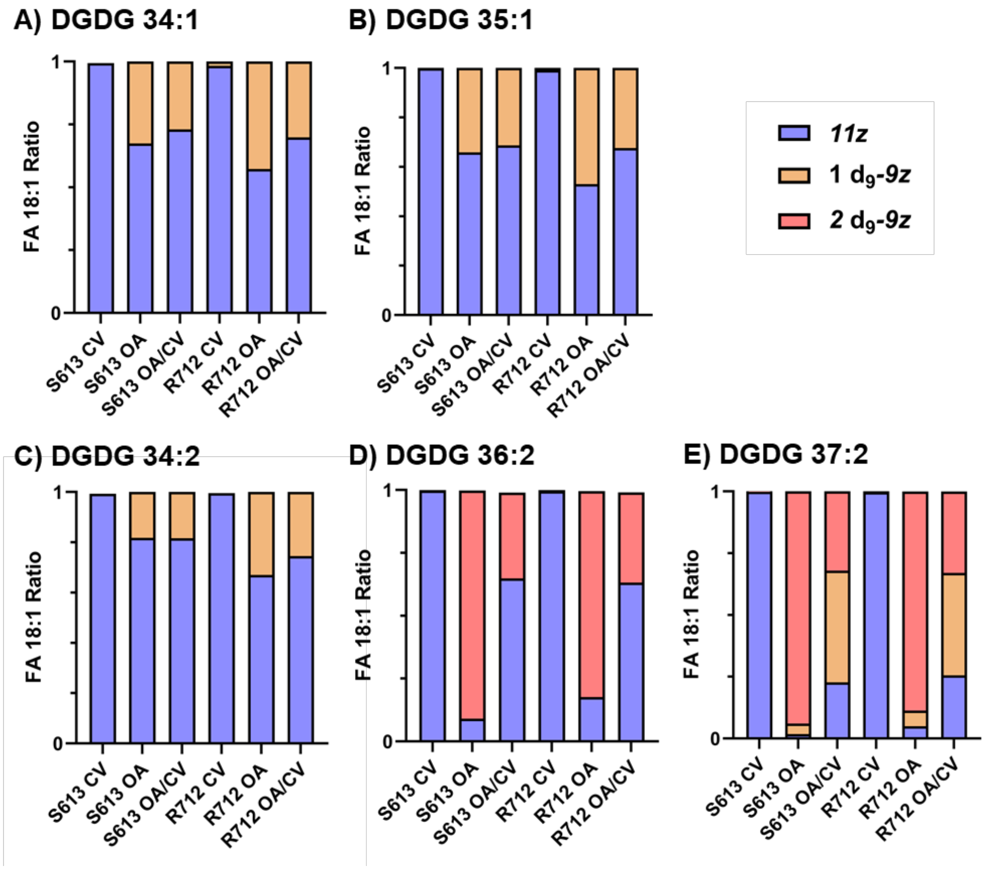

Figure S20: FA 18:1(*11z*) and FA 18:1(*9z*) ratios from DGDGs based on the deuterium labeled oleic acid experiment adapted from *SI Table S24*.

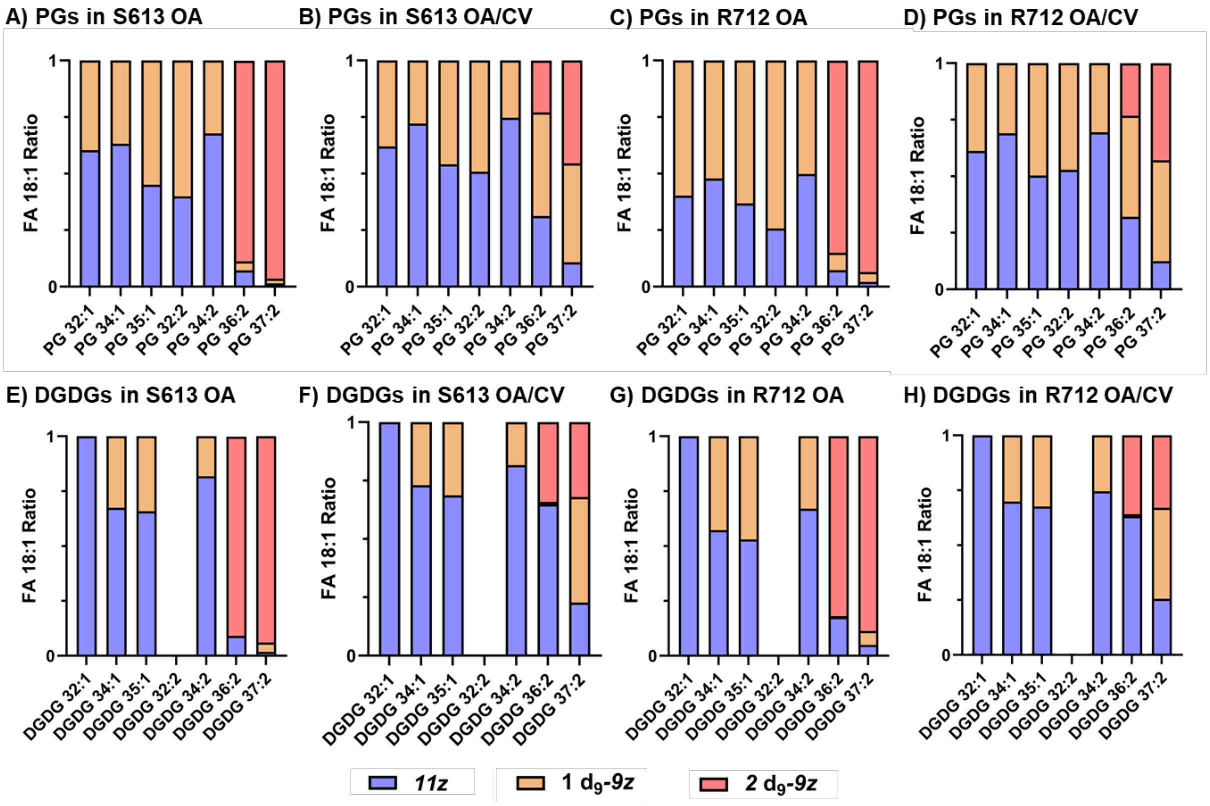

Figure S21: FA 18:1(*11z*) and FA 18:1(*9z*) ratios from PGs and DGDGs based on each condition from the deuterium labeled oleic acid experiment.

Table S25. Summary table of the decimal ratios of total unlabeled PGs and DGDGs and their corresponding *d9*-PGs and -DGDGs (with either one or 2 *d9*-FA 18:1(*9z*) tails), based on the species detected in *SI Table S21*.

	**PGs**	**1 d9-PGs**	**2 d9-PGs**	**DGDGs**	**1 d9-DGDGs**	**2 d9-DGDGs**
**S613 CV**	0.998	0.002	<0.0001	0.995	0.0043	0.0006
**S613 OA**	0.32	0.224	0.462	0.32	0.10	0.57
**S613 OA/CV**	0.52	0.39	0.1	0.672	0.204	0.124
**R712 CV**	0.9889	0.0111	<0.0001	0.99	0.01	0.001
**R712 OA**	0.26	0.34	0.40	0.355	0.162	0.483
**R712 OA/CV**	0.515	0.396	0.0894	0.65	0.22	0.13

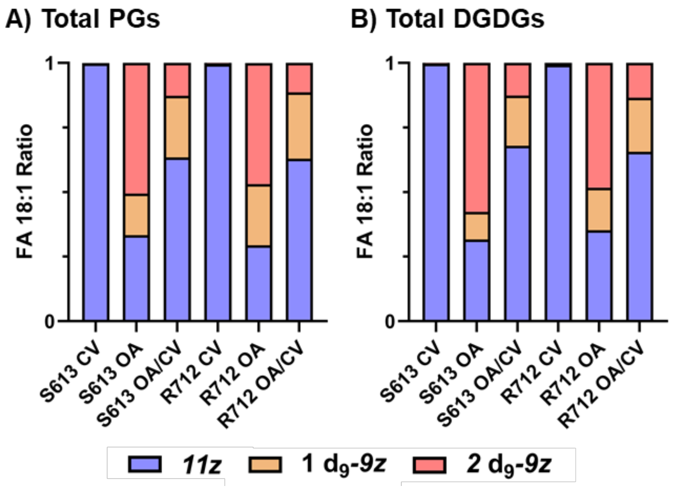

Figure S22. Distribution of FA 18:1(*11z*) and incorporated d_9-FA 18:1(*9z*) utilized in the sum production of lipid classes A) PGs and B) DGDGs adapted from *SI Table S25*.

6. Daptomycin Growth Curves

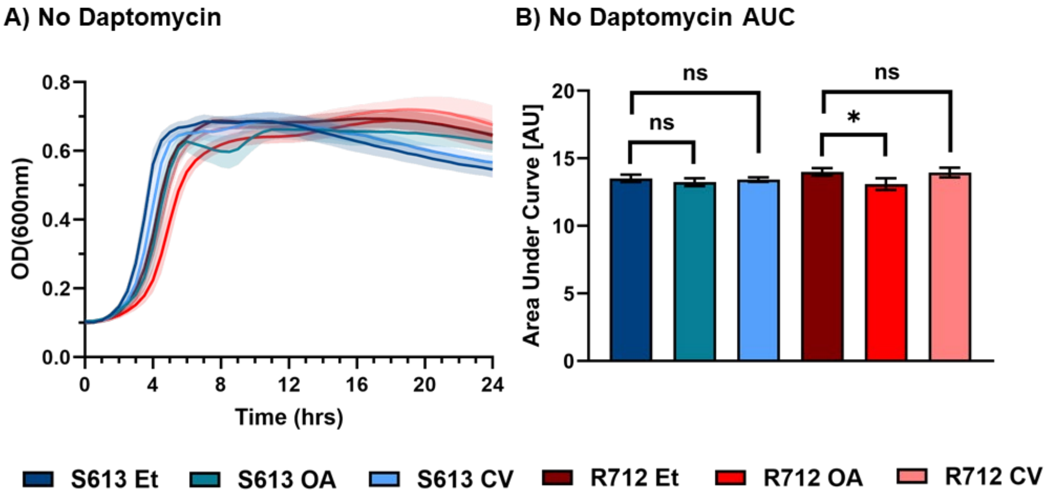

Figure S23. A) growth curves of S613 and R712 with Et, OA, CV without the challenge of daptomycin and B) the corresponding area under the curve (<u>Note:</u> The curves are an average of 3 trials with 5 replicates (per trial) of each condition; unpaired *t*-test *p*-value where *<0.05).

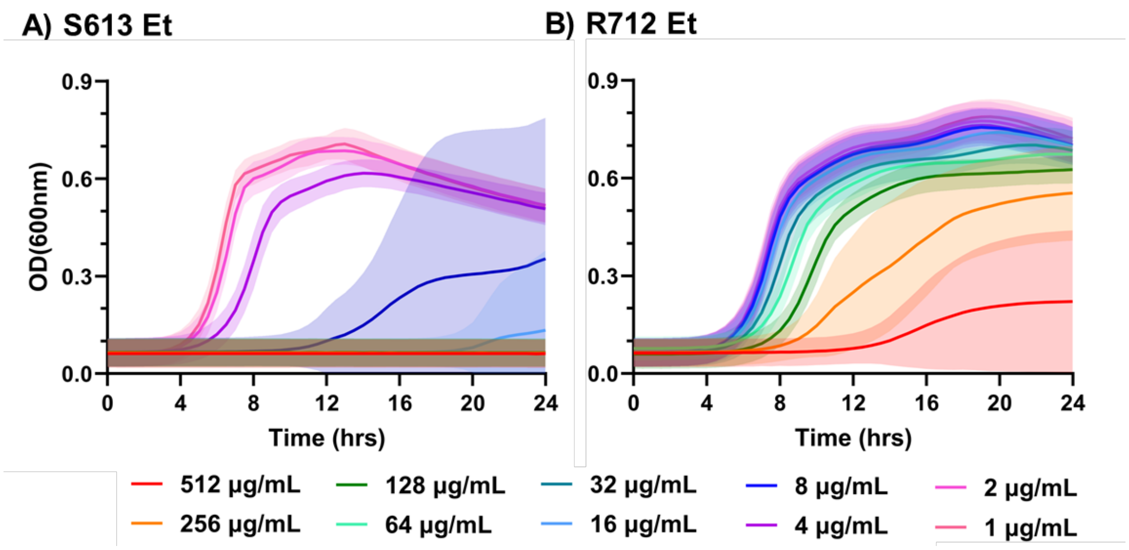

Figure S24. A) growth curves of S613 Et with increasing concentrations of daptomycin and B) growth curves of R712 Et with increasing concentrations of daptomycin (Note: The curves are an average of 6 trials with 2 replicates (per trial) of each condition).

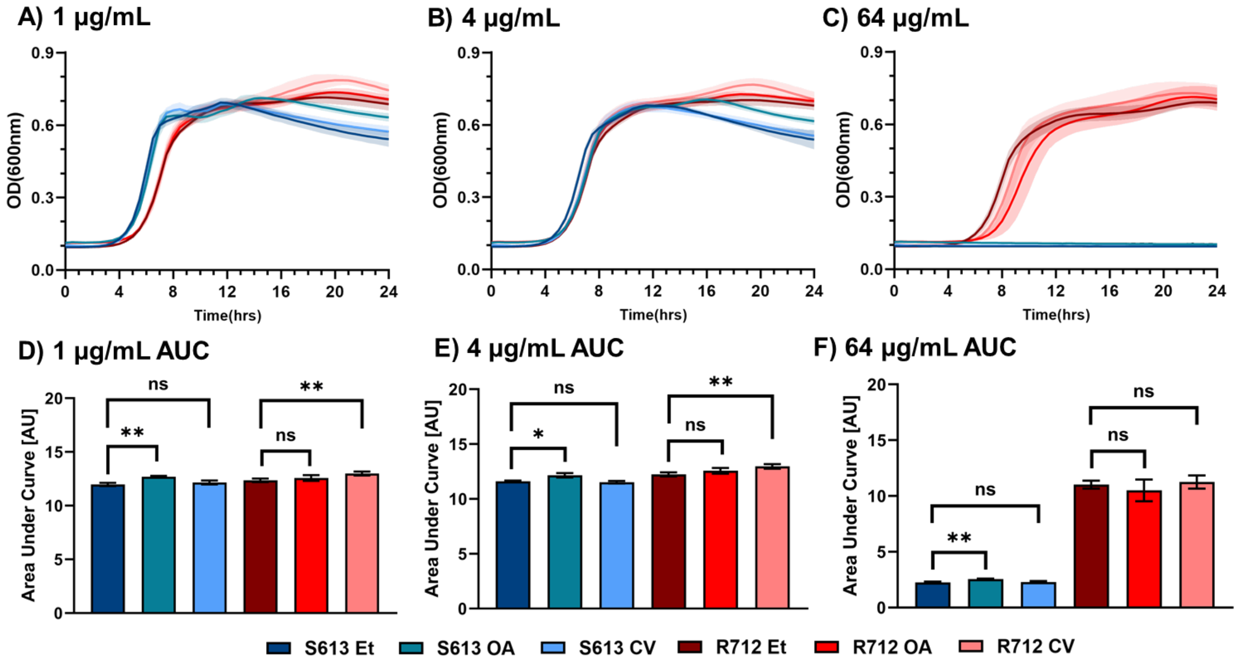

Figure S25. A) growth curves with 1 µg/mL daptomycin, B) growth curves with 4 µg/mL daptomycin, C) growth curves with 64 µg/mL daptomycin, D) area under the curve of 1 µg/mL daptomycin, E) area under the curve of 4 µg/mL daptomycin, and F) area under the curve of 64 µg/mL daptomycin (<u>Note</u>: The curves are an average of 3 trials with 5 replicates (per trial) of each condition; false-discovery rate adjusted *p*-values from multiple unpaired *t*-tests where *<0.05, **<.01, ***<0.001).

Table S26. Summary table of the unpaired *t*-test *p*-values (with false discovery rate adjustment to the multiple comparison daptomycin growth curve trials) were performed on the area under the growth curves displayed in *SI Figure S23 and S25.*

Dap Concentration	Condition 1	Condition 2	P-Value
**No Dap**	S613 Et	S613 OA	0.30
**No Dap**	S613 Et	S613 CV	0.65
**No Dap**	R712 Et	R712 OA	0.039
**No Dap**	R712 Et	R712 CV	0.90
**No Dap**	S613 Et	R712 Et	0.10
**No Dap**	R712 Et	S613 OA	0.033
**1 µg/mL**	S613 Et	S613 OA	0.0035
**1 µg/mL**	S613 Et	S613 CV	0.48
**1 µg/mL**	R712 Et	R712 OA	0.46
**1 µg/mL**	R712 Et	R712 CV	0.0065
**1 µg/mL**	S613 Et	R712 Et	0.044
**1 µg/mL**	R712 Et	S613 OA	0.039
**4 µg/mL**	S613 Et	S613 OA	0.011
**4 µg/mL**	S613 Et	S613 CV	0.48
**4 µg/mL**	R712 Et	R712 OA	0.46
**4 µg/mL**	R712 Et	R712 CV	0.0065
**4 µg/mL**	S613 Et	R712 Et	0.0082
**4 µg/mL**	R712 Et	S613 OA	0.43
**64 µg/mL**	S613 Et	S613 OA	0.0035
**64 µg/mL**	S613 Et	S613 CV	0.52
**64 µg/mL**	R712 Et	R712 OA	0.46
**64 µg/mL**	R712 Et	R712 CV	0.21
**64 µg/mL**	S613 Et	R712 Et	<0.0001
**64 µg/mL**	R712 Et	S613 OA	<0.0001

Table S27. Doubling times (mins) of growth curves displayed in *SI Figure S23 and S25* determined between two time points in the middle of the exponential phase.

Conditions	No Dap	1 µg/mL	4 µg/mL	64 µg/mL
**S613 Et**	29.12	26.68	23.69	N/A
**S613 OA**	24.63	22.66	22.10	N/A
**S613 CV**	26.85	24.70	23.01	N/A
**R712 Et**	23.69	22.96	22.08	18.75
**R712 OA**	22.05	23.12	21.49	18.05
**R712 CV**	22.94	22.81	23.06	20.22
